# Rift Valley fever: An open-source transmission dynamics simulation model

**DOI:** 10.1371/journal.pone.0209929

**Published:** 2019-01-09

**Authors:** Robert Sumaye, Famke Jansen, Dirk Berkvens, Bernard De Baets, Eveline Geubels, Etienne Thiry, Meryam Krit

**Affiliations:** 1 Ifakara Health Institute, Ifakara, Tanzania; 2 Institute of Tropical Medicine, Department of Biomedical Sciences, Antwerp, Belgium; 3 University of Liège, Faculty of Veterinary Sciences, Liège, Belgium; 4 Ghent University, Faculty of Bioscience Engineering, Department of Data Analysis and Mathematical Modelling, Ghent, Belgium; Faculty of Science, Ain Shams University (ASU), EGYPT

## Abstract

Rift Valley fever (RVF) is one of the major viral zoonoses in Africa, affecting humans and several domestic animal species. The epidemics in eastern Africa occur in a 5-15 year cycle coinciding with abnormally high rainfall generally associated to the warm phase of the El Niño event. However, recently, evidence has been gathered of inter-epidemic transmission. An open-source, easily applicable, accessible and modifiable model was built to simulate the transmission dynamics of RVF. The model was calibrated using data collected in the Kilombero Valley in Tanzania with people and cattle as host species and *Ædes mcintoshi*, *Æ. ægypti* and two *Culex* species as vectors. Simulations were run over a period of 27 years using standard parameter values derived from two previous studies in this region. Our model predicts low-level transmission of RVF, which is in line with epidemiological studies in this area. Emphasis in our simulation was put on both the dynamics and composition of vector populations in three ecological zones, in order to elucidate the respective roles played by different vector species: the model output did indicate the necessity of *Culex* involvement and also indicated that vertical transmission in *Ædes mcintoshi* may be underestimated. This model, being built with open-source software and with an easy-to-use interface, can be adapted by researchers and control program managers to their specific needs by plugging in new parameters relevant to their situation and locality.

## Introduction

Rift Valley fever (RVF) is caused by the Rift Valley fever virus (RVFv), which belongs to the genus *Phlebovirus* in the family Bunyaviridae. RVF is one of the major viral zoonoses in Africa, affecting man and several domestic animal species [1, 2].

**Fig 1:**
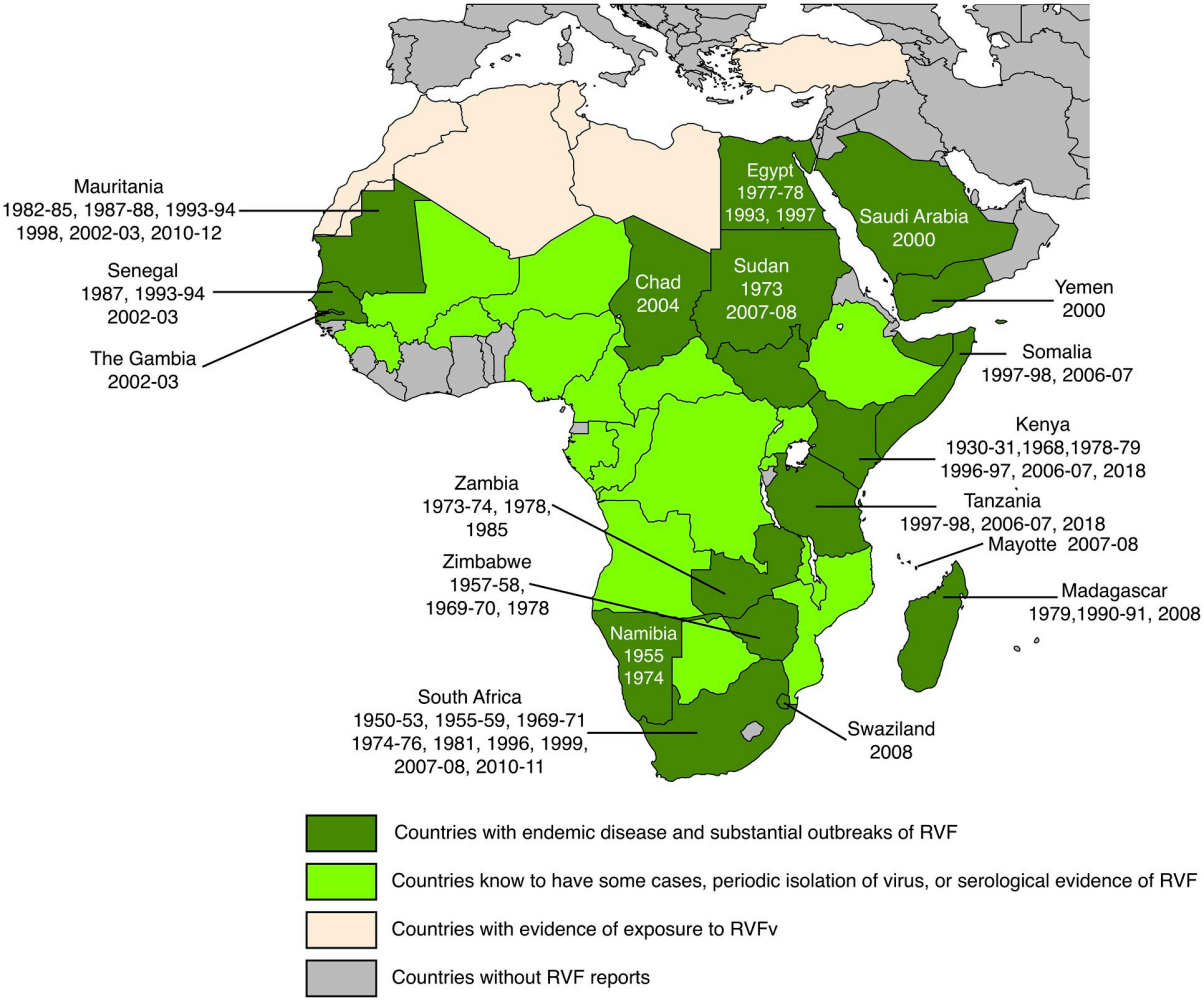
Geographical distribution of Rift Valley fever. The years indicate when the disease was detected in individual countries. Adapted from CDC and https://www.nature.com/articles/emi201381/figures/1 with supplementary information from [4–8]. Dark green: Chad, Egypt, Kenya, Madagascar, Mauritania, Mayotte (Fr.), Namibia, Saudi Arabia, Senegal, Somalia, South Africa, Sudan, Swaziland, Tanzania, The Gambia, Yemen, Zambia, Zimbabwe. Light green: Angola, Botswana, Burkina Faso, Cameroon, Central African Republic, Congo, Democratic Republic of the Congo, Ethiopia, Gabon, Guinea Conakry, Malawi, Mali, Mozambique, Niger, Nigeria, Rwanda, Uganda Light beige: Algeria, Libya, Morocco, Tunisia, Turkey.

A syndrome compatible with RVF was first described in the Rift Valley of Kenya in the early 1900s and the virus was isolated in the 1930s [3]. The known range of RVFv is shown in Fig 1. RVF was confined to eastern and southern Africa until about 1975. Since then it has expanded its range first to Egypt (1977), then to western Africa (*ca.* 1980) and finally to the Arabian peninsula in 2000 [4]. It has so far not been officially confirmed from the Maghreb countries, although there is at least serological evidence of import into south-western Algeria [5], evidence of human exposure in Tunisia [6], mention of viral presence in Morocco, Algeria and Libya [7] and mention of exposure of camels, gazelle and water buffalo in Turkey [8]. Currently, an epidemic is being experienced in East Africa (Kenya, Rwanda, Tanzania and Uganda reporting cases in humans and animals, ProMED-mail, several postings http://www.promedmail.org). RVFv has been imported into countries outside the normal range, the most recent report being that of a patient, being diagnosed in China and having acquired the infection in Angola [9].

The epidemics in eastern Africa and the Horn of Africa involve a 5–15 year cycle marked by abnormally high rainfall, *e.g*. during the warm phase of the El Niño/Southern Oscillation phenomenon (ENSO) [10, 11]. In other regions of Africa, the occurrence of the disease is linked to other sources of flooding, *e.g*. the construction of a hydroelectric dam along the Senegal river [12, 13].

In the past, the above was the traditional view of the epidemiology of RVF, but recently there is more and more evidence of so-called inter-epidemic transmission: previously unnoticed low-level viral transmission in all species involved [12, 14–18]. In Tanzania, human involvement in RVF inter-epidemic transmission has been reported in the past [19, 20]. During the 2006/07 RVF epidemic in eastern Africa, livestock and people in the Kilombero valley in Tanzania were affected [21]. Two serological surveys in this region since this last epidemic, one in livestock and one in people, effectively showed the presence of inter-epidemic transmission in the area [17, 22].

RVF is transmitted to humans and other mammalian hosts, both livestock and wild ruminants (*e.g*. cattle, buffalo, sheep, goats and camels) through mosquito (*e.g*. *Culex* spp., *Ædes* spp. and *Mansonia* spp.) and other arthropod vector bites [1, 2, 16, 23]. Ædine mosquitoes are capable of transovarial (= vertical) transmission of RVFv to the eggs, which can survive long droughts (several years) and hatch when new water arrives during *e.g*. the ENSO phenomenon, resulting in infected larvæ and adult mosquitoes [2]. The highest risk for humans to become infected is through direct and indirect contact with infectious animal materials (blood, body fluids or tissues of viræmic animals). Ærosol formation during *e.g*. milking or consumption of raw milk, meat or blood form another risk for transmission [13, 24–28]. An established treatment method or a vaccine for humans currently does not exist. Control of the disease needs to be done through vaccination of livestock and preventive measures by humans [29, 30].

Clinical manifestation in humans can go from only mild illness, including fever, muscle pain, joint pain and headache to severe forms with ocular disease, meningo-encephalitis or haemorrhagic fever [29, 31]. The disease manifests itself in livestock through morbidity and mortality in newborns and abortions during all stages of the pregnancy. This has devastating effects on livestock populations and has severe economic repercussions for livestock keepers [2, 26, 32, 33].

Quantitative analysis and simulation modelling of RVFv dynamics have been undertaken on several occasions. Note that the list that follows cites only typical examples and that many more publications exist dealing with RVF modelling. The analytical models use environmental characteristics and range from post-hoc predictions of where outbreaks were to be expected during the 2006-2007 epidemic in East Africa [10] over statistical modelling in order to identify landscape features related to RVFv transmission [34] to the identification of ranges of potential vectors [35]. Simulation models include temporal models using differential equations [36] with extensions to spatial components [37]. Risk analysis of introduction into new territory (*in casu* The Netherlands) [38] has also been carried out. An overview of compartmental models, applied to the simulation of RVF dynamics, is provided by Danzetta and colleagues [39].

The existing models all suffer from being closed, inaccessible and specialised. The combination of R/RStudio^®^ with the libraries shiny and deSolve offers the possibility to develop open-source, easily applicable, accessible and modifiable models that can, on the one hand, be adapted to a specific situation with minimal programming effort and, on the other hand, be perused by the epidemiological researcher to study different scenarios and/or the effects of different parameter settings. The model presented here has been developed for the specific situation in East Africa, but as explained above, it can easily be adapted to other areas/situations, mostly by switching on or off certain parameters or parameter groups or by the inclusion of extensions with minimal new coding. The model presented in this paper is thus to be considered a research tool, allowing the user to study the effect(s) of different scenarios in order to better understand RVFv transmission dynamics and the mammalian hosts and arthropod vectors involved, and ultimately to assist in the formulation of new research questions. The model is not a predictive tool, as too much uncertainty still exists with regards to the actual dynamics of inter-epidemic transmission of the virus.

## Model—General description

**Fig 2:**
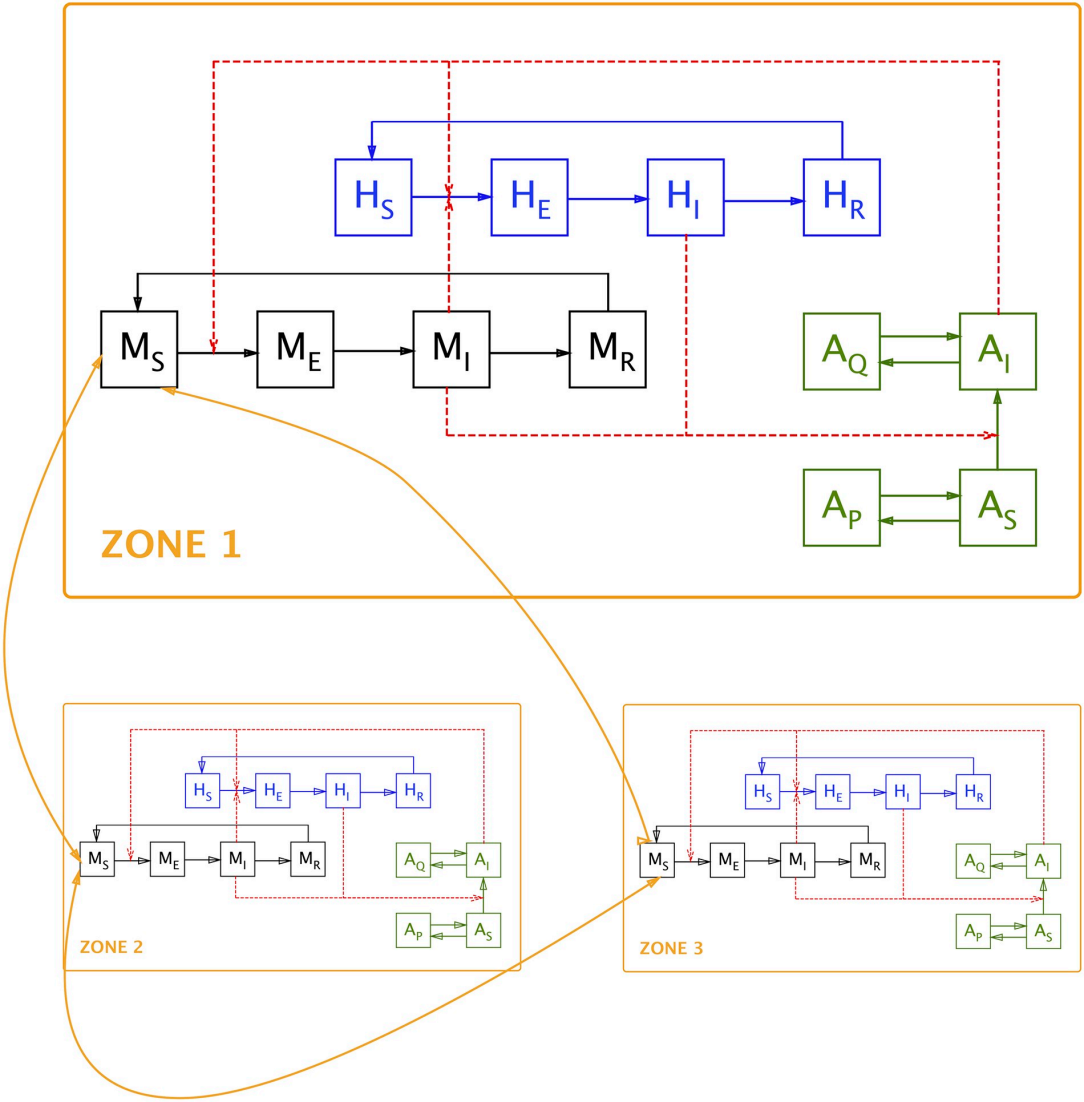
Diagrammatic representation of the model. Note: for the sake of clarity, inter-zone movement is indicated only for the susceptible animal compartment (*M*_*S*_); it is identical for all other compartments. Also for the sake of clarity, compartments are only shown for human population (*H*), domestic animal population (*M*) and one vector species (*A*); see Table 1 for a list of all compartments.

The model describes the RVFv transmission dynamics in six species (human population, domestic animal population and four vectors) in three different areas. The model attempts to offer maximal flexibility, whilst remaining manageable. The model allows for migration of the various species between the different areas. The different compartments in the model are presented in Table 1 and a simplified schematic representation of the model is shown in Fig 2.

**Table 1:** Different compartments in the model.

Zone 1				Zone 2				Zone 3			
$H_{S}^{1}$	$H_{E}^{1}$	$H_{I}^{1}$	$H_{R}^{1}$	$H_{S}^{2}$	$H_{E}^{2}$	$H_{I}^{2}$	$H_{R}^{2}$	$H_{S}^{3}$	$H_{E}^{3}$	$H_{I}^{3}$	$H_{R}^{3}$
$M_{S}^{1}$	$M_{E}^{1}$	$M_{I}^{1}$	$M_{R}^{1}$	$M_{S}^{2}$	$M_{E}^{2}$	$M_{I}^{2}$	$M_{R}^{2}$	$M_{S}^{3}$	$M_{E}^{3}$	$M_{I}^{3}$	$M_{R}^{3}$
$A_{Q}^{1}$	$A_{P}^{1}$	$A_{S}^{1}$	$A_{I}^{1}$	$A_{Q}^{2}$	$A_{P}^{2}$	$A_{S}^{2}$	$A_{I}^{2}$	$A_{Q}^{3}$	$A_{P}^{3}$	$A_{S}^{3}$	$A_{I}^{3}$
$B_{Q}^{1}$	$B_{P}^{1}$	$B_{S}^{1}$	$B_{I}^{1}$	$B_{Q}^{2}$	$B_{P}^{2}$	$B_{S}^{2}$	$B_{I}^{2}$	$B_{Q}^{3}$	$B_{P}^{3}$	$B_{S}^{3}$	$B_{I}^{3}$
	$C_{P}^{1}$	$C_{S}^{1}$	$C_{I}^{1}$		$C_{P}^{2}$	$C_{S}^{2}$	$C_{I}^{2}$		$C_{P}^{3}$	$C_{S}^{3}$	$C_{I}^{3}$
	$D_{P}^{1}$	$D_{S}^{1}$	$D_{I}^{1}$		$D_{P}^{2}$	$D_{S}^{2}$	$D_{I}^{2}$		$D_{P}^{3}$	$D_{S}^{3}$	$D_{I}^{3}$

*H* = People; *M* = Domestic animals; *A* = Vector A; *B* = Vector B; *C* = Vector C; *D* = Vector D;

□_*S*_ = susceptible; □_*E*_ = exposed; □_*I*_ = infective; □_*R*_ = removed;

□_*Q*_ = infected eggs; □_*P*_ = non-infected eggs;

□^1^ = Zone 1; □^2^ = Zone 2; □^3^ = Zone 3

Each human and animal population consists of a susceptible *S*, exposed *E*, infective *I* and removed *R* (= recovered/immune) compartment. There is a flow back from the removed to the susceptible compartment in both populations, *i.e*. immunity is not lifelong. All individuals are born susceptible and a proportion of the pregnant infected animals abort. Vectors *A* and *B* allow for vertical transmission: infected females (*I* compartment) transmit infection to their eggs (*Q* compartment), where the virus survives until the larvæ hatch and the resulting adults are infective. Vector *A* furthermore has the possibility of long-term dormancy in the egg stage (both infected and non-infected).

A challenge lies in the correct modelling of the vector dynamics. More specifically, a point of attention is the distribution of feeding individuals over the different host populations (both species-wise and zone-wise). Vectors can feed on the two modelled host species (human and domestic animal), but they can also use alternative hosts (especially so in the forest zone). The latter means there is no increased mortality in case the two main hosts are not available, but this of course also influences infection prevalence in the vector population. The vector populations are furthermore limited by a density-dependent oviposition rate. The approach currently taken uses the following basic parameters (see **Vector feeding and infection rates** for details):

- *ε*: proportion of vector Ξ feeding on host Λ in zone *i*; it is the user’s responsibility to ensure that the sum of the various *ε* per species per zone does not exceed one
- *η*: (maximum) number of successful bites per time unit of vector Ξ on host Λ
- *π*_*uv*_: probability to transmit infection from species *u* to species *v* (*v* ≠ *u*) upon a successful bite
- Ω_*alt*_: number of alternative hosts
- $\kappa_{\Xi }^{j}$: maximum number of vector Ξ individuals in zone *j* (‘carrying capacity’)

El Niño events are currently modelled to occur every ten years. Additionally, the user is given the opportunity to include annual overall climate variability through the choice of a random series of ‘dry’ or ‘wet’ years and a seasonal within-year variation in egg eclosion to model seasonal effects on vector population size. Finally, there is the possibility of including a ‘fixed’ annual domestic animal movements between zones 1 and 2, simulating seasonal transhumance of (*e.g*.) cattle between the plateau and the floodplain. Details are to be found in **Seasonality and El Niño effect**.

It is understood that the necessary calculations for these density-dependent oviposition-feeding, climatic variability and transhumance processes slow down the model considerably. It was therefore decided to rewrite part of the code, doing the preparatory computations before calling the deSolve routines (using the classical Runge-Kutta 4^th^ order method), in C++ (making use of the RCCP library). This speeds up execution by a factor of about sixty, but of course means a lower accessibility of the code. Therefore, a slower version, entirely written in R is also offered. Full details on how to install and run the model are given in the accompanying user’s manual S1 Appendix. The R and C++ code is provided in S2 Appendix.

## Model—Differential equations

For every zone *i*(*i* = 1, 2, 3), we compute the differential equations of each compartment of the human, the animal and the vector populations.

### Human population

$$\frac{dH_{S}^{i}}{dt}=\gamma_{H}N_{H}^{i}+\sum_{\begin{array}{c} j=1\\ j\neq i \end{array}}^{3}\lambda_{H}^{ji}H_{S}^{j}+\rho_{H}H_{R}^{i}-\left(\mu_{H}+\beta_{H}^{i}+\sum_{\begin{array}{c} j=1\\ j\neq i \end{array}}^{3}\lambda_{H}^{ij}\right)H_{S}^{i};\left(i=1,\ldots ,3\right)$$ (1)

$$\frac{dH_{E}^{i}}{dt}=\beta_{H}^{i}H_{S}^{i}+\sum_{\begin{array}{c} j=1\\ j\neq i \end{array}}^{3}\lambda_{H}^{ji}H_{E}^{j}-\left(\mu_{H}+\xi_{H}+\sum_{\begin{array}{c} j=1\\ j\neq i \end{array}}^{3}\lambda_{H}^{ij}\right)H_{E}^{i};\left(i=1,\ldots ,3\right)$$ (2)

$$\frac{dH_{I}^{i}}{dt}=\xi_{H}H_{E}^{i}+\sum_{\begin{array}{c} j=1\\ j\neq i \end{array}}^{3}\lambda_{H}^{ji}H_{I}^{j}-\left(\mu_{H}+\delta_{H}+\alpha_{H}+\sum_{\begin{array}{c} j=1\\ j\neq i \end{array}}^{3}\lambda_{H}^{ij}\right)H_{I}^{i};\left(i=1,\ldots ,3\right)$$ (3)

$$\frac{dH_{R}^{i}}{dt}=\alpha_{H}H_{I}^{i}+\sum_{\begin{array}{c} j=1\\ j\neq i \end{array}}^{3}\lambda_{H}^{ji}H_{R}^{2}-\left(\mu_{H}+\rho_{H}+\sum_{\begin{array}{c} j=1\\ j\neq i \end{array}}^{3}\lambda_{H}^{ij}\right)H_{R}^{i};\left(i=1,\ldots ,3\right)$$ (4)

Eq 1 describes the rate of change in the susceptible human compartment in Zone *i*: $\gamma_{H}N_{H}^{i}$ refers to the newborn individuals, $\sum_{\begin{array}{c} j=1\\ j\neq i \end{array}}^{3}\lambda_{H}^{ji}H_{S}^{j}+\rho_{H}H_{R}^{i}$ refers to the immigration into Zone *i* from the other two zones and individuals losing their immunity while $\left(\mu_{H}+\beta_{H}^{i}+\sum_{\begin{array}{c} j=1\\ j\neq i \end{array}}^{3}\lambda_{H}^{ij}\right)H_{S}^{i}$ refers to the losses through natural mortality, people becoming infected and emigration out of Zone *i*. Eq 2 describes the rate of change in the human exposed (incubating) compartment in Zone *i*: $\beta_{H}^{i}H_{S}^{i}$ refers to the individuals having become infected, $\sum_{\begin{array}{c} j=1\\ j\neq i \end{array}}^{3}\lambda_{H}^{ji}H_{E}^{j}$ refers to immigration into zone *i* and $\mu_{H}+\xi_{H}+\sum_{\begin{array}{c} j=1\\ j\neq i \end{array}}^{3}\lambda_{H}^{ij})H_{E}^{i}$ refers to the losses through natural mortality, changing from incubation to the infective stage and emigration from Zone *i*. Eq 3 describes the rate of change in the infective human compartment: $\xi_{H}H_{E}^{i}$ refers to the individuals having become infective, $\sum_{\begin{array}{c} j=1\\ j\neq i \end{array}}^{3}\lambda_{H}^{ji}H_{I}^{j}$ refers to the immigration into Zone *i* and $\left(\mu_{H}+\delta_{H}+\alpha_{H}+\sum_{\begin{array}{c} j=1\\ j\neq i \end{array}}^{3}\lambda_{H}^{ij}\right)H_{I}^{i}$ refers to the losses through natural mortality, disease-specific mortality, recovery and emigration from Zone *i*. Eq 4 describes the rate of change in the recovered (immune) human compartment: $\alpha_{H}H_{I}^{i}$ refers to individuals having recovered (gained immunity), $\sum_{\begin{array}{c} j=1\\ j\neq i \end{array}}^{3}\lambda_{H}^{ji}H_{R}^{2}$ refers to immigration into Zone *i* and $\left(\mu_{H}+\rho_{H}+\sum_{\begin{array}{c} j=1\\ j\neq i \end{array}}^{3}\lambda_{H}^{ij}\right)H_{R}^{i}$ refers to losses through natural mortality, loss of immunity and emigration from Zone *i*.

### Animal population

$$\frac{dM_{S}^{i}}{dt}=(\gamma_{M_{U}}N_{M}^{i}+\gamma_{M_{I}}M_{I}^{i})(1-\frac{N_{M}^{i}}{\kappa_{M}^{i}})+\sum_{\begin{array}{c} j=1\\ j\neq i \end{array}}^{3}\lambda_{M}^{ji}M_{S}^{j}+\rho_{M}M_{R}^{i}-\left(\mu_{M}+\beta_{M}^{i}+\sum_{\begin{array}{c} j=1\\ j\neq i \end{array}}^{3}\lambda_{M}^{ij}\right)M_{S}^{i};\left(i=1,\ldots ,3\right)$$ (5)

$$\frac{dM_{E}^{i}}{dt}=\beta_{M}^{i}M_{S}^{i}+\sum_{\begin{array}{c} j=1\\ j\neq i \end{array}}^{3}\lambda_{M}^{ji}M_{E}^{j}-\left(\mu_{M}+\xi_{M}+\sum_{\begin{array}{c} j=1\\ j\neq i \end{array}}^{3}\lambda_{M}^{ij}\right)M_{E}^{i};\left(i=1,\ldots ,3\right)$$ (6)

$$\frac{dM_{I}^{i}}{dt}=\xi_{M}M_{E}^{i}+\sum_{\begin{array}{c} j=1\\ j\neq i \end{array}}^{3}\lambda_{M}^{ji}M_{I}^{j}-\left(\mu_{M}+\delta_{M}+\alpha_{M}+\sum_{\begin{array}{c} j=1\\ j\neq i \end{array}}^{3}\lambda_{M}^{ij}\right)M_{I}^{i};\left(i=1,\ldots ,3\right)$$ (7)

$$\frac{dM_{R}^{i}}{dt}=\alpha_{M}M_{I}^{i}+\sum_{\begin{array}{c} j=1\\ j\neq i \end{array}}^{3}\lambda_{M}^{ji}M_{R}^{j}-\left(\mu_{M}+\rho_{M}+\sum_{\begin{array}{c} j=1\\ j\neq i \end{array}}^{3}\lambda_{M}^{ij}\right)M_{R}^{i};\left(i=1,\ldots ,3\right)$$ (8)

Eq 5 describes the rate of change in the susceptible animal host compartment: $\left(\gamma_{M_{U}}N_{M}^{i}+\gamma_{M_{I}}M_{I}^{i})(1-\frac{N_{M}^{i}}{\kappa_{M}^{i}}\right)$ refers to the newborn individuals, respectively born from uninfected and infected individuals and corrected for population density to simulate removal (sales) in function of herd size, $\sum_{\begin{array}{c} j=1\\ j\neq i \end{array}}^{3}\lambda_{M}^{ji}M_{S}^{j}+\rho_{M}M_{R}^{i}$ refers to immigration into Zone *i* from the other two zones and individuals losing their immunity and $\left(\mu_{M}+\beta_{M}^{i}+\sum_{\begin{array}{c} j=1\\ j\neq i \end{array}}^{3}\lambda_{M}^{ij}\right)M_{S}^{i}$ refers to losses through natural mortality, animals becoming infected and emigration out of Zone *i*. Eq 6 describes the rate of change in the animal host exposed (incubating) compartment in Zone *i*: $\beta_{M}^{i}M_{S}^{i}$ refers to the animals becoming infected, $\sum_{\begin{array}{c} j=1\\ j\neq i \end{array}}^{3}\lambda_{M}^{ji}M_{E}^{j}$ refers to immigration into Zone *i* and $\left(\mu_{M}+\xi_{M}+\sum_{\begin{array}{c} j=1\\ j\neq i \end{array}}^{3}\lambda_{M}^{ij}\right)M_{E}^{i}$ refers to the losses through natural mortality, changing from incubation to the infective stage and emigration from Zone *i*. Eq 7 describes the rate of change in the animal infective compartment in Zone *i*: $\xi_{M}M_{E}^{i}$ refers to the individuals becoming infective, $\sum_{\begin{array}{c} j=1\\ j\neq i \end{array}}^{3}\lambda_{M}^{ji}M_{I}^{j}$ refers to the immigration into Zone *i* ands $\left(\mu_{M}+\delta_{M}+\alpha_{M}+\sum_{\begin{array}{c} j=1\\ j\neq i \end{array}}^{3}\lambda_{M}^{ij}\right)M_{I}^{i}$ refers to the losses through natural mortality, disease-specific mortality, recovery and emigration from Zone *i*. Eq 8 describes the rate of change in the recovered (immune) animal compartment in Zone *i*: $\alpha_{M}M_{I}^{i}$ refers to the animals having recovered (gained immunity), $\sum_{\begin{array}{c} j=1\\ j\neq i \end{array}}^{3}\lambda_{M}^{ji}M_{R}^{j}$ refers to immigration into Zone *i* and $\left(\mu_{M}+\rho_{M}+\sum_{\begin{array}{c} j=1\\ j\neq i \end{array}}^{3}\lambda_{M}^{ij}\right)M_{R}^{i}$ refers to losses through natural mortality, loss of immunity and emigration from Zone *i*.

### Vector A

$$\frac{dA_{Q}^{i}}{dt}=\omega_{A}^{i}\gamma_{A}(1-\frac{N_{A}^{i}}{\kappa_{A}^{i}})\zeta_{A}A_{I}^{i}-\left(\mu_{A_{Q}}^{i}+\tau_{s}\tau_{A}^{i}\right)A_{Q}^{i};\left(i=1,\ldots ,3\right)$$ (9)

$$\frac{dA_{P}^{i}}{dt}=\gamma_{A}(1-\frac{N_{A}^{i}}{\kappa_{A}^{i}})[\omega_{A}^{i}\left(1-\zeta_{A}\right)A_{I}^{i}+\left(\omega_{A}^{i}+\omega_{A_{2}}^{1}\right)A_{S}^{i}]-(\mu_{A_{P}}^{i}+\tau_{s}\tau_{A}^{i})A_{P}^{i};\left(i=1,\ldots ,3\right)$$ (10)

$$\frac{dA_{S}^{i}}{dt}=\tau_{s}\tau_{A}^{i}A_{P}^{i}+\sum_{\begin{array}{c} j=1\\ j\neq i \end{array}}^{3}\lambda_{A}^{ji}A_{S}^{j}-\left(\mu_{A}+\omega_{A}^{i}\beta_{A}^{i}+\sum_{\begin{array}{c} j=1\\ j\neq i \end{array}}^{3}\lambda_{A}^{ij}\right)A_{S}^{i};\left(i=1,\ldots ,3\right)$$ (11)

$$\frac{dA_{I}^{i}}{dt}=\tau_{s}\tau_{A}^{i}A_{Q}^{i}+\omega_{A}^{i}\beta_{A}^{i}A_{S}^{i}+\sum_{\begin{array}{c} j=1\\ j\neq i \end{array}}^{3}\lambda_{A}^{ji}A_{I}^{j}-\left(\mu_{A}+\sum_{\begin{array}{c} j=1\\ j\neq i \end{array}}^{3}\lambda_{A}^{ij}\right)A_{I}^{i};\left(i=1,\ldots ,3\right)$$ (12)

Eq 9 describes the rate of change in the infected-egg compartment of Vector A in Zone *i*: $\omega_{A}^{i}\gamma_{A}(1-\frac{N_{A}^{i}}{\kappa_{A}^{i}})\zeta_{A}A_{I}^{i}$ refers to the production of infected eggs (product of total biting rate, egg production rate, density-dependent correction and vertical transmission rate) while $\left(\mu_{A_{Q}}^{i}+\tau_{s}\tau_{A}^{i}\right)A_{Q}^{i}$ refers to losses through mortality and hatching (in function of El Niño and seasonal flooding through *τ*_*s*_). Eq 10 describes the rate of change in the uninfected-egg compartment of Vector A in Zone *i*: $\gamma_{A}(1-\frac{N_{A}^{i}}{\kappa_{A}^{i}})[\omega_{A}^{i}\left(1-\zeta_{A}\right)A_{I}^{i}+\left(\omega_{A}^{i}+\omega_{A_{2}}^{1}\right)A_{S}^{i}]$ refers to the density-dependence corrected production of uninfected eggs both by infected adult vectors (absence of vertical transmission) and uninfected adult vectors while $(\mu_{A_{P}}^{i}+\tau_{s}\tau_{A}^{i})A_{P}^{i}$ refers to losses through mortality and hatching (in function of El Niño and seasonal flooding through *τ*_*s*_). Eq 11 describes the rate of change in the uninfected-adult-vector compartment in Zone *i*: $\tau_{s}\tau_{A}^{i}A_{P}^{i}$ refers to the newly ‘hatched’ adults (note that stages intervening between egg and adult are omitted, requiring adjustment of hatching and mortality rates), $\sum_{\begin{array}{c} j=1\\ j\neq i \end{array}}^{3}\lambda_{A}^{ji}A_{S}^{j}$ refers to the immigration into Zone *i* and $\left(\mu_{A}+\omega_{A}^{i}\beta_{A}^{i}+\sum_{\begin{array}{c} j=1\\ j\neq i \end{array}}^{3}\lambda_{A}^{ij}\right)A_{S}^{i}$ refers to the losses through mortality, acquisition of infection and emigration out of Zone *i*. Eq 12 describes the rate of change in the infected-adult-vector compartment in Zone *i*: $\tau_{s}\tau_{A}^{i}A_{Q}^{i}$ refers to the newly ‘hatched’ infected adult vectors (same remark as for Eq 11), $\omega_{A}^{i}\beta_{A}^{i}A_{S}^{i}$ refers to newly infected adult vectors, $\sum_{\begin{array}{c} j=1\\ j\neq i \end{array}}^{3}\lambda_{A}^{ji}A_{I}^{j}$ refers to the immigration into Zone *i* and $\left(\mu_{A}+\sum_{\begin{array}{c} j=1\\ j\neq i \end{array}}^{3}\lambda_{A}^{ij}\right)A_{I}^{i}$ refers to the losses through mortality and emigration out of Zone *i*.

### Vector B

$$\frac{dB_{Q}^{i}}{dt}=\omega_{B}^{i}\gamma_{B}(1-\frac{N_{B}^{i}}{\kappa_{B}^{i}})\zeta_{B}B_{I}^{i}-\left(\mu_{B_{Q}}^{i}+\tau_{s}\tau_{B}^{i}\right)B_{Q}^{i};\left(i=1,\ldots ,3\right)$$ (13)

$$\frac{dB_{P}^{i}}{dt}=\gamma_{B}(1-\frac{N_{B}^{i}}{\kappa_{B}^{i}})[\omega_{B}^{i}\left(1-\zeta_{B}\right)B_{I}^{i}+\left(\omega_{B}^{i}+\omega_{B_{2}}^{i}\right)B_{S}^{i}]-(\mu_{B_{P}}^{i}+\tau_{s}\tau_{B}^{i})B_{P}^{i};\left(i=1,\ldots ,3\right)$$ (14)

$$\frac{dB_{S}^{i}}{dt}=\tau_{s}\tau_{B}^{i}B_{P}^{i}+\sum_{\begin{array}{c} j=1\\ j\neq i \end{array}}^{3}\lambda_{B}^{ji}B_{S}^{j}-\left(\mu_{B}+\omega_{B}^{i}\beta_{B}^{i}+\sum_{\begin{array}{c} j=1\\ j\neq i \end{array}}^{3}\lambda_{B}^{ij}\right)B_{S}^{i};\left(i=1,\ldots ,3\right)$$ (15)

$$\frac{dB_{I}^{i}}{dt}=\tau_{s}\tau_{B}^{i}B_{Q}^{i}+\omega_{B}^{i}\beta_{B}^{i}B_{S}^{i}+\sum_{\begin{array}{c} j=1\\ j\neq i \end{array}}^{3}\lambda_{B}^{ji}B_{I}^{j}-\left(\mu_{B}+\sum_{\begin{array}{c} j=1\\ j\neq i \end{array}}^{3}\lambda_{B}^{ij}\right)B_{I}^{i};\left(i=1,\ldots ,3\right)$$ (16)

The differential equations describing the dynamics of Vector B are identical as those for Vector A, the only difference being the possible presence of dormant eggs in the latter and not in the former.

### Vector C

$$\frac{dC_{P}^{i}}{dt}=\gamma_{C}(1-\frac{N_{C}^{i}}{\kappa_{C}^{i}})[\omega_{C}^{i}C_{I}^{i}+(\omega_{C}^{i}+\omega_{C_{2}}^{i})C_{S}^{i}]-(\mu_{C_{P}}^{i}+\tau_{s}\tau_{C})C_{P}^{i};\left(i=1,\ldots ,3\right)$$ (17)

$$\frac{dC_{S}^{i}}{dt}=\tau_{s}\tau_{C}C_{P}^{i}+\sum_{\begin{array}{c} j=1\\ j\neq i \end{array}}^{3}\lambda_{C}^{ji}C_{S}^{j}-\left(\mu_{C}+\omega_{C}^{i}\beta_{C}^{i}+\sum_{\begin{array}{c} j=1\\ j\neq i \end{array}}^{3}\lambda_{C}^{ij}\right)C_{S}^{i};\left(i=1,\ldots ,3\right)$$ (18)

$$\frac{dC_{I}^{i}}{dt}=\omega_{C}^{i}\beta_{C}^{i}C_{S}^{i}+\sum_{\begin{array}{c} j=1\\ j\neq i \end{array}}^{3}\lambda_{C}^{ji}C_{I}^{j}-\left(\mu_{C}+\sum_{\begin{array}{c} j=1\\ j\neq i \end{array}}^{3}\lambda_{C}^{ij}\right)C_{I}^{i};\left(i=1,\ldots ,3\right)$$ (19)

Vector C differs from Vectors A and B in the absence of vertical transmission and hence the absence of an infected-egg compartment (*i.e*. no $\frac{dC_{Q}^{i}}{dt}$ differential equation). Infected adult vectors can only originate through uninfected adults acquiring infection ($\omega_{C}^{i}\beta_{C}^{i}C_{S}^{i}$) and there is therefore no ‘hatching’ term in the equation (*i.e*. no $\tau_{s}\tau_{C}^{i}C_{Q}^{i}$ term).

### Vector D

$$\frac{dD_{P}^{i}}{dt}=\gamma_{D}(1-\frac{N_{D}^{i}}{\kappa_{D}^{i}})[\omega_{D}^{i}D_{I}^{i}+(\omega_{D}^{i}+\omega_{D_{2}}^{i})D_{S}^{i}]-(\mu_{D_{P}}^{i}+\tau_{s}\tau_{D})D_{P}^{i};\left(i=1,\ldots ,3\right)$$ (20)

$$\frac{dD_{S}^{i}}{dt}=\tau_{s}\tau_{D}D_{P}^{i}+\sum_{\begin{array}{c} j=1\\ j\neq i \end{array}}^{3}\lambda_{D}^{ji}D_{S}^{j}-\left(\mu_{D}+\omega_{D}^{i}\beta_{D}^{i}+\sum_{\begin{array}{c} j=1\\ j\neq i \end{array}}^{3}\lambda_{D}^{ij}\right)D_{S}^{i};\left(i=1,\ldots ,3\right)$$ (21)

$$\frac{dD_{I}^{i}}{dt}=\omega_{D}^{i}\beta_{D}^{i}D_{S}^{i}+\sum_{\begin{array}{c} j=1\\ j\neq i \end{array}}^{3}\lambda_{D}^{ji}D_{I}^{j}-\left(\mu_{D}+\sum_{\begin{array}{c} j=1\\ j\neq i \end{array}}^{3}\lambda_{D}^{ij}\right)D_{I}^{i};\left(i=1,\ldots ,3\right)$$ (22)

Vector D is identical to Vector C.

## Auxiliary equations

### Population totals

$$N_{H}^{i}=H_{S}^{i}+H_{E}^{i}+H_{I}^{i}+H_{R}^{i};\left(i=1,\ldots ,3\right)$$ (23)

$$N_{M}^{i}=M_{S}^{i}+M_{E}^{i}+M_{R}^{i};\left(i=1,\ldots ,3\right)$$ (24)

$$N_{A}^{i}=A_{Q}^{i}+A_{P}^{i}+A_{S}^{i}+A_{I}^{i};\left(i=1,\ldots ,3\right)$$ (25)

$$N_{B}^{i}=B_{Q}^{i}+B_{P}^{i}+B_{S}^{i}+B_{I}^{i};\left(i=1,\ldots ,3\right)$$ (26)

$$N_{C}^{i}=C_{P}^{i}+C_{S}^{i}+C_{I}^{i};\left(i=1,\ldots ,3\right)$$ (27)

$$N_{D}^{i}=D_{P}^{i}+D_{S}^{i}+D_{I}^{;}\left(i=1,\ldots ,3\right)i$$ (28)

### Vector feeding and infection rates

Parameters 29–35 are the basic parameters used to compute carrying capacity etc. of a zone *vis-à-vis* its resident vectors. The present approach is to compare the total number of bites (successful feedings, …– for sake of brevity referred to as ‘bites’ from now on) the vectors can inflict upon the hosts per time unit with the total number of number of vector bites the host populations can sustain (given their resistance, evasive behaviour, …). The minimum value of these two is used to compute the actual number of bites given per vector and/or the number of bites suffered per host. It is understood that this approach may introduce a number of parameters whose values are only vaguely known at best, but an attempt was made to avoid unrealistic numbers of vectors interacting with a single host, *i.e*. host numbers determine vector numbers. At the same time, the possibility is offered to include so-called alternative hosts, which can be used by the vectors when the hosts included in the model are insufficient, in order to avoid vectors disappearing when host population levels are too low.

$$\varepsilon_{kj}=\text{proportion}\;\text{of}\;\text{vector}\;\text{population}\;\Xi_{k}\;\text{feeding}\;\text{on}\;\text{host}\;\Lambda_{j}\;\sum_{j}\varepsilon_{kj}\leq 1)$$ (29)

$$\nu_{k}=\text{average}\;\text{number}\;\text{of}\;\text{bites}\;\text{an}\;\text{individual}\;\text{of}\;\text{vector}\;\Xi_{k}\;\text{issues}\;\text{per}\;\text{time}\;\text{unit}$$ (30)

$$\begin{array}{cc} \eta_{j} & =\text{maximum}\;\text{number}\;\text{of}\;\text{bites}\;\text{host}\;\Lambda_{j}\;\text{can}\;‘\text{sustain}’\;\text{per}\;\text{time}\;\text{unit,}\\ & \;\;\text{before}\;e.g.\;\text{taking}\;\text{evasive}\;\text{action}\;\text{or}\;\text{dislodging}\;\text{behaviour} \end{array}$$ (31)

$$\varphi_{j^{\prime },j}=\text{number}\;\text{of}\;j^{\prime }\;\text{transmitting}\;\text{hosts}\;\text{contacted}\;\text{by}\;\text{receiving}\;\text{host}\;j\;\text{per}\;\text{time}\;\text{unit}$$ (32)

$$\pi_{uv}=\text{probability}\;\text{to}\;\text{transmit}\;\text{infection}\;\text{from}\;u\;\text{to}\;v$$ (33)

withu∈{j,k}&v∈{k,j}&v≠u (34)

$$\beta_{\text{wl}}=\text{probability}\;\text{to}\;\text{pick}\;\text{up}\;\text{infection}\;\text{from}\;\text{wildlife}\;\text{hosts}\;\text{in}\;\text{general}$$ (35)

Parameters 36 and 37 are computed from the simulation output:

$$N_{\Xi_{k}}=\text{Population}\;\text{size}\;\text{of}\;\text{vector}\;\Xi_{k}$$ (36)

$$N_{\Lambda_{j}}=\text{Population}\;\text{size}\;\text{of}\;\text{host}\;\Lambda_{j}$$ (37)

The potential maximum number of vector bites (all vector species) on whole host population Λ_*j*_ is computed as:

$$\Omega_{j}=\sum_{k}\varepsilon_{kj}N_{\Xi_{k}}\nu_{k}$$ (38)

This is compared with the maximum number of bites the same host population can ‘sustain’ (see above for more details):

$$\chi_{j}=\eta_{j}N_{\Lambda_{j}}$$ (39)

The ‘availability’ of host population Λ_*j*_ (*i.e*. the proportion of the potential bites actual inflicted on the host population in question) is the ratio of parameter 39 over parameter 38 with a maximum of unity:

$$\sigma_{j}=\text{min}(1,\frac{\chi_{j}}{\Omega_{j}})$$ (40)

The actual number of bites by vector Ξ_*k*_ on the whole host population Λ_*j*_ is thus:

$$\Omega_{kj}=\varepsilon_{kj}N_{\Xi_{k}}\nu_{k}\sigma_{j}$$ (41)

The individual biting rate of vector Ξ_*k*_ on host Λ_*j*_ per time unit becomes:

$$\omega_{kj}=\varepsilon_{kj}\nu_{k}\sigma_{j}$$ (42)

The total individual biting rate of vector Ξ_*k*_ on all host populations per time unit therefore is the sum of the respective *ω*_*kj*_:

$$\omega_{k}=\sum_{j}\omega_{kj}$$ (43)

The biting rate of vector Ξ_*k*_ on alternative hosts (with Ω_*alt*_ = number of alternative hosts) is defined as:

$$\omega_{k_{2}}=\frac{\Omega_{\text{alt}}}{N_{\Xi_{k}}}$$ (44)

The **proportion** of infection in vector Ξ_*k*_ feeding on all modelled hosts species is computed as (the reference to the zone is left out, $I_{\Lambda_{j}}$ being the number of infective individuals of host Λ_*j*_; *β*_wl_ refers to the infection picked up from game animals and it is added only in the case of Zone-3-dwelling vectors):

$$\beta_{k}=\text{min}(1,\sum_{j}\pi_{jk}\frac{I_{\Lambda_{j}}}{N_{\Lambda_{j}}}+\beta_{\text{wl}})$$ (45)

The infection **rate** of host Λ_*j*_ being subjected to the actual number of bites by the various vectors and/or interacting with other infectious hosts is calculated as (*φ*_*j*′, *j*_ refers to the number of transmitting hosts [domestic animal] met by one receiving host [a person] per time unit; $\frac{\Omega_{kj}}{N_{\Lambda_{j}}}\frac{I_{\Xi_{k}}}{N_{\Xi_{k}}}$ becomes $\frac{\omega_{kj}I_{\Xi_{k}}}{N_{\Lambda_{j}}}$ because $\omega_{kj}=\frac{\Omega_{kj}}{N_{\Xi_{k}}}$):

$$\begin{array}{c} \beta_{j}=-\text{log}\{1-[1-\prod_{k}(1-\pi_{\Xi_{k}\Lambda_{j}}{)}^{\frac{\omega_{kj}I_{\Xi_{k}}}{N_{\Lambda_{j}}}}]-[1-\prod_{j^{\prime }}(1-\pi_{\Lambda_{j^{\prime }}\Lambda_{j}}{)}^{\frac{\varphi_{j^{\prime },j}I_{\Lambda_{j^{\prime }}}}{N_{\Lambda_{j^{\prime }}}}}]\\ +[1-\prod_{k}(1-\pi_{\Xi_{k}\Lambda_{j}}{)}^{\frac{\omega_{kj}I_{\Xi_{k}}}{N_{\Lambda_{j}}}}]\;\times [1-\prod_{j^{\prime }}(1-\pi_{\Lambda_{j^{\prime }}\Lambda_{j}}{)}^{\frac{\varphi_{j^{\prime },j}I_{\Lambda_{j^{\prime }}}}{N_{\Lambda_{j^{\prime }}}}}]\}\;\forall j^{\prime }\neq j \end{array}$$ (46)

The second and third terms of the logarithm function of Eq 46 are currently implemented only for animal-to-human direct transmission.

### Seasonality and El Niño effect

Simulating an annual (seasonal) animal transhumance between Zone 1 and Zone 2 is possible: animals move to Zone 1 on day *d*_1_ and move back to Zone 2 on day *d*_2_. This is achieved through the generation of 0/1 indicators, which are to be multiplied with the movement rate:

$$\lambda_{M}^{12}=[t\equiv d_{1}\;\left(\text{mod}\;360\right)]$$ (47)

$$\lambda_{M}^{21}=[t\equiv d_{2}\;\left(\text{mod}\;360\right)]$$ (48)

Hatching of dormant eggs of Vector A can be regulated on a seasonal basis as well as periodically through El Niño events in Zone 1 (*d*_3_ and *d*_4_ are respectively the start and end of the annual flooding, *π*_*φ*_ is the proportion proportion of Zone 1 that is seasonally flooded; *d*_5_ and *d*_6_ are respectively the start and end of the El Niño event):

$$\tau_{A}^{1}=\underset{\text{seasonal}\;\text{flooding}}{\underset{︸}{\left[d_{3}\leq t\leq d_{4}\;\left(\text{mod}\;360\right)\right]*\pi_{\varphi }}}+\underset{\text{El}\;\text{Ni}ñ\text{o}\;\text{flooding}}{\underset{︸}{\left[d_{5}\leq t\leq d_{6}\;\left(\text{mod}\;3600\right)\right]}}$$ (49)

Annual variation (*e.g*. because of wet and dry years) and seasonal variation in vector egg eclosion (*τ*_*S*_) in all three zones can be included in the model: the current approach is by penalising hatching rates during dry years (hatching rate becomes a fraction –*π*_*δ*_– of normal rates) and by allowing hatching rates in normal and dry years to vary seasonally according to a cosine curve (see the accompanying user’s manual S1 Appendix for examples on different parameter settings). The different possible combinations are as follows in Table 2:

**Table 2:** Seasonal variation in vector egg eclosion.

Wet/dry year	Seasonal variation	*τ*_*S*_
wet	no	1
wet	yes	$\text{cos}[\frac{n\pi (t+\delta_{S})}{180}]$
dry	no	*π*_*δ*_
dry	yes	$\pi_{\delta }\text{cos}[\frac{n\pi (t+\delta_{S})}{180}]$

where: $\pi_{\delta }=\;\text{proportion}\frac{\text{hatching}\;\text{dry}\;\text{season}}{\text{hatching}\;\text{normal}\;\text{season}}$, *n* = number of optimums per annum, *δ*_*S*_ = shift from 1 January

## Model—Calibration

The model is calibrated using data that were extracted from two studies in the Kilombero Valley in Tanzania (Morogoro region, [17, 22]: the principal findings of these studies were the presence of inter-epidemic RVFv circulation in human and domestic animal populations and the location of so-called infection ‘hot-spots’ away from the floodplain and in fact closer to forested areas on the plateau. The Kilombero Valley region consists of a seasonally inundated floodplain between the densely forested escarpments of the Udzungwa mountains to the northwest and the grass covered Mahenge mountains to the southeast. The valley receives an average annual rainfall of 1200–1800 mm and the average monthly temperature ranges between 25℃ and 32℃. The valley has a diverse ecology and demography with villages consisting largely of numerous distinct groups of houses located on the margins of the floodplain where rice cultivation is the predominant economic activity. Other land use types include hunting, fishing, forestry, pastoral livestock rearing and cultivation of other crops. Several mosquito species inhabit the valley, including known vectors of RVFv, such as *Culex* spp., *Ædes* spp. and *Mansonia* spp. [17, 22, 40]. The zones, the two mammalian hosts and the four vector populations modelled are in this case:

- **Areas**
  - **Zone 1**: Floodplain (rice cultivation and dry season grazing)
  - **Zone 2**: Residential area (= village) & rainy season grazing area (= pastures)
  - **Zone 3**: Forest (people collect various resources, occasional grazing by cattle)
- **Species**
  - **H**: Human population
  - **M**: Cattle
  - **A**: *Ædes mcintoshi* (residing in the floodplain zone, known RVFv vector with vertical transmission and dormancy in eggs)
  - **B**: *Ædes ægypti* (residing in residential and forest zones, known RVFv vector with vertical transmission)
  - **C**: *Culex* sp.1 (residing in the floodplain, exact species currently unknown in Kilombero Valley)
  - **D**: *Culex* sp.2 (residing in the residential and forest zones, exact species currently unknown in Kilombero Valley)

*Ædes mcintoshi* floodplain populations have vertical transmission and dormant (infected and uninfected) eggs. *Æ. ægypti* populations also have vertical transmission, but no dormancy in the eggs so only the *Æ. mcintoshi* eggs sustain the infection during a drought spell. *Culex* populations have neither vertical transmission nor dormancy in the eggs. Mosquito larvæ are ignored in the model (the delay they represent is simulated by means of a lower egg eclosion rate and a higher egg mortality). *Ædes* mosquitoes generally have a lower vector competence for RVFv compared to *Culex* spp. Due to heavy rains (annual flooding and the El Niño phenomenon), the infected *Ædes* mosquito eggs hatch. The infection is quickly taken over by the *Culex* species present in that region, making an epidemic possible.

Parameter values (ranges) for this scenario are given in Tables 3, 4 and 5. The model was run for 27 years, thereby modelling three El Niño events (years 1, 11 and 21) allowing the model to reach quasi-equilibrium conditions and generating output six years after the last ENSO, which could be compared with the observations made during the field studies [17, 22].

**Table 3:** Basic model parameters—1.

Symbol	Roman	Description	Value	References	Comments
		**General**			
	year	Number of years (360 days) to run the simulation	27	user-defined	
	flood_prop	proportion flooded annually in floodplain	0.025	user-defined	
Ω_*alt*_	O_alt	Number of bites by all vector species on alternative hosts	0	user-defined	
*β*_*wl*_	b _wl	Wildlife infection rate	0	user-defined	
		**Human**			
*γ*_*H*_	g_h	Human birth rate	4/(2*50*360)	user-defined	
*μ*_*H*_	m_h	Human mortality rate	= *γ*_*H*_	user-defined	
*ξ*_*H*_	x_h	Human RVF incubation rate	1/4 (2–6 days)	[29]	
*δ*_*H*_	d_h	Human RVF-specific mortality rate	1/3*0.01	[29]	
*α*_*H*_	a_h	Human RVF recovery rate	1/3*0.99	[2, 29]	
*ρ*_*H*_	r_h	Human immunity loss rate	1/900	[41]	
$\lambda_{H}^{ij}$	l_h{ij}	Human migration rate from zone *i* to zone *j*	various^†^		
*π*_*HA*_	p_ha	Probability to transmit infection from person to *Æ. mcintoshi*	0.89 (77–100%)	[42, 43]	based on hamster model
*π*_*HB*_	p_hb	Probability to transmit infection from person to *Æ. ægypti*	0.89 (77–100%)	[42, 43]	based on hamster model
*π*_*HC*_	p_hc	Probability to transmit infection from person to *Culex* sp1	0.81 (78–84%)	[42, 43]	based on hamster model
*π*_*HD*_	p_hd	Probability to transmit infection from person to *Culex* sp2	0.81 (78–84%)	[42, 43]	based on hamster model
$\eta_{H}^{i}$	h_h{1, 2, 3}	Maximum number of bites per person per day in zone *i*	25, 25, 25	user-defined	
		**Cattle**			
$\gamma_{M_{U}}$	g_m_u	Birth rate non-infected cattle	0.00082	user-defined	
$\pi_{A_{I}}$	p_a_i	Proportion abortion due to RVF	0.90	user-defined	
$\gamma_{M_{I}}$	g_m_i	Birth rate infected cattle	$\left(1-\pi_{A_{I}}\right)\times \gamma_{M_{U}}$		
$\kappa_{M}^{i}$	k_m{1, 2, 3}	Carrying capacity cattle in zone *i*	500000	user-defined	
*μ*_*M*_	m_m	Cattle mortality rate	0.0008	user-defined	
*ξ*_*M*_	x_m	Cattle RVF incubation rate	24/3.25 (12–72 hrs)	[44]	
				[45]	based on sheep data
*δ*_*M*_	d_m	Cattle RVF-specific mortality rate	1/3*0.05	OIE disease fact sheet RVF	
*α*_*M*_	a_m	Cattle RVF recovery rate	1/3*0.95	[2]	
*ρ*_*M*_	r_m	Bovine immunity loss rate	1/900	[41]	
$\lambda_{M}^{ij}$	l_m{ij}	Cattle migration rate from zone *i* to zone *j*	various^‡^		
$\varphi_{\text{MH}}^{i}$	f_mh*i*	Number of cattle met per person per time unit in zone *i*	2.5	user-defined	
*π*_*MA*_	p_ma	Probability to transmit infection from bovine to *Æ. mcintoshi*	0.89 (77–100%)	[42, 43]	
*π*_*MB*_	p_mb	Probability to transmit infection from bovine to *Æ. ægypti*	0.89 (77–100%)	[42, 43]	
*π*_*MC*_	p_mc	Probability to transmit infection from bovine to *Culex* sp1	0.81 (78–84%)	[42, 43]	
*π*_*MD*_	p_md	Probability to transmit infection from bovine to *Culex* sp2	0.81 (78–84%)	[42, 43]	
*π*_*MH*_	p_mh00	Probability to transmit infection from bovine to people	0.001	user-defined	
*η*_*M*_	h_m	Maximum number of bites per bovine per day	50	user-defined	

^†^ Currently: _21_ = 0.005; _23_ = 0.001; _12_ = 0.05; _32_ = 0.05; _13_ = 0.0001; _31_ = 0.005

^‡^ Currently: _13_ = 0; _23_ = 0.0001; _32_ = 0.0005; _31_ = 0; _21_ and _12_ seasonal movement from plateau to floodplain

**Table 4:** Basic model parameters—2.

Symbol	Roman	Description	Value	Range	References	Comments
		***Æ. mcintoshi***				
*γ*_*A*_	g_a	*Æ. mcintoshi* egg production rate	10		expert opinion	
$\kappa_{A}^{1}$	k_a1	*Æ. mcintoshi* carrying capacity in zone 1	175000		user-defined	
*ζ*_*A*_	z_a	Probability *Æ. mcintoshi* vertical transmission	0.5^†^			
$\mu_{A_{Q}}^{1}$	m_aq1	Mortality rate *Æ. mcintoshi* infected eggs in zone 1	0.00001		[46]	
$\mu_{A_{P}}^{1}$	m_ap1	Mortality rate *Æ. mcintoshi* uninfected eggs in zone 1	0.00001		[46]	
*μ*_*A*_	m_a	*Æ. mcintoshi* adult mortality rate	1/3		expert opinion	
*ε*_*AH*_	e_ah	Proportion of *Æ. mcintoshi* feeding on people	0.1	(0.1–0.9)	[47]	adequate contact
*ε*_*AM*_	e_am	Proportion of *Æ. mcintoshi* feeding on cattle	0.3	(4/13)	[48]	% engorged based on host choice experiments
*ν*_*A*_	v_a	Number of bites per *Æ. mcintoshi* mosquito per day	0.5	(0.45–0.7)	[49]	
*π*_*AH*_	p_ah	Probability to transmit infection to person upon *Æ. mcintoshi* bite	0.01		[42, 43]	
*π*_*AM*_	p_am	Probability to transmit infection to bovine upon *Æ. mcintoshi* bite	0.01		[42, 43]	
		***Æ. ægypti***				
*γ*_*B*_	g_b	*Æ. ægypti* egg production rate	25		expert opinion	
$\kappa_{B}^{2}$	k_b2	*Æ. ægypti* carrying capacity in zone 2	175000		user-defined	
$\kappa_{B}^{3}$	k_b3	*Æ. ægypti* carrying capacity in zone 3	175000		user-defined	
*ζ*_*B*_	z_b	Probability *Æ. ægypti* vertical transmission	0.05	(0–8.5%)	[50]	
$\mu_{\text{BQ}}^{2}$	m_bq2	*Æ. ægypti* infected egg mortality rate in zone 2	0.005		[46]	
$\mu_{\text{BP}}^{2}$	m_bp2	*Æ. ægypti* uninfected egg mortality rate in zone 2	0.005		[46]	
$\mu_{\text{BQ}}^{3}$	m_bq3	*Æ. ægypti* infected egg mortality rate in zone 3	0.005		[46]	
$\mu_{\text{BP}}^{3}$	m_bp3	*Æ. ægypti* uninfected egg mortality rate in zone 3	0.005		[46]	
*τ*_*B*_	t_b	*Æ. ægypti* hatching rate	0.2		[51]	
					[52]	
*μ*_*B*_	m_b	*Æ. ægypti* adult mortality rate	0.10		user-defined	
*ε*_*BH*_	e_bh	Proportion of *Æ. ægypti* feeding on people	0.01		[53]	
					[54]	
					[55]	
					[47]	
*ε*_*BM*_	e_bm	Proportion of *Æ. ægypti* feeding on cattle	0.25		[54]	
*ν*_*B*_	v_b	Number of bites per *Æ. ægypti* mosquito per day	0.5	(0.45–0.7)	[49]	
$\lambda_{B}^{ij}$	l_b{ij}	*Æ. ægypti* migration rate from zone *i* to zone *j*	0		user-defined	
*π*_*BH*_	p_bh	Probability to transmit infection to person upon *Æ. ægypti* bite	0.01		[42, 43]	Based on Hamster model
*π*_*BM*_	p_bm	Probability to transmit infection to bovine upon *Æ. ægypti* bite	0.01		[42, 43]	Based on Hamster model

^†^ Values within the published range [0—8.5%, [50]] did not allow infection to be carried by dormant *Æ. mcintoshi* eggs from one El Niño event to the next

**Table 5:** Basic model parameters—3.

Symbol	Roman	Description	Value	Range	References	Comments
		***Culex* sp.1**				
*γ*_*C*_	g_c	*Culex* sp1 egg production rate	25		expert opinion	
$\kappa_{C}^{1}$	k_c1	*Culex* sp1 carrying capacity in zone 1	1750		user-defined	
$\mu_{\text{CP}}^{1}$	m_cp1	*Culex* sp1 egg mortality rate in zone 1	0.002		user-defined	
*τ*_*C*_	t_c	*Culex* sp1 hatching rate	0.2		user-defined	
*μ*_*C*_	m_c	*Culex* sp1 adult mortality rate	0.10		user-defined	
*ε*_*CH*_	e_ch	Proportion of *Culex* sp1 feeding on people	0.005		[47]	depends on host availability
*ε*_*CM*_	e_cm	Proportion of *Culex* sp1 feeding on cattle	0.02	(0–0.9)	[47, 48]	host availability and host choice experiments
*ν*_*C*_	v_c	Number of bites per *Culex* sp1 mosquito per day	1		user-defined	
*π*_*CH*_	p_ch	Probability to transmit infection to person upon *Culex* sp1 bite	0.07	(7–37%)	[42, 43]	based on hamster model
*π*_*CM*_	p_cm	Probability to transmit infection to bovine upon *Culex* sp1 bite	0.07	(7–37%)	[42, 43]	based on hamster model
		***Culex* sp.2**				
*γ*_*D*_	g_d	*Culex* sp2 egg production rate	25		expert opinion	
$\kappa_{D}^{2}$	k_d2	*Culex* sp2 carrying capacity in zone 2	17500		user-defined	
$\kappa_{D}^{3}$	k_d3	*Culex* sp2 carrying capacity in zone 3	17500		user-defined	
$\mu_{\text{DP}}^{2}$	m_dp2	*Culex* sp2 egg mortality rate in zone 2	0.002		user-defined	
$\mu_{\text{DP}}^{3}$	m_dp3	*Culex* sp2 egg mortality rate in zone 3	0.002		user-defined	
*τ*_*D*_	t_d	*Culex* sp2 hatching rate	0.2		user-defined	
*μ*_*D*_	m_d	*Culex* sp2 adult mortality rate	0.10		user-defined	
*ε*_*DH*_	e_dh	Proportion of *Culex* sp2 feeding on people	0.005	(0–0.9)	[47]	
*ε*_*DM*_	e_dm	Proportion of *Culex* sp2 feeding on cattle	0.12	(0–0.9)	[47, 48]	host availability and host choice experiments
*ν*_*D*_	v_d	Number of bites per *Culex* sp2 mosquito per day	1		user-defined	
$\lambda_{D}^{ij}$	l_d{ij}	*Culex* sp2 migration rate from zone *i* to zone *j*	0		user-defined	
*π*_*DH*_	p_dh	Probability to transmit infection to person upon *Culex* sp2 bite	0.07		[42, 43]	
*π*_*DM*_	p_dm	Probability to transmit infection to bovine upon *Culex* sp2 bite	0.07		[42, 43]	

## Results

**Fig 3:**
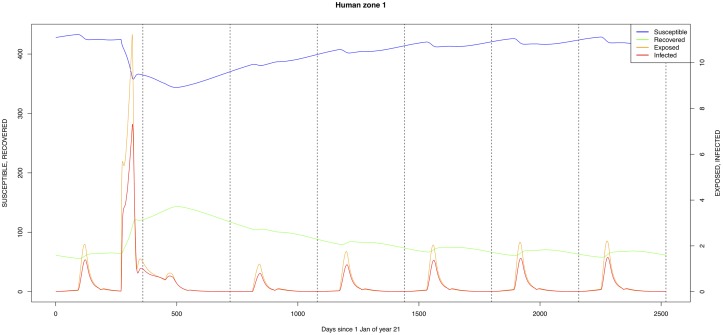
Standard parameters: Human—Zone 1.

**Fig 4:**
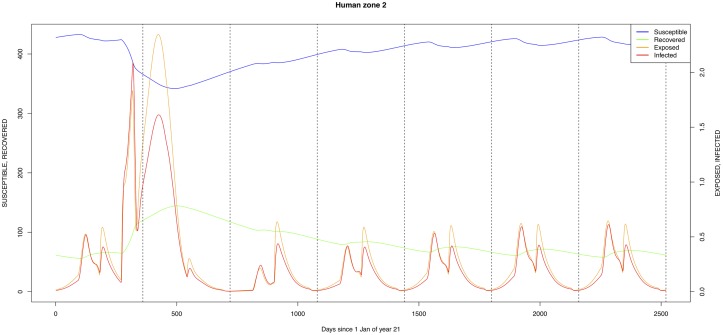
Standard parameters: Human—Zone 2.

**Fig 5:**
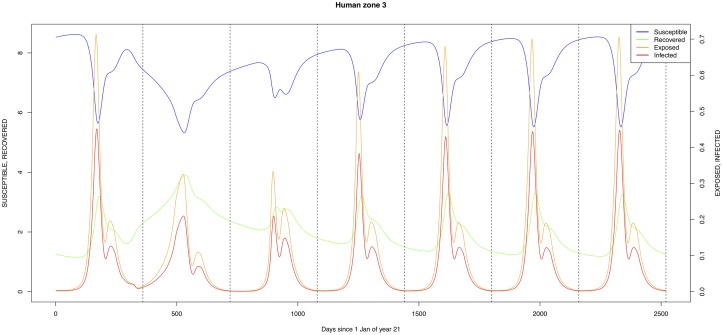
Standard parameters: Human—Zone 3.

**Fig 6:**
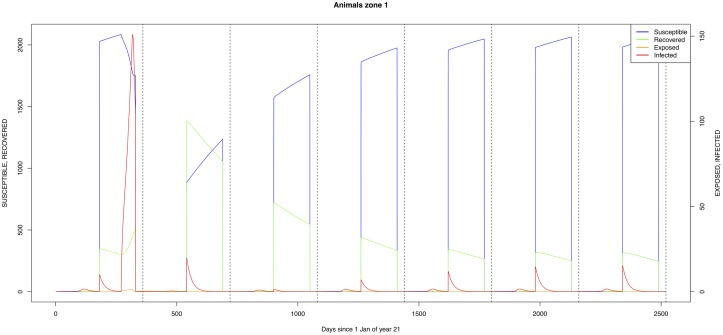
Standard parameters: Cattle—Zone 1.

**Fig 7:**
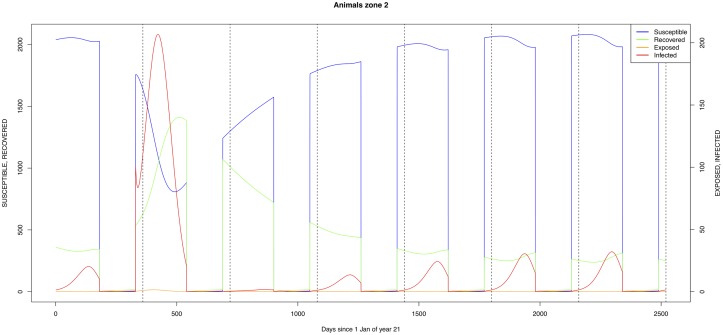
Standard parameters: Cattle—Zone 2.

**Fig 8:**
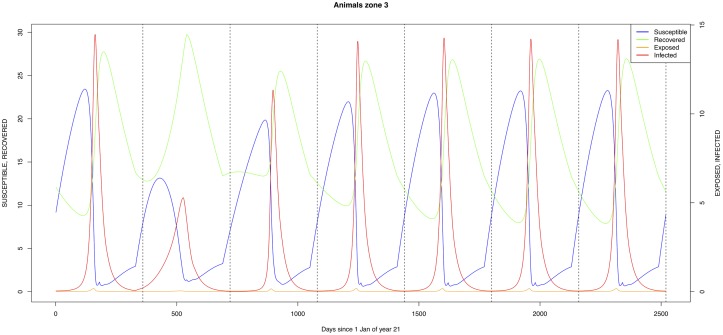
Standard parameters: Cattle—Zone 3.

**Fig 9:**
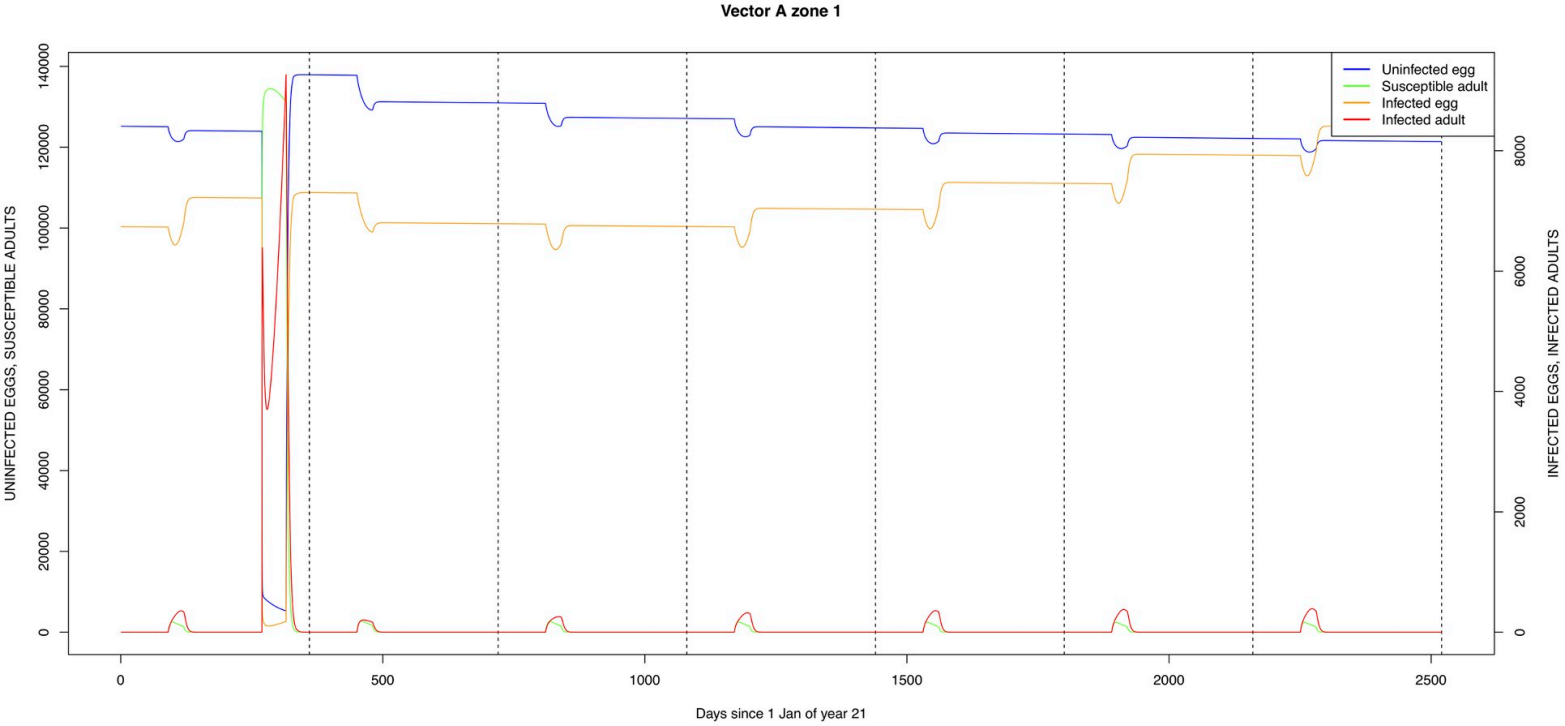
Standard parameters: *Æ. mcintoshi*—Zone 1.

**Fig 10:**
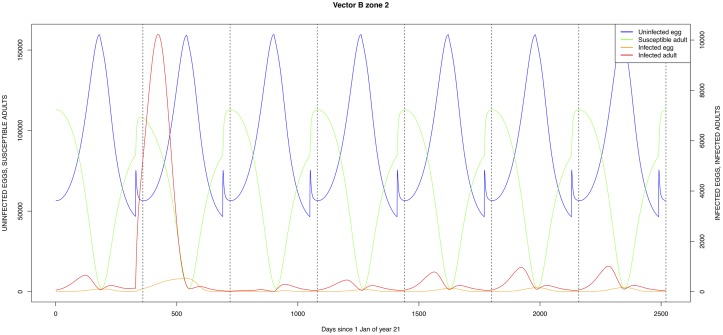
Standard parameters: *Æ. ægypti*—Zone 2.

**Fig 11:**
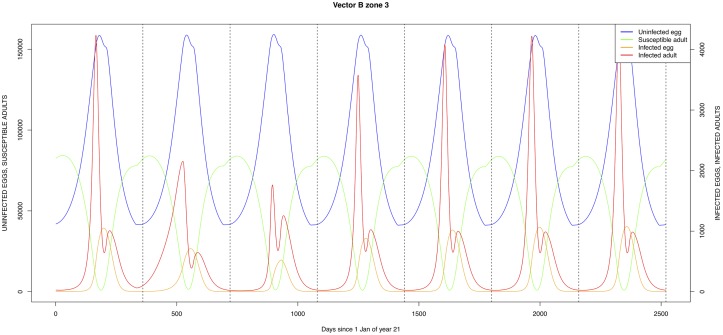
Standard parameters: *Æ. ægypti*—Zone 3.

**Fig 12:**
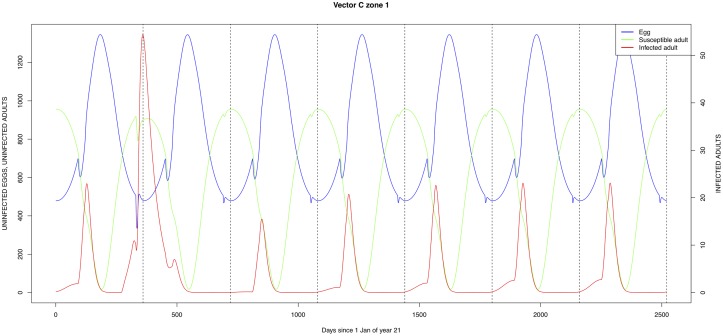
Standard parameters: *Culex* sp.1—Zone 1.

**Fig 13:**
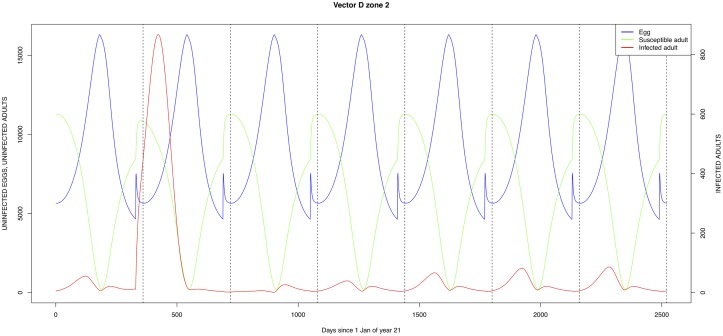
Standard parameters: *Culex* sp.2—Zone 2.

**Fig 14:**
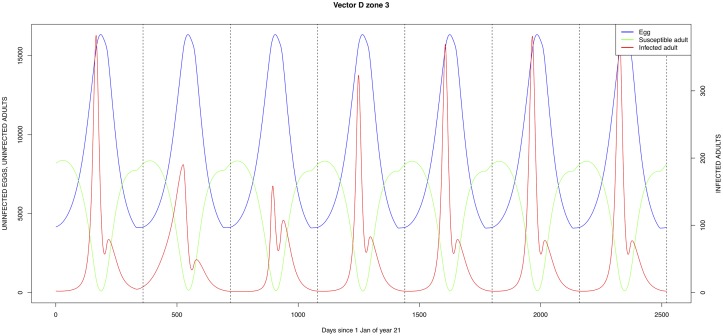
Standard parameters: *Culex* sp.2—Zone 3.

**Fig 15:**
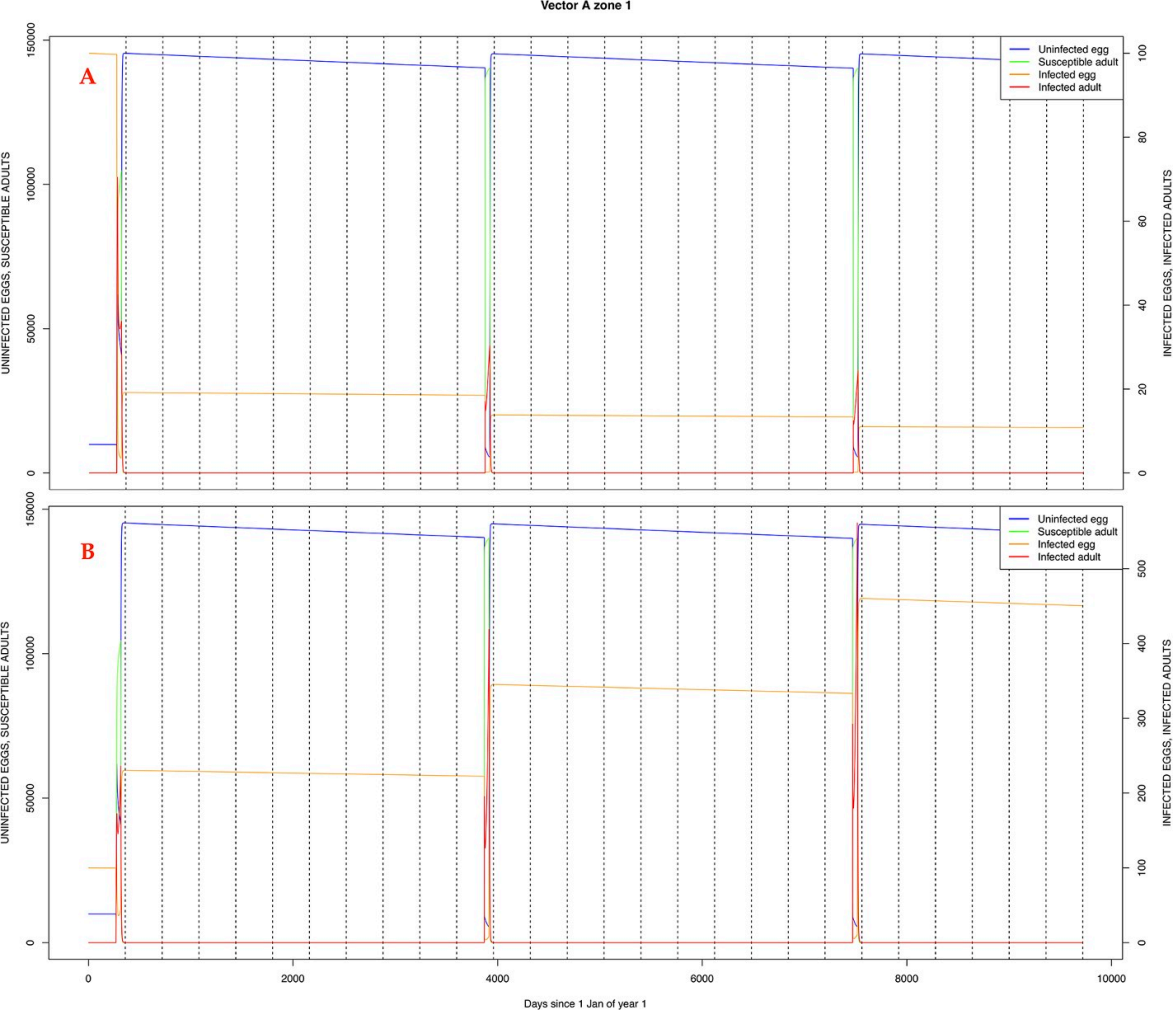
*Æ. mcintoshi* as only vector, no seasonal flooding of zone 1. A: Vertical transmission rate = 0.25; B: Vertical transmission rate = 0.50.

The graphical output (showing results for the years 20–27) for the simulations over a period of 27 years, using the standard parameter values as shown in Tables 3–5 are presented in Figs 3–14. The graphical output for the *Æ. mcintoshi* population in zone 1, when this is the only vector and when there is no seasonal flooding of the plains in this zone is shown in Fig 15: the importance of the level of vertical transmission within the *Ædes* population is shown in the respective sub-figures of Fig 15. The seroprevalence levels in the human and cattle population at different years after the El Niño event of year 21 are shown in Table 6.

**Table 6:** RVF seroprevalence levels (proportion) in people and cattle at different times after an El Niño event.

	Human			Cattle		
	EN+2^†^	EN+4	EN+6	EN+2	EN+4	EN+6
Standard	0.209	0.147	0.132	0.324	0.140	0.123
Standard + wl	0.209	0.147	0.132	0.324	0.139	0.122
only Aemc (100 $A_{Q}^{1}$ + 9900 $A_{P}^{1}$) − flood	0.005	0.002	0.001	0.003	0.001	0.000
only Aemc (100 $A_{Q}^{1}$ + 9900 $A_{P}^{1}$) + flood	0.136	0.093	0.078	0.063	0.017	0.006
only Aeae (100 $B_{Q}^{2}$ + 9900 $B_{P}^{2}$)	0.048	0.041	0.039	0.070	0.067	0.067
only Cu2 (1000 $D_{P}^{3}$)	0.000	0.000	0.000	0.000	0.000	0.000
only Cu2 (1000 $D_{P}^{3}$) + wl	0.130	0.138	0.141	0.034	0.035	0.035
only Cu2 (1000 $D_{P}^{2}$)	0.000	0.000	0.000	0.000	0.000	0.000
only Cu2 (1000 $D_{P}^{2}$) + introduction of 1 $M_{I}^{2}$	0.177	0.186	0.189	0.132	0.136	0.136

^†^EN+2/4/6 = year 2/4/6 after El Niño event
Standard: 1000 $H_{S}^{2}$, 2500 $M_{S}^{2}$, 100 $A_{Q}^{1}$, 9900 $A_{P}^{1}$, 10 $B_{P}^{3}$, 100 $C_{P}^{1}$, 1000 $D_{S}^{2}$, 1000 $D_{P}^{3}$ Standard + wl: as above + wildlife reservoir (infection rate for vectors = 1e^-5^) only Aemc (100 $A_{Q}^{1}$ + 9900 $A_{P}^{1}$) − flood: *Æ. mcintoshi* 100 infected eggs, 9900 uninfected eggs in zone 1, no annual partial flooding of zone 1 only Aemc (100 $A_{Q}^{1}$ + 9900 $A_{P}^{1}$) + flooding: as above + annual partial flooding of zone 1 only Aeae (100 $B_{Q}^{2}$ + 9900 $B_{P}^{2}$): *Æ. ægypti* 100 infected eggs, 9900 uninfected eggs in zone 2 only Cu2 (1000 $D_{P}^{3}$): *Culex* sp.2 1000 eggs in zone 3 only Cu2 (1000 $D_{P}^{3}$): as above + wildlife reservoir (infection rate for vectors = 1e^-5^) only Cu2 (1000 $D_{P}^{2}$): *Culex* sp.2 1000 eggs in zone 2 only Cu2 (1000 $D_{P}^{2}$) + introduction of 1 $M_{I}^{2}$: as above with introduction of one infective bovine in Zone 2

## Discussion

A model on RVFv transmission in the Kilombero valley in Tanzania was run for 27 years to include three El Niño events (and thus three RVF epidemics), to allow the model to reach a state of ‘equilibrium’ and to allow model output during a period of 4-7 years after the epidemic to coincide with published observations [17, 22]. The model is a complex interaction of density-dependent birth, death and transmission processes and as such very sensitive to certain parameter values. The model was explored by means of scenarios and no attempt was made to include a sensitivity analysis.

Most parameters could be kept at values within the ranges found in the literature, by adjusting the values of other parameters to acceptable values, based on expert opinion. In this respect, a major influence is exerted by *ν*, the maximum number of bites ‘supported’ by an individual host. The value itself directly determines the (*e.g*.) seroprevalence levels, but this parameter also introduces a competition between the various vector species, as at present it is assumed that the ‘available’ bites are distributed proportionally between the different vectors. The effect can be seen in Table 6, when comparing lines one and (*e.g*.) nine: *Culex* on its own, being a more efficient vector, yields higher seroprevalence values than the standard setting, where it must share the biting opportunities with *Ædes*.

The exception to the above was the vertical transmission rate (trans-ovarial transmission rate) for *Æ. mcintoshi*. The range found in [50] (0–8.5%) is not sufficient to carry the virus from one epidemic to another in the absence of other vectors to ensure inter-epidemic transmission. As shown in Fig 15, a vertical transmission rate of 0.25 does not suffice to ensure sufficient numbers of infected eggs to trigger an epidemic at the next El Niño event. No other estimates of this parameter could be traced in the literature and it is recommended that the correct values (ranges) of this important parameter are determined experimentally.

A low level of RVFv transmission was predicted by the model (Table 6). Using the standard values, predicted seroprevalence levels in humans and cattle at different times after the El Niño event were comparable to those observed. Seroprevalence is estimated to be 13.2% in people and 12.3% in cattle, six years after an El Niño event. The field studies found similar overall seroprevalence levels of 11.7% in people and 11.3% in cattle, five to six years after the 2006/07 RVF epidemic in the area [17, 22]. The results are also in line with previous studies across Africa with evidence of inter-epidemic transmission of RVF [1, 15, 16]. The dynamics of levels of seroprevalence are of course in the first place dependent on the value employed for the loss-of-serotitre rate: currently a daily value of 1/900 is used, based on a single, rather vague reference [41]. Inclusion of a wildlife reservoir (Table 6, second line) did not have a significant effect on the predicted levels of seroprevalence.

The simulated seroprevalence levels in Table 6 in both the human and livestock populations show a gradual decline during the years after an epidemic event (El Niño), which seems to imply low numbers of infective bites during inter-epidemic periods, reflecting the generally low numbers of mosquitoes in the absence of heavy rainfall associated with the El Niño events. People and cattle transiting in the forest (zone 3, Figs 5 and 8) are exposed to infectious bites every year from the *Æ. ægypti* and *Culex* sp.2 populations (Figs 11 and 14): the mosquitoes are constantly infected from the wildlife reservoir [56]. People and cattle remaining in the villages (zone 2, Figs 4 and 7) and/or the floodplains (zone 1, Figs 3 and 6) are minimally exposed on an annual basis with high exposure rates occurring only every ten years (Figs 9, 10, 12 and 13). Infection thus principally spreads to the villages and floodplains by humans and cattle temporarily residing in the forest zone.

The *Æ. mcintoshi* population in the floodplains (Fig 9) is the one maintaining the infection inside the dormant eggs. Adult mosquitoes do not survive the drier period following the El Niño event and only some eggs hatch every year during the partial seasonal flooding of the plain. Substantial hatching occurs during flooding related to the El Niño event in the East African region, releasing the infection and starting the epidemics. The infection is picked up by *Culex* sp.1 present in this area. The human population acquires the infection first, followed by the cattle population. From there on, the epidemic spreads to the village and the forest with migrating cattle and people.

As indicated by lines three and four of Table 6 (with the current standard parameter settings), *Æ. mcintoshi* on its own is not able to explain the high seroprevalence found in both humans and cattle [17, 22], not even when including annual partial flooding of zone 1 accompanied by eclosion of part of the dormant eggs. The same can be said for *Æ. ægypti*, despite it being resident in the village and forest zones, although it must be understood that in this case the low values for vertical transmission were maintained.

Lines six to nine of Table 6 examine different scenarios with an efficient *Culex* vector in the village and forest zones. Introduction of infection, either by means of a wildlife reservoir (line seven) or through the introduction of an infective animal, allows for maintenance of the infection within the host and vector populations. Because of the interaction between the different vectors for host-feeding opportunities, the more efficient *Culex* vector on its own (without competition from *Aedes* species) results in higher infection transmission and higher seroprevalence levels. Again, a lot more detailed observations are required to properly quantify this aspect of the transmission dynamics.

Mosquito species in the forested environment (*Æ. ægypti* and *Culex* sp.2) (Figs 11 and 14) had high annual infection rates. On the other hand, mosquitos in the residential area (*Æ. ægypti* and *Culex* sp.2) and in the floodplain (*Æ. mcintoshi* and *Culex* sp.1) have low infection rates (Figs 9, 10, 12 and 13) with peak rates occurring only during or immediately after an El Niño event and subsequent RVF epidemics in the East African region [57].

The model presented here needs further calibrating with datasets from other regions where there are similar or dissimilar ecologies compared to our study area in order to extend and/or improve usability of the model in different geographical, climatic settings. This model, being built with open-source software and with an easy to use interface, can be adapted by researchers and program managers to their specific needs by plugging in new parameters relevant to their situation and locality. Its use can be further expanded by including disease prevention and control interventions to model potential impact of these veterinary and public health measures on disease in people and domestic animals, for example vaccination, quarantining and vector control programs.

## Supporting information

S1 Appendix User manual. (PDF)

S2 Appendix Program R code. (PDF)
