# Supplementary material for: Rift Valley fever: An open-source transmission dynamics simulation model
Source: PLoS One. 2019 Jan 9;14(1):e0209929. doi: 10.1371/journal.pone.0209929 (PMC6326482; doi:10.1371/journal.pone.0209929)
Supplement: S1 Appendix — (PDF) [file pone.0209929.s001.pdf]

RVF transmission dynamics

User manual

Contents

1 Introduction . . . 1
2 Installation . . . 2
2.1 Installation of **R**™ and **RStudio**™ . . . 2
2.2 Installation of the RVF components . . . 3
2.2.1 Fast C++ version . . . 3
2.2.2 Slow open version . . . 3
3 Starting the program . . . 4
4 Setting up the simulation . . . 5
4.1 Introduction . . . 5
4.2 Run simulation & select plots . . . 5
4.3 Initial state . . . 5
4.4 General model information . . . 6
4.5 People . . . 8
4.6 Animal . . . 8
4.7 Vector A . . . 9
4.8 Vector B . . . 9
4.9 Vector C . . . 9
4.10 Vector D . . . 9
4.11 Summary tables . . . 9
5 Running the simulation . . . 10
6 Saving the graph output . . . 11
7 Stopping the program . . . 11
8 Sample output . . . 12

1 Introduction

Two versions of the simulation Rift Valley fever virus (RVFv) transmission dynamics package are offered. They are, on the one hand, a faster, less user-friendly compromise and, on the other, a completely open, easily adapted but rather slow version (about 60 times slower than the first version). The compromise version has the actual model code written in C++ with input and output through RStudio shiny. The completely open version has the entire code (model computations, input and output) written in R.

2 Installation

2.1 Installation of R™ and RStudio™

1. Obtain and install the most recent versions of **R**™ and **RStudio**™

> **Attention!**
> It is important to always ensure both R and RStudio are up-to-date as conflicts between different packages may occur if this is not the case.

2. Ensure the latest version of each of the following packages is downloaded and installed:

   - `shiny`
   - `deSolve`
   - `Rcpp`

> **Attention! If C^{++} is not installed**
> Test to see if C^{++} language components are correctly installed by first running the short script `test.R` (Listing 1). If C^{++} is properly installed the message `Rcpp installed 0` will appear. If not:
>
> - MacOS: users must have local copies of the **xcode developer line tools**. If not installed, follow the instructions at: https://thecoatlessprofessor.com/programming/r-compiler-tools-for-rcpp-on-os-x/
> - Windows: users must have a local install of **Rtools**. If not installed, when running Listing 1 the ensuing error will automatically open the following wizard. Follow the instructions.

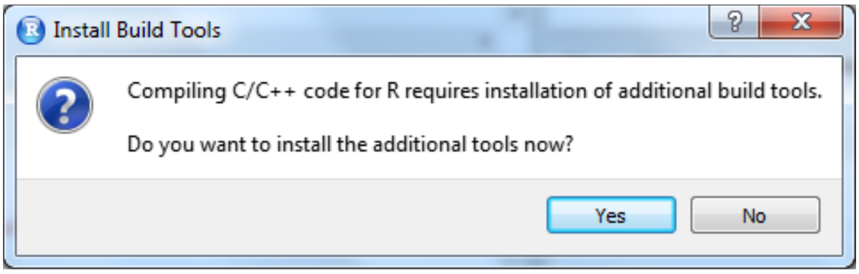

Listing 1: Test C^{++} installation

```
require(Rcpp)
cppFunction('void test(int m) { std::cout<<"Rcpp installed "<<m<<std::endl;}')
test(0)
```

2.2 Installation of the RVF components

2.2.1 Fast C^{++} version

Ensure the following files are present, all in the same folder/directory:

- `RVF.R`
- `CppFunctions.cpp`
- `CppFunctions.h`

Attention!

Any name, allowed by the respective operating system, can be used for the enclosing directory/folder. However, it is essential **not to change the names** of the individual files. An error will occur when any of the file names are changed. An error also occurs when the three files are **not in the same directory/folder**.

2.2.2 Slow open version

Ensure the `RVF_nocpp.R` is installed.

3 Starting the program

1. Open `RVF.R` (or `RVF_nocpp.R`) in **RStudio**

2. Select `RVF.R` (or `RVF_nocpp.R`): the top right of the script pane (which is the top left window of RStudio in the standard set-up) should show a green arrow with the text `Run App`

3. Click the small triangle to the right of `Run App` and select **Run in Viewer Pane** from the drop-down menu[1]

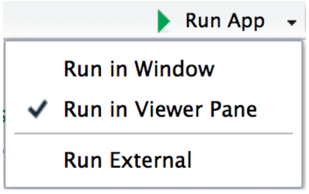

4. Click the **Viewer** tab in the right bottom window pane and maximise its size by moving the horizontal divider to the top and the vertical divider as far left as possible, making sure the green arrow in the script pane remains visible (once the program runs, the divider can be moved further left to increase the size of the Viewer pane even more)

5. Click the green arrow in the script pane to start the program

[1] It may happen that you have to try and select **Run in Viewer Pane** several times. If the ✓ does not appear, select first **Run External** and then again **Run in Viewer Pane**.

4 Setting up the simulation

4.1 Introduction

Parameter and initial population values can be defined by means of numeric entry fields, sliders and checkboxes, divided over the different tab panes. Sliders can be moved by selecting the slider knob with the cursor and moving it. When the slider knob has been selected it can also be moved by means of the left and right arrows on the keyboard. The individual tab panes can be selected from the tab menu at the top of the Viewer Pane.

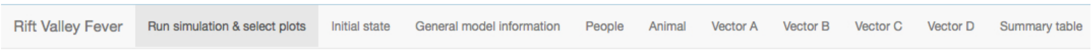

The time-unit of the actual model is one day. For practical reasons, simulation length is set in terms of years. Years start of 1 January and are subdivided in twelve months of 30 days each, *e.g.* 1 March is day 61, 1 June is day 181 and 31 December is day 360.

4.2 Run simulation & select plots

Select the plots to be generated, select the type of x-axis (either labels for each month or a continuous variable showing the number of days lapsed since the start of the plot), indicate the range to be plotted (first and last days), set the number of years over which the simulation is to be run, if desired set the random number seed and run the simulation (of course, after having set whatever parameters to the desired values).

4.3 Initial state

Set the initial values (the number of individuals in a particular compartment) for the various compartments of the model. The first letter indicates the organism (human/-mammal/vector)

- H = people
- M = domestic animals
- A = vector A
- B = vector B
- C = vector C
- D = vector D

The second letter indicates the compartment the individuals are in:

- S = susceptible (adult in case of vector)
- E = exposed
- I = infective (adult in case of vector)
- R = recovered

- Q = infected eggs
- P = uninfected eggs

All compartments may be selected for the three zones.

4.4 General model information

The overall characteristics of the simulation is determined by means of checkboxes and numerical input fields. The different options are:

- **El Niño flooding**: yes/no checkbox and, if selected, starting and ending dates of flooding
  El Niño rains causing full flooding of Zone 1 is at present set to occur every ten years (year 1, year 11, year 21, . . .)
- **Annual flooding**: yes/no checkbox and, if selected, starting and ending dates plus the proportion of Zone 1 flooded
  This type of partial flooding occurs annually at the same period
- **Seasonal effect hatching**: yes/no checkbox, peak shifting from away 1 January and number of peaks
  The standard setting (if selected) generates a single cosine curve per year with peak on 1 January. The following graphs show the output with different settings of `delay` and `number`:
  - **(a)** delay = 0; number = 1; (one peak on 1 January)
  - **(b)** delay = -90; number = 1; (one peak on 1 April)
  - **(c)** delay = 90; number = 1; (one peak on 1 September)
  - **(d)** delay = 0; number = 2 (two peaks, one on 1 January and one on 1 June)

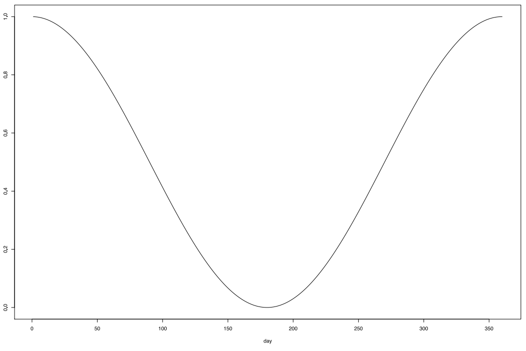

(a)

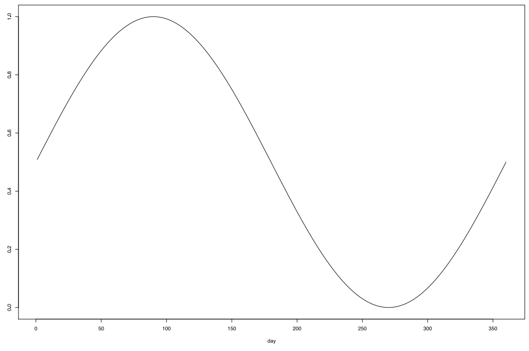

(b)

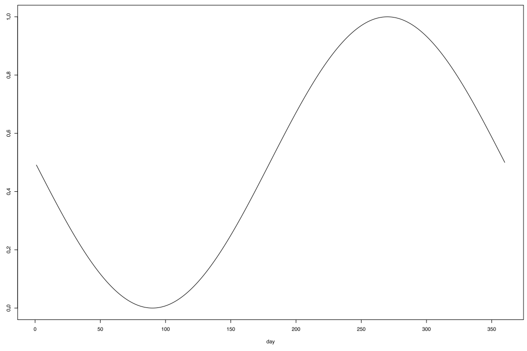

(c)

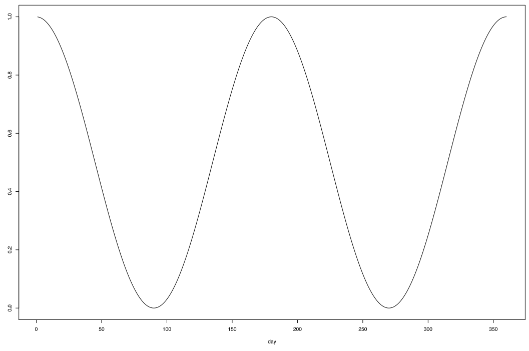

(d)

- **Annual variation climate**: yes/no checkbox and, if selected, number of `dry years` in a period of `total years` and effect on hatching (*i.e.* proportion hatching during dry year compared to normal year)
  This control option allows for inter-year climatic variability to be included, creating 'wet' and 'dry' years. A random number between zero and one is generated for every year in the simulation. If this random number is smaller than the ratio `dry years/total years` then the corresponding year suffers from drought. The hatching rate during a dry year is the normal hatching rate times the value entered by the `Minimum` slider

- **Annual transhumance**: yes/no checkbox and, if selected, date of Zone 1 to Zone 2 movement and date of return movement
  Transhumance is supposed to be an annual event currently taking place each year on the same dates. Transhumance is programmed to be from wet-season grazing around the homestead (Zone 2) to dry-season grazing on seasonally inundated pasturelands (Zone 1)

- **Increased susceptibility of animals**: yes/no checkbox and, if selected, starting and ending dates of period of increased susceptibility and factor of increase
  This option allows for the inclusion of an annual period where the animals are more prone to (*e.g.*) being bitten[2] by a vector, for instance after shearing of sheep

- **Infection rate wildlife**: infection rate of wildlife in Zone 3
  Vector species residing in Zone 3 can become infected from 'wildlife' species and in turn infect visiting people and domestic animals

[2]Bite/biting etc. are used throughout. Replace by sting/stinging or equivalent where necessary.

- **Number alternative hosts**: number of alternative hosts (*i.e.* other than people and domestic animals) available for the vectors (per zone)
  This number represents the number of alternative hosts for the vectors that can be bitten if there are not enough people and/or animals to accommodate the total number of vector bites per unit in the respective zone. Increasing this number increases the survival rate of the vectors in case of insufficient hosts, but decreases infection rates of the vectors as these alternative hosts are assumed refractory to RVFv

4.5 People

Values for parameters pertaining to the human population are entered in this pane.

- Birth rate and natural mortality rate (assumed to be the same in all zones)
  Enter the same value for birth rate and natural mortality rate to obtain a constant population size in absence of disease
- Incubation and infective periods and disease-specific mortality rate
- Migration rates between the different zones
  Put repective migration rates and initial state compartment values to zero to confine people to certain zones
- RVFv transmission probabilities from people to the different vectors
  This is the probability to transmit the virus when a vector successfully bites an infective person
- Contact rates with animals per time unit (day)
  This value represents the number of (different) animals a person contacts per day, sufficiently close for infection to be acquired from the animal, if the latter is infective
- Maximum daily biting rates
  The maximum number of bites a person 'supports' per time unit

4.6 Animal

The domestic animal population parameters are entered in this pane. They mostly correspond to what has been discussed in subsection 4.5 and only the differences are discussed.

- The standard birth rate pertains to non-infective animals. The birth rate for infective animals is adjusted taking into account the abortion rate due to infection
- There is a so-called animal population carrying capacity for the different zones. Although this affects the reproduction in a density-dependent way by reducing birth rates to zero as the number of individuals approach the carrying capacity, this upper limit can also be used to limit the animal population to a certain number, thereby (*e.g.*) simulating sales of animals in function of number of animals present

4.7 Vector A

Vector A is characterised by both the ability of eggs to remain dormant during inimical conditions (*e.g.* between El Niño events or between annual flooding) and the possibility of vertical transmission of RVFv, *i.e.* infected females produce infected eggs resulting in a new generation of infective adults. The following parameters can be set:

- Oviposition, vertical transmission and hatching rates
  The hatching rate in Zone 1 can be controlled by manipulating flooding (El Niño and annual) and by means of annual and seasonal variability in the three zones

- Mortality rates for adults and the two types of eggs in the three zones

- Daily maximum biting rate, feeding distribution[3] and RVFv transmission probabilities upon successful biting
  The feeding distribution represents the proportion of bites given to a particular host

- Carrying capacities and migration rates
  See subsection 4.5 on how to constrain vector populations to certain zones

4.8 Vector B

The parameters to be entered for this vector are essentially the same as for Vector A. The difference is that there is no dormancy in the eggs stage for this species.

4.9 Vector C

This species differs from Vector B in that there is no vertical transmission. The other parameters are equivalent.

4.10 Vector D

See subsection 4.9

4.11 Summary tables

A summary of the different compartment sizes, seroprevalence levels, maximum epidemic size etc are provided. At present, there is a table summarising compartment sizes for humans and domestic animals (first lines), as well as maximum epidemic sizes (**Infective** second line) and average seroprevalence (**Recovered** second line). A second table shows the average compartment sizes of the various vector populations. A third table provides details of the seroprevalence in human and domestic animal populations, three, five and seven years after the last El Niño event in the simulation.

[3] It is the user's responsibility to ensure that the sum of the proportions feeding on the different hosts is less than or equal to unity

5 Running the simulation

1. Set up the parameters as required
2. Click the **Run simulation** button in the **General and plot** tab pane
3. The differential equation model runs for the required time period and creates the requested sets of graphs in the **Plots** tab pane of the **RStudio** output pane. The graphs are created in individual plot windows that can be accessed by means of left and right arrows at the top left of the **Plots** pane. Which graphs are produced is selected by means of checkboxes in the **General and plot** tab pane.

> **Attention!**
>
> Although it is possible to remove the graphs in the **Plots** pane (by clicking 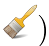), this feature is apparently not available when the App is running. It can thus apparently happen that **RStudio** runs out of memory and hangs if ‘too’ many graph windows have been created. It is therefore recommended to leave the application every now and then, clear the plots and start it up again. It is also recommended to only plot the graphs that are of interest when comparing different scenarios.

4. To go back to the input and output window click the **Viewer** tab.

6 Saving the graph output

Individual graphs can be saved in various formats. Click the small black arrow to the right of `Export` and select the desired format. Make sure that text is reproduced correctly, as especially legend text is sometimes saved incompletely. The safest option is to select **Copy to Clipboard. . .** (make sure ☑ Maintain aspect ratio is selected and do not forget to click `Copy plot`).

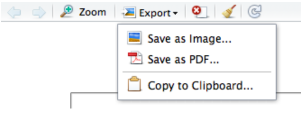

7 Stopping the program

The program is stopped by clicking STOP either in the **Console** or in the output pane.

8 Sample output

The following output is produced with the standard settings (Figure 2 to Figure 14).

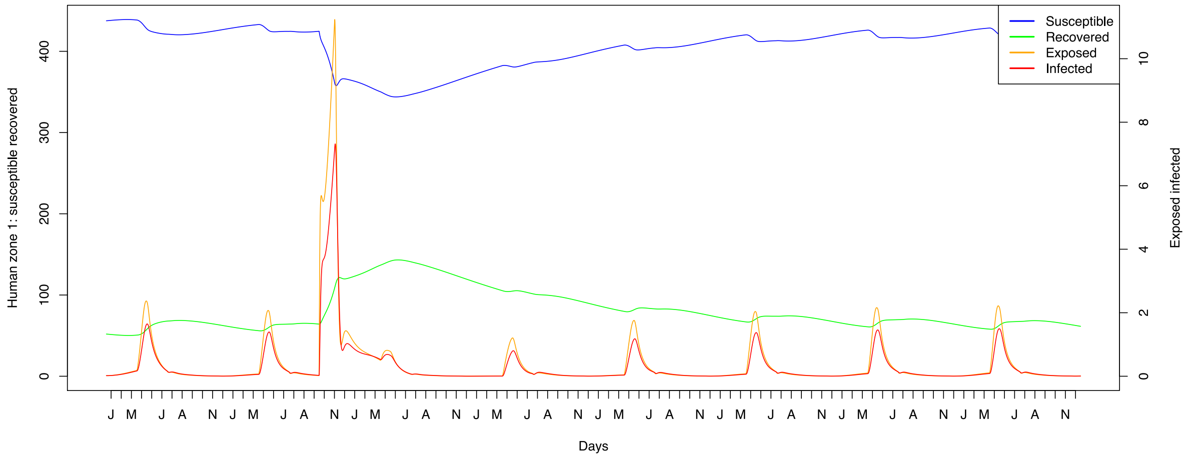

Figure 2: Standard parameters: Human - zone 1

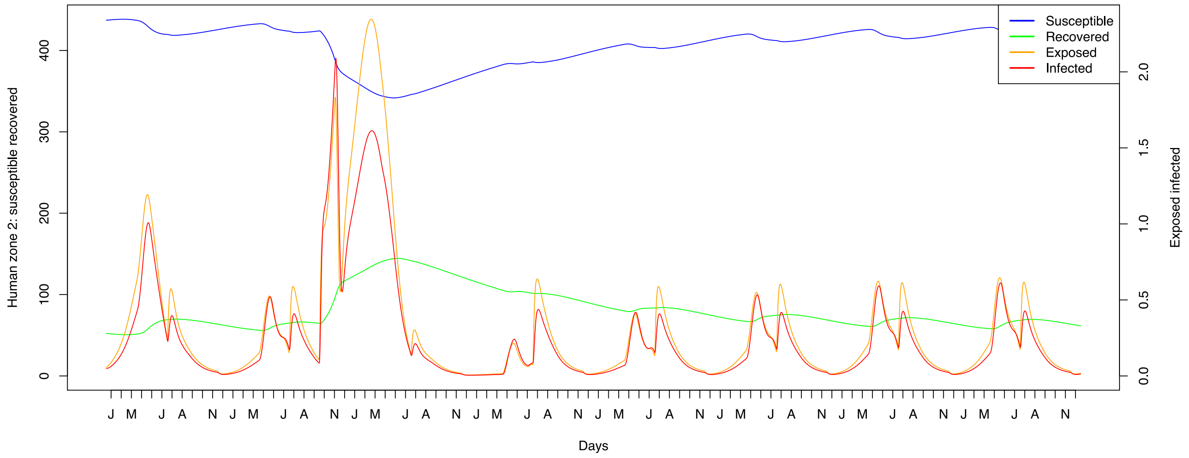

Figure 3: Standard parameters: Human - zone 2

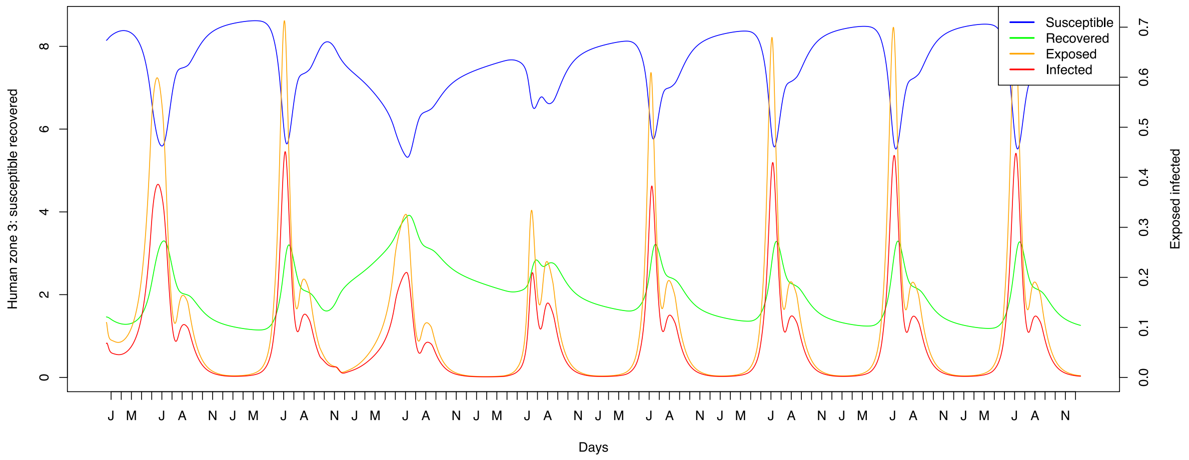

Figure 4: Standard parameters: Human - zone 3

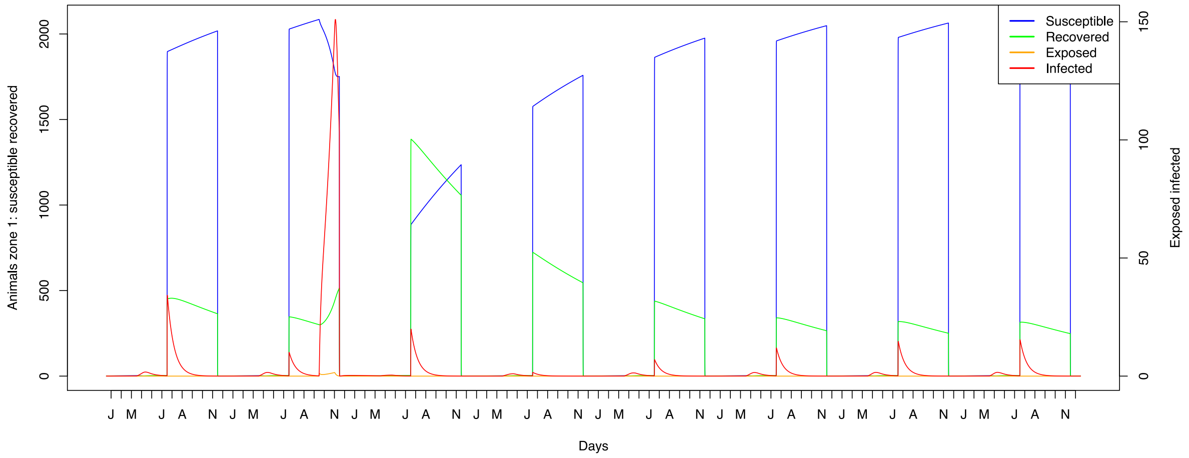

Figure 5: Standard parameters: Animal - zone 1

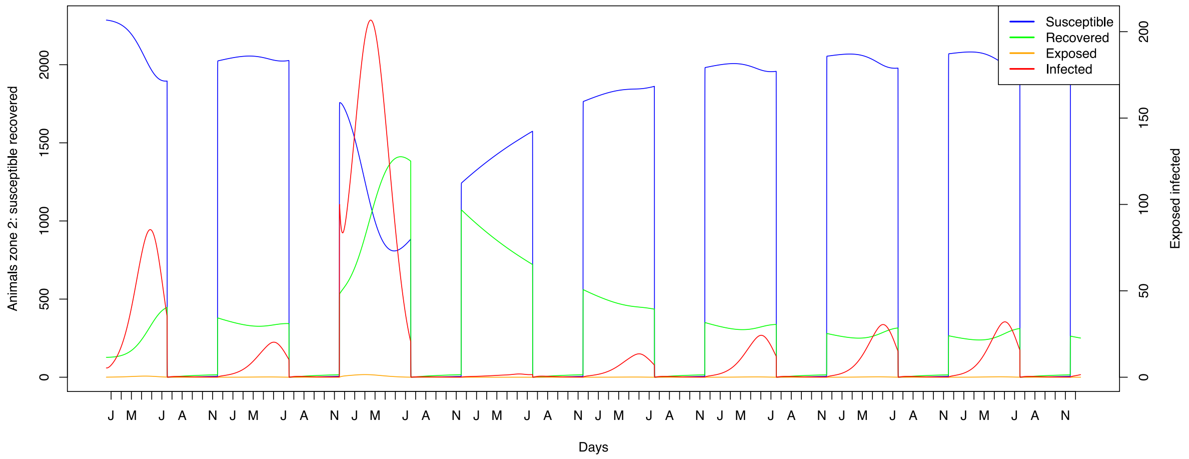

Figure 6: Standard parameters: Animal - zone 2

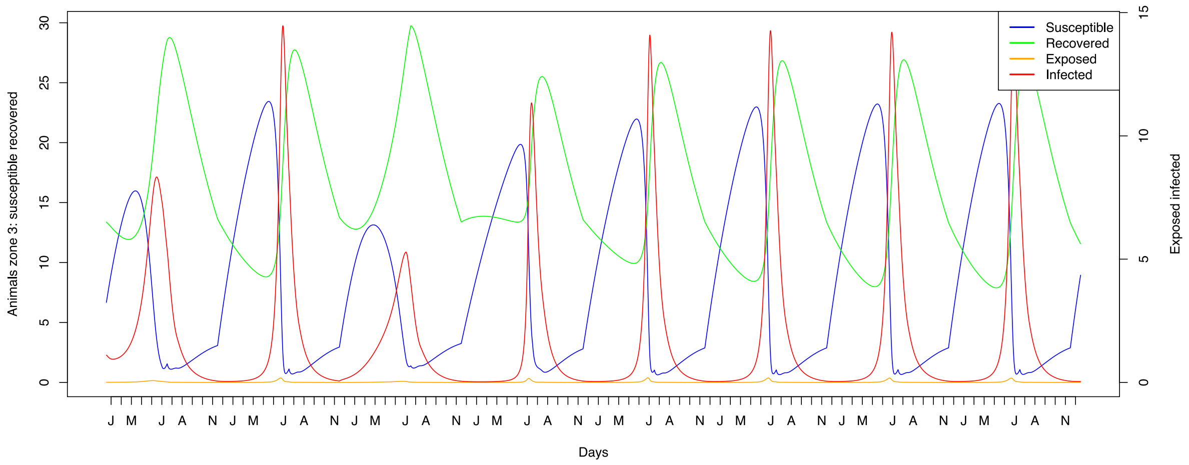

Figure 7: Standard parameters: Animal - zone 3

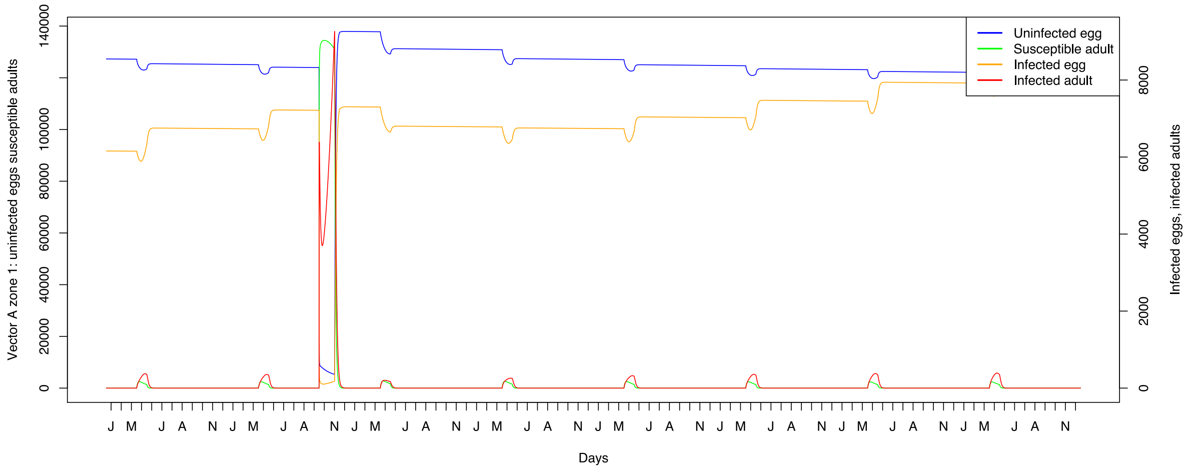

Figure 8: Standard parameters: Vector A - zone 1

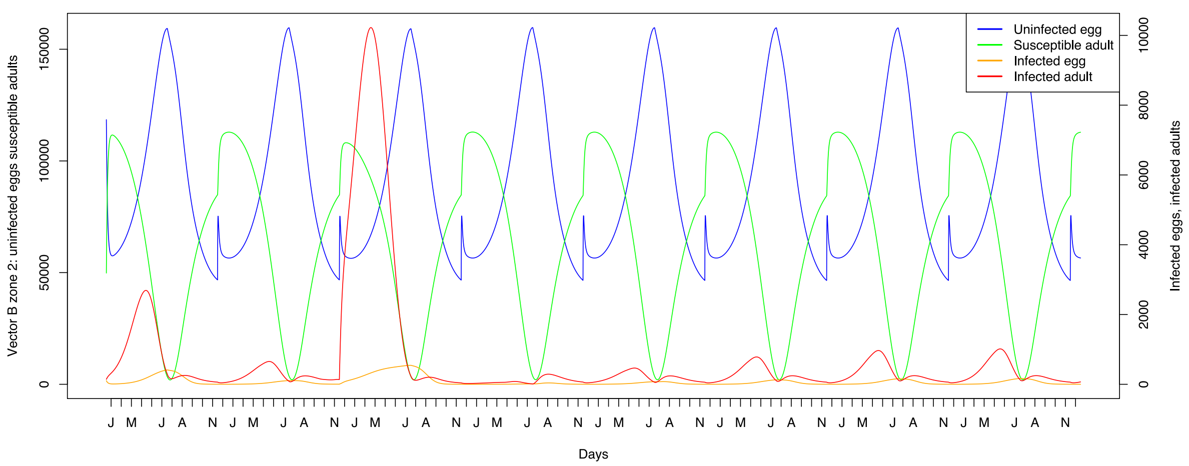

Figure 9: Standard parameters: Vector B - zone 2

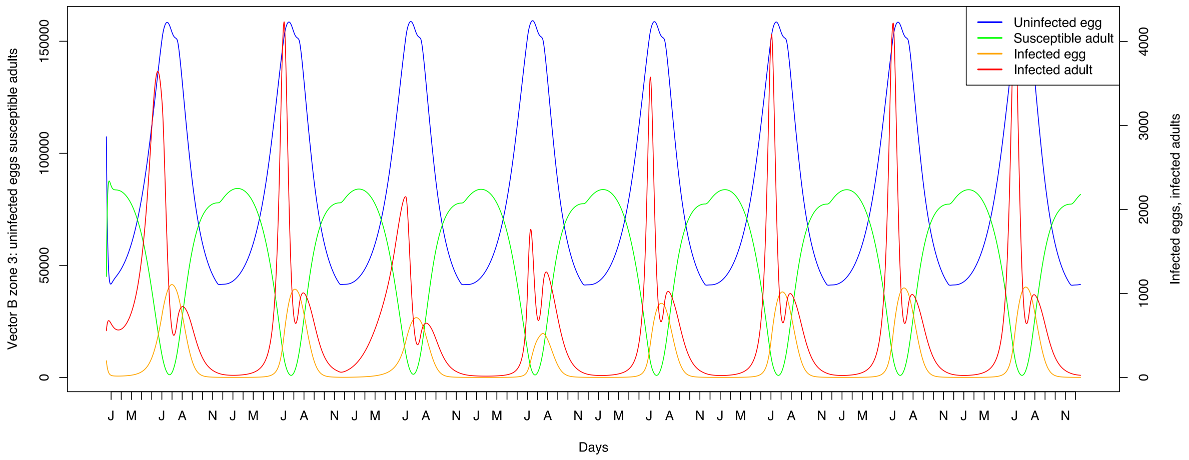

Figure 10: Standard parameters: Vector B - zone 3

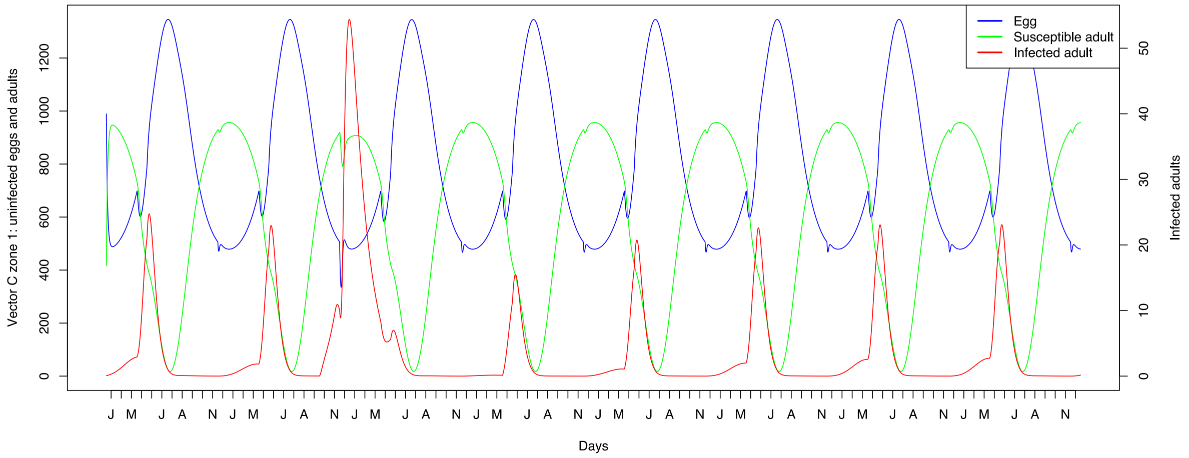

Figure 11: Standard parameters: Vector C - zone 1

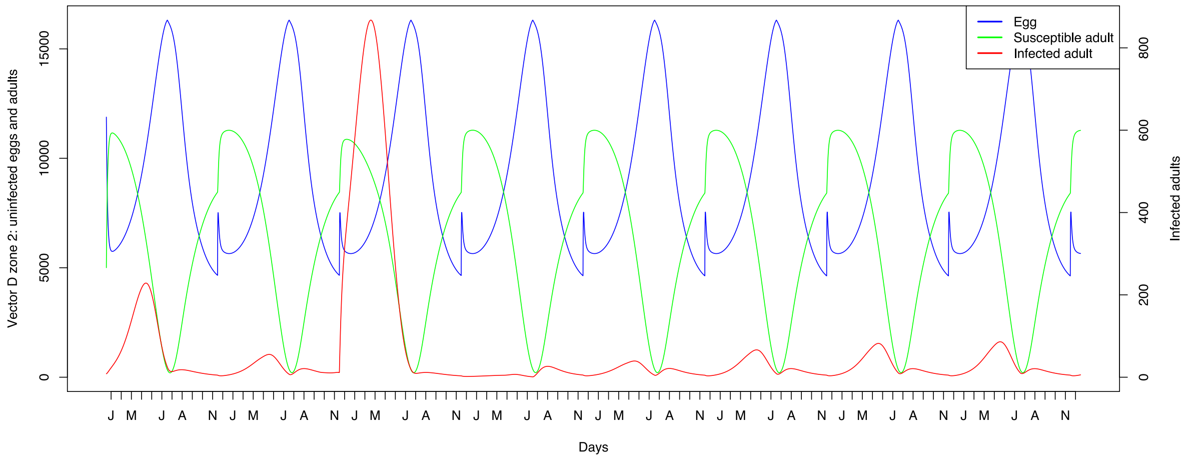

Figure 12: Standard parameters: Vector D - zone 2

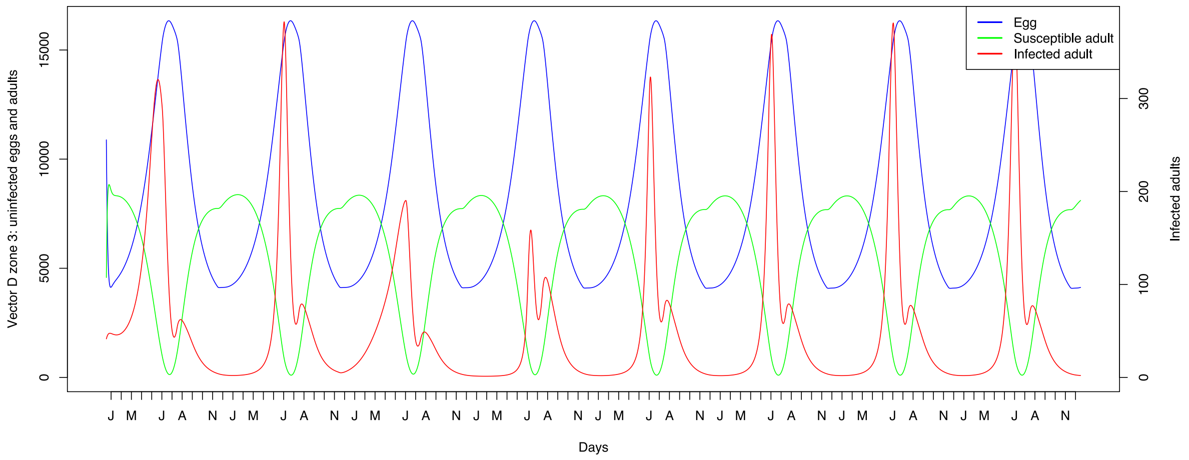

Figure 13: Standard parameters: Vector D - zone 3

Hosts

	Susceptible	Exposed	Infective	Recovered
Human	857.26	0.70	0.53	133.14
			9.40	0.13
Mammal	2010.74	0.12	16.79	403.27
			221.65	0.17

Vectors

	Clean eggs	Infected eggs	Susc. adult	Inf. adult
Vector A	123986.33	4616.18	1916.23	69.21
Vector B	201295.97	382.35	97040.54	1292.63
Vector C	897.88		470.36	2.88
Vector D	20683.98		9760.92	109.54

Seroprevalence

	Year +2	Year +4	Year +6
Human	0.209	0.147	0.132
Mammal	0.324	0.140	0.123

Figure 14: Standard parameters: Summarising output table
