# Supplementary material for: Rift Valley fever: An open-source transmission dynamics simulation model
Source: PLoS One. 2019 Jan 9;14(1):e0209929. doi: 10.1371/journal.pone.0209929 (PMC6326482; doi:10.1371/journal.pone.0209929)
Supplement: S2 Appendix — (PDF) [file pone.0209929.s002.pdf]

Listing 1: RVF.R: R interface to be used in conjunction with CppFunctions

```r
library(shiny)
library(deSolve)

#--------------- Call Cpp functions
Rcpp::sourceCpp('CppFunctions.cpp')
require(Rcpp)
options(warn=-1)

# Define the UI for the application
ui = navbarPage("Rift Valley Fever",
       tabPanel("Run simulation & select plots",
         mainPanel(fluidRow(br()),
           fluidRow(column(6, actionButton("runIt", label = h2("Run simulation")),
                    br(), br(),
                    sliderInput("year", label = "Number of years to run simulation",
                                min = 1, max = 50, value = 27, step = 1), align="center"),
                    column(6, br(), br(),
                           wellPanel(radioButtons("typeGraph", "Type of plot to be produced",
                                                  choices = c("Months" = 1, "Days elapsed" = 2), selected = 1))
                           )),
           fluidRow(column(4, numericInput("plotStart", h4("Start plotting at (day):"), value = 7201)),
                    column(4, numericInput("plotEnd", h4("Stop plotting at (day):") , value = 9720)),
                    column(4, numericInput("rngSeed", h4("RNG seed"), value = 123))),
           fluidRow(br(), hr()),
           fluidRow(column(3, h1("species"), hr(), h1("human"), hr(), h1("animal"),hr(),
                           h1("vector A"), hr(), h1("vector B"), hr(), h1("vector C"), hr(), h1("vector D", hr()),
                           align="center"),
                    column(3, h1("zone 1"), hr(),
                           h1(checkboxInput("plotH1", NULL, value = TRUE)),  hr(),
                           h1(checkboxInput("plotM1", NULL, value = TRUE)),  hr(),
                           h1(checkboxInput("plotA1", NULL, value = TRUE)),  hr(),
                           h1(checkboxInput("plotB1", NULL, value = FALSE)), hr(),
                           h1(checkboxInput("plotC1", NULL, value = TRUE)),  hr(),
                           h1(checkboxInput("plotD1", NULL, value = FALSE)), hr(),
                           align="center"),
                    column(3, h1("zone 2"), hr(),
                           h1(checkboxInput("plotH2", NULL, value = TRUE)),  hr(),
                           h1(checkboxInput("plotM2", NULL, value = TRUE)),  hr(),
                           h1(checkboxInput("plotA2", NULL, value = FALSE)), hr(),
                           h1(checkboxInput("plotB2", NULL, value = TRUE)),  hr(),
                           h1(checkboxInput("plotC2", NULL, value = FALSE)), hr(),
                           h1(checkboxInput("plotD2", NULL, value = TRUE)),  hr(),
                           align="center"),
                    column(3, h1("zone 3"), hr(),
                           h1(checkboxInput("plotH3", NULL, value = TRUE)),  hr(),
                           h1(checkboxInput("plotM3", NULL, value = TRUE)),  hr(),
                           h1(checkboxInput("plotA3", NULL, value = FALSE)), hr(),
                           h1(checkboxInput("plotB3", NULL, value = TRUE)),  hr(),
                           h1(checkboxInput("plotC3", NULL, value = FALSE)), hr(),
                           h1(checkboxInput("plotD3", NULL, value = TRUE)),
                           align="center")
             ) # fluidRow
           ) # mainPanel
         ), # tabPanel plot
       tabPanel("Initial state",
         mainPanel(
           fluidRow(
                    column(3, h3("Compartment"),
                           hr(), h3("HS", style="padding:4px;"), hr(), h3("HE", style="padding:4px;"),
                           hr(), h3("HI", style="padding:4px;"), hr(), h3("HR", style="padding:4px;"),
                           hr(), h3("MS", style="padding:4px;"), hr(), h3("ME", style="padding:4px;"),
                           hr(), h3("MI", style="padding:4px;"), hr(), h3("MR", style="padding:4px;"),
                           hr(), h3("AQ", style="padding:4px;"), hr(), h3("AP", style="padding:4px;"),
                           hr(), h3("AS", style="padding:4px;"), hr(), h3("AI", style="padding:4px;"),
                           hr(), h3("BQ", style="padding:4px;"), hr(), h3("BP", style="padding:4px;"),
                           hr(), h3("BS", style="padding:4px;"), hr(), h3("BI", style="padding:4px;"),
                           hr(), h3("CP", style="padding:4px;"), hr(), h3("CS", style="padding:4px;"),
                           hr(), h3("CI", style="padding:4px;"),
                           hr(), h3("DP", style="padding:4px;"), hr(), h3("DS", style="padding:4px;"),
                           hr(), h3("DI", style="padding:4px;"), hr(), align="center"), # column labels
                    column(3, h3("Zone 1"), hr(),
                           numericInput("HS1", NULL, value = 0), hr(), numericInput("HE1", NULL, value = 0), hr(),
                           numericInput("HI1", NULL, value = 0), hr(), numericInput("HR1", NULL, value = 0), hr(),
                           numericInput("MS1", NULL, value = 0), hr(), numericInput("ME1", NULL, value = 0), hr(),
                           numericInput("MI1", NULL, value = 0), hr(), numericInput("MR1", NULL, value = 0), hr(),
                           numericInput("AQ1", NULL, value = 100), hr(), numericInput("AP1", NULL, value = 9900), hr(),
                           numericInput("AS1", NULL, value = 0), hr(), numericInput("AI1", NULL, value = 0), hr(),
                           numericInput("BQ1", NULL, value = 0), hr(), numericInput("BP1", NULL, value = 0), hr(),
                           numericInput("BS1", NULL, value = 0), hr(), numericInput("BI1", NULL, value = 0), hr(),
                           numericInput("CP1", NULL, value = 100), hr(), numericInput("CS1", NULL, value = 0), hr(),
                           numericInput("CI1", NULL, value = 0), hr(),
                           numericInput("DP1", NULL, value = 0), hr(), numericInput("DS1", NULL, value = 0), hr(),
                           numericInput("DI1", NULL, value = 0), hr(),
                           align="center"), # column zone 1
                    column(3, h3("Zone 2"), hr(),
                           numericInput("HS2", NULL, value = 1000), hr(), numericInput("HE2", NULL, value = 0), hr(),
                           numericInput("HI2", NULL, value = 0), hr(), numericInput("HR2", NULL, value = 0), hr(),
                           numericInput("MS2", NULL, value = 2500), hr(), numericInput("ME2", NULL, value = 0), hr(),
                           numericInput("MI2", NULL, value = 0), hr(), numericInput("MR2", NULL, value = 0), hr(),
                           numericInput("AQ2", NULL, value = 0), hr(), numericInput("AP2", NULL, value = 0), hr(),
                           numericInput("AS2", NULL, value = 0), hr(), numericInput("AI2", NULL, value = 0), hr(),
```

Listing 1 (Cont.): RVF.R: R interface to be used in conjunction with CppFunctions

```
                            numericInput("BQ2", NULL, value = 0), hr(), numericInput("BP2", NULL, value = 10), hr(),
                            numericInput("BS2", NULL, value = 0), hr(), numericInput("BI2", NULL, value = 0), hr(),
                            numericInput("CP2", NULL, value = 0), hr(), numericInput("CS2", NULL, value = 0), hr(),
                            numericInput("CI2", NULL, value = 0), hr(),
                            numericInput("DP2", NULL, value = 0), hr(), numericInput("DS2", NULL, value = 1000), hr(),
                            numericInput("DI2", NULL, value = 0), hr(),
                            align="center"), # column zone 2
                     column(3, h3("Zone 3"), hr(),
                            numericInput("HS3", NULL, value = 0), hr(), numericInput("HE3", NULL, value = 0), hr(),
                            numericInput("HI3", NULL, value = 0), hr(), numericInput("HR3", NULL, value = 0), hr(),
                            numericInput("MS3", NULL, value = 0), hr(), numericInput("ME3", NULL, value = 0), hr(),
                            numericInput("MI3", NULL, value = 0), hr(), numericInput("MR3", NULL, value = 0), hr(),
                            numericInput("AQ3", NULL, value = 0), hr(), numericInput("AP3", NULL, value = 0), hr(),
                            numericInput("AS3", NULL, value = 0), hr(), numericInput("AI3", NULL, value = 0), hr(),
                            numericInput("BQ3", NULL, value = 0), hr(), numericInput("BP3", NULL, value = 10), hr(),
                            numericInput("BS3", NULL, value = 0), hr(), numericInput("BI3", NULL, value = 0), hr(),
                            numericInput("CP3", NULL, value = 0), hr(), numericInput("CS3", NULL, value = 0), hr(),
                            numericInput("CI3", NULL, value = 0), hr(),
                            numericInput("DP3", NULL, value = 1000), hr(), numericInput("DS3", NULL, value = 0), hr(),
                            numericInput("DI3", NULL, value = 0), hr(),
                            align="center") # column zone 3
              ) # fluidRow
            ) # mainPanel
          ), # tabPanel state
        tabPanel("General model information",
          mainPanel(
            fluidRow(column(12, checkboxInput("elNino", h4("El Nino flooding"), value = TRUE, width="800px"), align="left")),
            fluidRow(
                     column(4, sliderInput("d5", h4("Start"), min = 1, max = 360, value = 270), align="left"),
                     column(4, sliderInput("d6", h4("End"), min = 1, max = 360, value = 315), align="left")),
            fluidRow(column(12, checkboxInput("flood", h4("Annual flooding"), value = TRUE, width="800px"), align="left")),
            fluidRow(column(4, sliderInput("d3", h4("Start"), min = 1, max = 360, value = 91), align="left"),
                     column(4, sliderInput("d4", h4("End"), min = 1, max = 360, value = 120), align="left"),
                     column(4, numericInput("flood_prop", h4("Proportion"), value = 0.05/30))),
            fluidRow(column(12, checkboxInput("seasonHatch", h4("Seasonal effect hatching"), value = TRUE, width="800px"), align="left")),
            fluidRow(column(4, sliderInput("ds", h4("Delay"), min = -180, max = 180, value = 0), align="left"),
                     column(4, sliderInput("nPeak", h4("Number of annual peaks"), min = 1, max = 4, value = 1), align="left")),
            fluidRow(column(12, checkboxInput("wetDry", h4("Annual variation climate"), value = TRUE, width="800px"), align="left")),
            fluidRow(column(4, sliderInput("w", h4("Dry years"), min = 0, max = 5, value = 3), align="left"),
                     column(4, sliderInput("W", h4("Total years"), min = 0, max = 10, value = 7), align="left"),
                     column(4, sliderInput("mmm", h4("Minimum"), min = 0, max = 1, value = 0.1))),
            fluidRow(column(12, checkboxInput("transHumance", h4("Annual transhumance"), value = TRUE, width="800px"), align="left")),
            fluidRow(column(4, sliderInput("d1", h4("Homestead to pasture"), min = 1, max = 360, value = 181), align="left"),
                     column(4, sliderInput("d2", h4("Pasture to homestead"), min = 1, max = 360, value = 330), align="left")),
            fluidRow(column(12, checkboxInput("shearing", h4("Increased susceptibility of animals"), value = FALSE, width="800px"), align="left")),
            fluidRow(column(4, sliderInput("shearBeg", h4("Start"), min = 1, max = 360, value = 1), align="left"),
                     column(4, sliderInput("shearEnd", h4("End"), min = 1, max = 360, value = 1), align="left"),
                     column(4, numericInput("shearUp", h4("Factor"), value = 1))),
            fluidRow(column(12, checkboxInput("incrTrans", h4("Increased transmission by animals"), value = FALSE, width="800px"), align="left")),
            fluidRow(column(4, sliderInput("incrTransBeg", h4("Start"), min = 1, max = 360, value = 1), align="left"),
                     column(4, sliderInput("incrTransEnd", h4("End"), min = 1, max = 360, value = 1), align="left"),
                     column(4, numericInput("incrTransUp", h4("Factor"), value = 1))),
            fluidRow(column(12, numericInput("b_wl", h4("Infection rate wildlife"), value = 0))),
            fluidRow(column(12, numericInput("O_alt", h4("Number of bites on alternative hosts"), value = 0)))
            ) # mainPanel
          ), # tabPanel general
        tabPanel("People",
          mainPanel(
            fluidRow(column(6, numericInput("g_h", "Birth rate", value = 0.0001),
                            numericInput("m_h", "Natural mortality rate", value = 0.0001),
                            hr(),
                            numericInput("x_h", "Average length incubation period", value = 4),
                            numericInput("a_h", "Average length infective period", value = 3),
                            numericInput("d_h", "Disease-specific mortality rate", value = 0.01),
                            numericInput("r_h", "Average length immune period", value = 900),
                            hr(),
                            numericInput("l_h12", "Migration rate zones 1->2", value = 0.05),
                            numericInput("l_h13", "Migration rate zones 1->3", value = 0.0001),
                            numericInput("l_h21", "Migration rate zones 2->1", value = 0.05),
                            numericInput("l_h23", "Migration rate zones 2->3", value = 0.001),
                            numericInput("l_h31", "Migration rate zones 3->1", value = 0.005),
                            numericInput("l_h32", "Migration rate zones 3->2", value = 0.05),
                            hr(),
                            align="left"), # first column
                     column(6, numericInput("p_ha", "Infection transfer rate human -> vector A", value = 0.89),
                            numericInput("p_hb", "Infection transfer rate human -> vector B", value = 0.89),
                            numericInput("p_hc", "Infection transfer rate human -> vector C", value = 0.81),
                            numericInput("p_hd", "Infection transfer rate human -> vector D", value = 0.81),
                            hr(),
                            numericInput("f_mh1", "Contact rate human-animal zone 1", value = 2.5),
                            numericInput("f_mh2", "Contact rate human-animal zone 2", value = 2.5),
                            numericInput("f_mh3", "Contact rate human-animal zone 3", value = 2.5),
                            hr(),
                            numericInput("h_h1", "Maximum supported biting rate zone 1", value = 25),
                            numericInput("h_h2", "Maximum supported biting rate zone 2", value = 25),
                            numericInput("h_h3", "Maximum supported biting rate zone 3", value = 25),
                            hr(),
                            align="left") # second column
              ) # fluidRow
            ) # mainPanel
          ), # tabPanel human
```

Listing 1 (Cont.): RVF.R: R interface to be used in conjunction with CppFunctions

```
        tabPanel("Animal",
          mainPanel(
            fluidRow(column(6, numericInput("g_m_u", "Birth rate non-infected", value = 0.00082),
                            numericInput("p_a_i", "Proportion abortion due to RVF", value = 0.9),
                            numericInput("m_m", "Natural mortality rate", value = 0.0008),
                            hr(),
                            numericInput("k_m1", "Carrying capacity zone 1", value = 500000),
                            numericInput("k_m2", "Carrying capacity zone 2", value = 500000),
                            numericInput("k_m3", "Carrying capacity zone 3", value = 500000),
                            hr(),
                            numericInput("x_m", "Average length incubation period", value = 24/3.25),
                            numericInput("a_m", "Average length infective period", value = 20),
                            numericInput("d_m", "Disease-specific mortality rate", value = 0.05),
                            numericInput("r_m", "Average length immune period", value = 900),
                            hr(),
                            numericInput("l_m12Base", "Migration rate zones 1->2*", value = 1e-5),
                            numericInput("l_m21Base", "Migration rate zones 2->1*", value = 1e-5),
                            numericInput("l_m13", "Migration rate zones 1->3", value = 0),
                            numericInput("l_m23", "Migration rate zones 2->3", value = 0.0001),
                            numericInput("l_m31", "Migration rate zones 3->1", value = 0),
                            numericInput("l_m32", "Migration rate zones 3->2", value = 0.005), hr(),
                            align="left"), # first column,
                     column(6, numericInput("p_mh00", "Infection transfer rate animal -> human", value = 0.001),
                            numericInput("p_ma", "Infection transfer rate animal -> vector A", value = 0.89),
                            numericInput("p_mb", "Infection transfer rate animal -> vector B", value = 0.89),
                            numericInput("p_mc", "Infection transfer rate animal -> vector C", value = 0.81),
                            numericInput("p_md", "Infection transfer rate animal -> vector D", value = 0.81),
                            hr(),
                            numericInput("h_m", "Maximum supported biting rate", value = 50),
                            hr(),
                            align="left")
              ) # fluidRow
            ) # mainPanel
          ), # tabPanel animal
        tabPanel("Vector A",
          mainPanel(
            fluidRow(column(6, numericInput("g_a", "Oviposition rate", value = 10),
                     numericInput("z_a", "Proportion vertical transmission", value = 0.5),
                     numericInput("t_a", "Hatching rate", value = 0.2),
                     hr(),
                     numericInput("m_a", "Mortality rate adults", value = 1/3),
                     numericInput("m_aq1", "Mortality rate infected eggs zone 1", value = 0.00001),
                     numericInput("m_aq2", "Mortality rate infected eggs zone 2", value = 0.00001),
                     numericInput("m_aq3", "Mortality rate infected eggs zone 3", value = 0.00001),
                     numericInput("m_ap1", "Mortality rate uninfected eggs zone 1", value = 0.00001),
                     numericInput("m_ap2", "Mortality rate uninfected eggs zone 2", value = 0.00001),
                     numericInput("m_ap3", "Mortality rate uninfected eggs zone 3", value = 0.00001),
                     hr(),
                     numericInput("v_a", "Maximum biting rate", value = 0.5),
                     numericInput("e_ah", "Proportion feeding on humans", value = 0.1),
                     numericInput("p_ah", "Infection transfer rate vector A -> human", value = 0.01),
                     numericInput("e_am", "Proportion feeding on animals", value = 0.3),
                     numericInput("p_am", "Infection transfer rate vector A -> animal", value = 0.01),
                     hr(),
                     align="left"), # first column
              column(6, numericInput("k_a1", "Carrying capacity zone 1", value = 175000),
                     numericInput("k_a2", "Carrying capacity zone 2", value = 175000),
                     numericInput("k_a3", "Carrying capacity zone 3", value = 175000),
                     hr(),
                     numericInput("l_a12", "Migration rate zones 1->2", value = 0),
                     numericInput("l_a13", "Migration rate zones 1->3", value = 0),
                     numericInput("l_a21", "Migration rate zones 2->1", value = 0),
                     numericInput("l_a23", "Migration rate zones 2->3", value = 0),
                     numericInput("l_a31", "Migration rate zones 3->1", value = 0),
                     numericInput("l_a32", "Migration rate zones 3->2", value = 0),
                     hr(),
                     align="left") # second column
              ) # fluidRow
            ) # mainPanel
          ), # tabPanel Vector A
        tabPanel("Vector B",
          mainPanel(
            fluidRow(column(6, numericInput("g_b", "Oviposition rate", value = 25),
                     numericInput("z_b", "Proportion vertical transmission", value = 0.05),
                     numericInput("t_b", "Hatching rate", value = 0.2),
                     hr(),
                     numericInput("m_b", "Mortality rate adults", value = 0.1),
                     numericInput("m_bq1", "Mortality rate infected eggs zone 1", value = 0.005),
                     numericInput("m_bq2", "Mortality rate infected eggs zone 2", value = 0.005),
                     numericInput("m_bq3", "Mortality rate infected eggs zone 3", value = 0.005),
                     numericInput("m_bp1", "Mortality rate uninfected eggs zone 1", value = 0.005),
                     numericInput("m_bp2", "Mortality rate uninfected eggs zone 2", value = 0.005),
                     numericInput("m_bp3", "Mortality rate uninfected eggs zone 3", value = 0.005),
                     hr(),
                     numericInput("v_b", "Maximum biting rate", value = 0.5),
                     numericInput("e_bh", "Proportion feeding on humans", value = 0.01),
                     numericInput("p_bh", "Infection transfer rate vector B -> human", value = 0.01),
                     numericInput("e_bm", "Proportion feeding on animals", value = 0.25),
                     numericInput("p_bm", "Infection transfer rate vector B -> animal", value = 0.01),
                     hr(),
                     align="left"), # first column
              column(6, numericInput("k_b1", "Carrying capacity zone 1", value = 175000),
                     numericInput("k_b2", "Carrying capacity zone 2", value = 175000),
                     numericInput("k_b3", "Carrying capacity zone 3", value = 175000),
                     hr(),
```

Listing 1 (Cont.): RVF.R: R interface to be used in conjunction with CppFunctions

```
                    numericInput("l_b12", "Migration rate zones 1->2", value = 0),
                    numericInput("l_b13", "Migration rate zones 1->3", value = 0),
                    numericInput("l_b21", "Migration rate zones 2->1", value = 0),
                    numericInput("l_b23", "Migration rate zones 2->3", value = 0),
                    numericInput("l_b31", "Migration rate zones 3->1", value = 0),
                    numericInput("l_b32", "Migration rate zones 3->2", value = 0),
                    hr(),
                    align="left") # second column
             ) # fluidRow
           ) # mainPanel
         ), # tabPanel Vector B
       tabPanel("Vector C",
         mainPanel(
           fluidRow(column(6,
                    numericInput("g_c", "Oviposition rate", value = 25),
                    numericInput("t_c", "Hatching rate", value = 0.2),
                    hr(),
                    numericInput("m_c", "Mortality rate adults", value = 0.1),
                    numericInput("m_cp1", "Mortality rate eggs zone 1", value = 0.002),
                    numericInput("m_cp2", "Mortality rate eggs zone 2", value = 0.002),
                    numericInput("m_cp3", "Mortality rate eggs zone 3", value = 0.002),
                    hr(),
                    numericInput("v_c", "Maximum biting rate", value = 1),
                    numericInput("e_ch", "Proportion feeding on humans", value = 0.0025),
                    numericInput("p_ch", "Infection transfer rate vector C -> human", value = 0.07),
                    numericInput("e_cm", "Proportion feeding on animals", value = 0.02),
                    numericInput("p_cm", "Infection transfer rate vector C -> animal", value = 0.07),
                    hr(),
                    align="left"), # first column
             column(6, numericInput("k_c1", "Carrying capacity zone 1", value = 1750),
                    numericInput("k_c2", "Carrying capacity zone 2", value = 1750),
                    numericInput("k_c3", "Carrying capacity zone 3", value = 1750),
                    hr(),
                    numericInput("l_c12", "Migration rate zones 1->2", value = 0),
                    numericInput("l_c13", "Migration rate zones 1->3", value = 0),
                    numericInput("l_c21", "Migration rate zones 2->1", value = 0),
                    numericInput("l_c23", "Migration rate zones 2->3", value = 0),
                    numericInput("l_c31", "Migration rate zones 3->1", value = 0),
                    numericInput("l_c32", "Migration rate zones 3->2", value = 0),
                    hr(),
                    align="left") # second column
             ) # fluidRow
           ) # mainPanel
         ), # tabPanel Vector C
       tabPanel("Vector D",
         mainPanel(
           fluidRow(column(6, numericInput("g_d", "Oviposition rate", value = 25),
                    numericInput("t_d", "Hatching rate", value = 0.2),
                    hr(),
                    numericInput("m_d", "Mortality rate adults", value = 0.1),
                    numericInput("m_dp1", "Mortality rate eggs zone 1", value = 0.002),
                    numericInput("m_dp2", "Mortality rate eggs zone 2", value = 0.002),
                    numericInput("m_dp3", "Mortality rate eggs zone 3", value = 0.002),
                    hr(),
                    numericInput("v_d", "Maximum biting rate", value = 1),
                    numericInput("e_dh", "Proportion feeding on humans", value = 0.005),
                    numericInput("p_dh", "Infection transfer rate vector D -> human", value = 0.07),
                    numericInput("e_dm", "Proportion feeding on animals", value = 0.12),
                    numericInput("p_dm", "Infection transfer rate vector D -> animal", value = 0.07),
                    hr(),
                    align="left"), # first column
             column(6, numericInput("k_d1", "Carrying capacity zone 1", value = 17500),
                    numericInput("k_d2", "Carrying capacity zone 2", value = 17500),
                    numericInput("k_d3", "Carrying capacity zone 3", value = 17500),
                    hr(),
                    numericInput("l_d12", "Migration rate zones 1->2", value = 0),
                    numericInput("l_d13", "Migration rate zones 1->3", value = 0),
                    numericInput("l_d21", "Migration rate zones 2->1", value = 0),
                    numericInput("l_d23", "Migration rate zones 2->3", value = 0),
                    numericInput("l_d31", "Migration rate zones 3->1", value = 0),
                    numericInput("l_d32", "Migration rate zones 3->2", value = 0),
                    hr(),
                    align="left") # second column
             ) # fluidRow
           ) # mainPanel
         ), # tabPanel Vector D
       tabPanel("Summary tables",
         mainPanel(
           fluidRow(column(12, wellPanel(h4("Hosts"), tableOutput("summary"))), # column
                    column(12, wellPanel(h4("Vectors"), tableOutput("summary2"))), # column
                    column(12, wellPanel(h4("Seroprevalence"), tableOutput("summary3"))) # column
             ) # fluidRow
           ) # mainPanel
         ) # tabPanel Summary
  ) # navbarPage


# Define the server function for the application
server = function(input, output) {
observe({ if(input$runIt > 0)
{
  start = Sys.time(); print(start)
  isolate({
    # Which plots to be generated
    ps = input$plotStart; ps2 = ps - 1; pe = input$plotEnd
```

Listing 1 (Cont.): RVF.R: R interface to be used in conjunction with CppFunctions

```r
    if(input$rngSeed>0) set.seed(input$rngSeed); typeGraph = input$typeGraph
    plotH1 = input$plotH1; plotM1 = input$plotM1; plotA1 = input$plotA1; plotB1 = input$plotB1; plotC1 = input$plotC1; plotD1 = input$plotD1
    plotH2 = input$plotH2; plotM2 = input$plotM2; plotA2 = input$plotA2; plotB2 = input$plotB2; plotC2 = input$plotC2; plotD2 = input$plotD2
    plotH3 = input$plotH3; plotM3 = input$plotM3; plotA3 = input$plotA3; plotB3 = input$plotB3; plotC3 = input$plotC3; plotD3 = input$plotD3

    # Initialization of all the parameters and the initial values of the compartments per species
    aa = as.double(input$HS1); ab = as.double(input$HE1); ac = as.double(input$HI1); ad = as.double(input$HR1)
    ae = as.double(input$HS2); af = as.double(input$HE2); ag = as.double(input$HI2); ah = as.double(input$HR2)
    ai = as.double(input$HS3); aj = as.double(input$HE3); ak = as.double(input$HI3); al = as.double(input$HR3)
    am = as.double(input$MS1); an = as.double(input$ME1); ao = as.double(input$MI1); ap = as.double(input$MR1)
    aq = as.double(input$MS2); ar = as.double(input$ME2); as = as.double(input$MI2); at = as.double(input$MR2)
    au = as.double(input$MS3); av = as.double(input$ME3); aw = as.double(input$MI3); ax = as.double(input$MR3)
    ay = as.double(input$AQ1); az = as.double(input$AP1); ba = as.double(input$AS1); bb = as.double(input$AI1)
    bc = as.double(input$AQ2); bd = as.double(input$AP2); be = as.double(input$AS2); bf = as.double(input$AI2)
    bg = as.double(input$AQ3); bh = as.double(input$AP3); bi = as.double(input$AS3); bj = as.double(input$AI3)
    bk = as.double(input$BQ1); bl = as.double(input$BP1); bm = as.double(input$BS1); bn = as.double(input$BI1)
    bo = as.double(input$BQ2); bp = as.double(input$BP2); bq = as.double(input$BS2); br = as.double(input$BI2)
    bs = as.double(input$BQ3); bt = as.double(input$BP3); bu = as.double(input$BS3); bv = as.double(input$BI3)
    bw = as.double(input$CP1); bx = as.double(input$CS1); by = as.double(input$CI1)
    bz = as.double(input$CP2); ca = as.double(input$CS2); cb = as.double(input$CI2)
    cc = as.double(input$CP3); cd = as.double(input$CS3); ce = as.double(input$CI3)
    cf = as.double(input$DP1); cg = as.double(input$DS1); ch = as.double(input$DI1)
    ci = as.double(input$DP2); cj = as.double(input$DS2); ck = as.double(input$DI2)
    cl = as.double(input$DP3); cm = as.double(input$DS3); cn = as.double(input$DI3)
    # set ON/OFF switches and general parameters
    elNino = input$elNino; d5 = input$d5; d6 = input$d6
    flood = input$flood; d3 = input$d3; d4 = input$d4; flood_prop = input$flood_prop
    seasonHatch = input$seasonHatch; ds = input$ds; nPeak = 180/input$nPeak
    wetDry = input$wetDry; w = input$w;  W = input$W; mmm = input$mmm
    transHumance = input$transHumance; d1 = input$d1; d2 = input$d2
    shearing = input$shearing; shearBeg = input$shearBeg; shearEnd = input$shearEnd; shearUp = input$shearUp
    incrTrans = input$incrTrans; incrTransBeg = input$incrTransBeg; incrTransEnd = input$incrTransEnd; incrTransUp = input$incrTransUp
    b_wl = input$b_wl; O_alt = input$O_alt; year = input$year
    if(ps < 1 | ps > year*360) ps = 1
    if(pe < ps | pe > year*360) pe = year*360
    ps = 1 + ps/0.1; pe = 1 + pe/0.1

    # Population parameter initialisations
    #---------------- Parameter initialisation human equations
    g_h = input$g_h; m_h = input$m_h; x_h = 1/input$x_h; a_h = (1-input$d_h)/input$a_h; d_h = input$d_h/input$a_h; p_mh00 = input$p_mh00
    f_mh1 = input$f_mh1; f_mh2 = input$f_mh2; f_mh3 = input$f_mh3; h_h1 = input$h_h1; h_h2 = input$h_h2; h_h3 = input$h_h3
    p_ha = input$p_ha; p_hb = input$p_hb; p_hc = input$p_hc; p_hd = input$p_hd; r_h = 1/input$r_h
    l_h12 = input$l_h12; l_h13 = input$l_h13; l_h21 = input$l_h21; l_h23 = input$l_h23; l_h31 = input$l_h31; l_h32 = input$l_h32
    #---------------- Parameter initialisation animal host equations
    g_m_u = input$g_m_u; g_m_i = (1-input$p_a_i)*g_m_u; m_m = input$m_m; x_m = input$x_m; a_m = (1-input$d_m)/input$a_m
    d_m = input$d_m/input$a_m; h_m = input$h_m
    p_ma = input$p_ma; p_mb = input$p_mb; p_mc = input$p_mc; p_md = input$p_md
    r_m = 1/input$r_m; k_m1 = input$k_m1; k_m2 = input$k_m2; k_m3 = input$k_m3
    l_m13 = input$l_m13; l_m23 = input$l_m23; l_m31 = input$l_m31; l_m32 = input$l_m32
    l_m12Base = input$l_m12Base; l_m21Base = input$l_m21Base
    #---------------- Parameter initialisation vector A equations
    g_a = input$g_a; z_a = input$z_a; m_a = input$m_a; t_a = input$t_a; v_a = input$v_a; e_ah = input$e_ah
    e_am = input$e_am; p_ah = input$p_ah; p_am = input$p_am
    k_a1 = input$k_a1; m_aq1 = input$m_aq1; m_ap1 = input$m_ap1; k_a2 = input$k_a2; m_aq2 = input$m_aq2; m_ap2 = input$m_ap2
    k_a3 = input$k_a3; m_aq3 = input$m_aq3; m_ap3 = input$m_ap3
    l_a12 = input$l_a12; l_a13 = input$l_a13; l_a21 = input$l_a21; l_a23 = input$l_a23; l_a31 = input$l_a31; l_a32 = input$l_a32
    #---------------- Parameter initialisation vector B equations
    g_b = input$g_b; z_b = input$z_b; m_b = input$m_b; t_b = input$t_b;  v_b = input$v_b
    e_bh = input$e_bh; e_bm = input$e_bm; p_bh = input$p_bh; p_bm = input$p_bm
    k_b1 = input$k_b1; m_bq1 = input$m_bq1; m_bp1 = input$m_bp1; k_b2 = input$k_b2; m_bq2 = input$m_bq2; m_bp2 = input$m_bp2
    k_b3 = input$k_b3; m_bq3 = input$m_bq3; m_bp3 = input$m_bp3
    l_b12 = input$l_b12; l_b13 = input$l_b13; l_b21 = input$l_b21; l_b23 = input$l_b23; l_b31 = input$l_b31; l_b32 = input$l_b32
    #---------------- Parameter initialisation vector C equations
    g_c = input$g_c; m_c = input$m_c; t_c = input$t_c;  v_c = input$v_c
    e_ch = input$e_ch; e_cm = input$e_cm; p_ch = input$p_ch; p_cm = input$p_cm
    k_c1 = input$k_c1; m_cp1 = input$m_cp1; k_c2 = input$k_c2; m_cp2 = input$m_cp2; k_c3 = input$k_c3; m_cp3 = input$m_cp3
    l_c12 = input$l_c12; l_c13 = input$l_c13; l_c21 = input$l_c21; l_c23 = input$l_c23; l_c31 = input$l_c31; l_c32 = input$l_c32
    #---------------- Parameter initialisation vector D equations
    g_d = input$g_d; m_d = input$m_d; t_d = input$t_d;  v_d = input$v_d
    e_dh = input$e_dh; e_dm = input$e_dm; p_dh = input$p_dh; p_dm = input$p_dm
    k_d1 = input$k_d1; m_dp1 = input$m_dp1; k_d2 = input$k_d2; m_dp2 = input$m_dp2; k_d3 = input$k_d3; m_dp3 = input$m_dp3
    l_d12 = input$l_d12; l_d13 = input$l_d13; l_d21 = input$l_d21; l_d23 = input$l_d23; l_d31 = input$l_d31; l_d32 = input$l_d32
  })

  # General initialisations
  #---------------- Initialisation of maximum rate allowed in ODE to avoid negative values
  #---------------- (if problems are encountered lower max_rate to 9)
  max_rate = 10
  #---------------- Timeframe
  times = round(seq(0, year*360, 0.1), 1); ntimes = length(times)
  #---------------- Wet and dry years
  dry2 = runif(year+1);  c0 = w/W
  #---------------- Initial state
  state = c(HS1 = aa, HE1 = ab, HI1 = ac, HR1 = ad, HS2 = ae, HE2 = af, HI2 = ag, HR2 = ah, HS3 = ai, HE3 = aj, HI3 = ak, HR3 = al,
            MS1 = am, ME1 = an, MI1 = ao, MR1 = ap, MS2 = aq, ME2 = ar, MI2 = as, MR2 = at, MS3 = au, ME3 = av, MI3 = aw, MR3 = ax,
            AQ1 = ay, AP1 = az, AS1 = ba, AI1 = bb, AQ2 = bc, AP2 = bd, AS2 = be, AI2 = bf, AQ3 = bg, AP3 = bh, AS3 = bi, AI3 = bj,
            BQ1 = bk, BP1 = bl, BS1 = bm, BI1 = bn, BQ2 = bo, BP2 = bp, BS2 = bq, BI2 = br, BQ3 = bs, BP3 = bt, BS3 = bu, BI3 = bv,
            CP1 = bw, CS1 = bx, CI1 = by,           CP2 = bz, CS2 = ca, CI2 = cb,           CP3 = cc, CS3 = cd, CI3 = ce,
            DP1 = cf, DS1 = cg, DI1 = ch,           DP2 = ci, DS2 = cj, DI2 = ck,           DP3 = cl, DS3 = cm, DI3 = cn)
  #---------------- Parameters to be passed to ODE function
```

Listing 1 (Cont.): RVF.R: R interface to be used in conjunction with CppFunctions

```r
parameters = c(max_rate, flood_prop, b_wl, O_alt, d1, d2, d3, d4, d5, d6, year, c0, mmm, ds, nPeak, seasonHatch,
               g_h, m_h, x_h, a_h, d_h, p_mh00, f_mh1, f_mh2, f_mh3, h_h1, h_h2, h_h3,
               p_ha, p_hb, p_hc, p_hd, r_h, l_h12, l_h13, l_h21, l_h23, l_h31, l_h32,
               g_m_u, g_m_i, m_m, x_m, a_m, d_m, h_m, p_ma, p_mb, p_mc, p_md, r_m, k_m1, k_m2, k_m3,
               l_m13, l_m23, l_m31, l_m32,
               g_a, z_a, m_a, v_a, e_ah, e_am, p_ah, p_am, k_a1, k_a2, k_a3,
               m_aq1, m_aq2, m_aq3, m_ap1, m_ap2, m_ap3, l_a12, l_a13, l_a21, l_a23, l_a31, l_a32,
               g_b, z_b, m_b, t_b, v_b, e_bh, e_bm, p_bh, p_bm, k_b1, k_b2, k_b3,
               m_bq1, m_bq2, m_bq3, m_bp1, m_bp2, m_bp3, l_b12, l_b13, l_b21, l_b23, l_b31, l_b32,
               g_c, m_c, t_c, v_c, e_ch, e_cm, p_ch, p_cm, k_c1, k_c2, k_c3, m_cp1, m_cp2, m_cp3,
               l_c12, l_c13, l_c21, l_c23, l_c31, l_c32,
               g_d, m_d, t_d, v_d, e_dh, e_dm, p_dh, p_dm, k_d1, k_d2, k_d3, m_dp1, m_dp2, m_dp3,
               l_d12, l_d13, l_d21, l_d23, l_d31, l_d32, shearing, shearBeg, shearEnd, shearUp,
               wetDry, flood, elNino, transHumance, t_a,l_m21Base,l_m12Base,
               incrTrans, incrTransBeg, incrTransEnd, incrTransUp, dry2)

#--------------- ODE function
rvfODE = function(t,  state, parameters)
{
  with(as.list(c(state,parameters)),
       {ODE(t,  state, param=parameters)})
}

out = as.data.frame(ode(y=state, times=times, func=rvfODE, parms=parameters, method="rk4"))
print(Sys.time() - start)

# User selected x-axis of plots to be 'months'
if(typeGraph == 1){
  if(plotH1) drawPlot4((times[ps:pe]), out$HS1[ps:pe], out$HR1[ps:pe], out$HE1[ps:pe], out$HI1[ps:pe],
                       "Human zone 1", "SUSCEPTIBLE, RECOVERED", "EXPOSED, INFECTED", c("Susceptible","Recovered","Exposed","Infected"), ps, pe, year)
  if(plotH2) drawPlot4(times[ps:pe], out$HS2[ps:pe], out$HR2[ps:pe], out$HE2[ps:pe], out$HI2[ps:pe],
                       "Human zone 2", "SUSCEPTIBLE, RECOVERED", "EXPOSED, INFECTED", c("Susceptible","Recovered","Exposed","Infected"), ps, pe, year)
  if(plotH3) drawPlot4(times[ps:pe], out$HS3[ps:pe], out$HR3[ps:pe], out$HE3[ps:pe], out$HI3[ps:pe],
                       "Human zone 3", "SUSCEPTIBLE, RECOVERED", "EXPOSED, INFECTED", c("Susceptible","Recovered","Exposed","Infected"), ps, pe, year)
  if(plotM1) drawPlot4(times[ps:pe], out$MS1[ps:pe], out$MR1[ps:pe], out$ME1[ps:pe], out$MI1[ps:pe],
                       "Animals zone 1", "SUSCEPTIBLE, RECOVERED", "EXPOSED, INFECTED", c("Susceptible","Recovered","Exposed","Infected"), ps, pe, year)
  if(plotM2) drawPlot4(times[ps:pe], out$MS2[ps:pe], out$MR2[ps:pe], out$ME2[ps:pe], out$MI2[ps:pe],
                       "Animals zone 2", "SUSCEPTIBLE, RECOVERED", "EXPOSED, INFECTED", c("Susceptible","Recovered","Exposed","Infected"), ps, pe, year)
  if(plotM3) drawPlot4(times[ps:pe], out$MS3[ps:pe], out$MR3[ps:pe], out$ME3[ps:pe], out$MI3[ps:pe],
                       "Animals zone 3", "SUSCEPTIBLE, RECOVERED", "EXPOSED, INFECTED", c("Susceptible","Recovered","Exposed","Infected"), ps, pe, year)
  if(plotA1) drawPlot4(times[ps:pe], out$AP1[ps:pe], out$AS1[ps:pe], out$AQ1[ps:pe], out$AI1[ps:pe],
                       "Vector A zone 1", "UNINFECTED EGGS, SUSCEPTIBLE ADULTS", "INFECTED EGGS, INFECTED ADULTS",
                       c("Uninfected egg","Susceptible adult","Infected egg","Infected adult"), ps, pe, year)
  if(plotA2) drawPlot4(times[ps:pe], out$AP2[ps:pe], out$AS2[ps:pe], out$AQ2[ps:pe], out$AI2[ps:pe],
                       "Vector A zone 2", "UNINFECTED EGGS, SUSCEPTIBLE ADULTS", "INFECTED EGGS, INFECTED ADULTS",
                       c("Uninfected egg","Susceptible adult","Infected egg","Infected adult"), ps, pe, year)
  if(plotA3) drawPlot4(times[ps:pe], out$AP3[ps:pe], out$AS3[ps:pe], out$AQ3[ps:pe], out$AI3[ps:pe],
                       "Vector A zone 3", "UNINFECTED EGGS, SUSCEPTIBLE ADULTS", "INFECTED EGGS, INFECTED ADULTS",
                       c("Uninfected egg","Susceptible adult","Infected egg","Infected adult"), ps, pe, year)
  if(plotB1) drawPlot4(times[ps:pe], out$BP1[ps:pe], out$BS1[ps:pe], out$BQ1[ps:pe], out$BI1[ps:pe],
                       "Vector B zone 1", "UNINFECTED EGGS, SUSCEPTIBLE ADULTS", "INFECTED EGGS, INFECTED ADULTS",
                       c("Uninfected egg","Susceptible adult","Infected egg","Infected adult"), ps, pe, year)
  if(plotB2) drawPlot4(times[ps:pe], out$BP2[ps:pe], out$BS2[ps:pe], out$BQ2[ps:pe], out$BI2[ps:pe],
                       "Vector B zone 2", "UNINFECTED EGGS, SUSCEPTIBLE ADULTS", "INFECTED EGGS, INFECTED ADULTS",
                       c("Uninfected egg","Susceptible adult","Infected egg","Infected adult"), ps, pe, year)
  if(plotB3) drawPlot4(times[ps:pe], out$BP3[ps:pe], out$BS3[ps:pe], out$BQ3[ps:pe], out$BI3[ps:pe],
                       "Vector B zone 3", "UNINFECTED EGGS, SUSCEPTIBLE ADULTS", "INFECTED EGGS, INFECTED ADULTS",
                       c("Uninfected egg","Susceptible adult","Infected egg","Infected adult"), ps, pe, year)
  if(plotC1) drawPlot3(times[ps:pe], out$CP1[ps:pe], out$CS1[ps:pe], out$CI1[ps:pe],
                       "Vector C zone 1", "UNINFECTED EGGS, UNINFECTED ADULTS", "INFECTED ADULTS", c("Egg", "Susceptible adult","Infected adult"), ps, pe, year)
  if(plotC2) drawPlot3(times[ps:pe], out$CP2[ps:pe], out$CS2[ps:pe], out$CI2[ps:pe],
                       "Vector C zone 2", "UNINFECTED EGGS, UNINFECTED ADULTS", "INFECTED ADULTS", c("Egg", "Susceptible adult","Infected adult"), ps, pe, year)
  if(plotC3) drawPlot3(times[ps:pe], out$CP3[ps:pe], out$CS3[ps:pe], out$CI3[ps:pe],
                       "Vector C zone 3", "UNINFECTED EGGS, UNINFECTED ADULTS", "INFECTED ADULTS", c("Egg", "Susceptible adult","Infected adult"), ps, pe, year)
  if(plotD1) drawPlot3(times[ps:pe], out$DP1[ps:pe], out$DS1[ps:pe], out$DI1[ps:pe],
                       "Vector D zone 1", "UNINFECTED EGGS, UNINFECTED ADULTS", "INFECTED ADULTS", c("Egg", "Susceptible adult","Infected adult"), ps, pe, year)
  if(plotD2) drawPlot3(times[ps:pe], out$DP2[ps:pe], out$DS2[ps:pe], out$DI2[ps:pe],
                       "Vector D zone 2", "UNINFECTED EGGS, UNINFECTED ADULTS", "INFECTED ADULTS", c("Egg", "Susceptible adult","Infected adult"), ps, pe, year)
  if(plotD3) drawPlot3(times[ps:pe], out$DP3[ps:pe], out$DS3[ps:pe], out$DI3[ps:pe],
                       "Vector D zone 3", "UNINFECTED EGGS, UNINFECTED ADULTS", "INFECTED ADULTS", c("Egg", "Susceptible adult","Infected adult"), ps, pe, year)
}

# user selected x-axis of plots to be 'days lapsed'
if(typeGraph == 2){
  if(plotH1) drawPlot4b((times[ps:pe] - ps2), out$HS1[ps:pe], out$HR1[ps:pe], out$HE1[ps:pe], out$HI1[ps:pe],
                       "Human zone 1", "SUSCEPTIBLE, RECOVERED", "EXPOSED, INFECTED", c("Susceptible","Recovered","Exposed","Infected"), ps, pe, year)
  if(plotH2) drawPlot4b((times[ps:pe] - ps2), out$HS2[ps:pe], out$HR2[ps:pe], out$HE2[ps:pe], out$HI2[ps:pe],
                       "Human zone 2", "SUSCEPTIBLE, RECOVERED", "EXPOSED, INFECTED", c("Susceptible","Recovered","Exposed","Infected"), ps, pe, year)
  if(plotH3) drawPlot4b((times[ps:pe] - ps2), out$HS3[ps:pe], out$HR3[ps:pe], out$HE3[ps:pe], out$HI3[ps:pe],
                       "Human zone 3", "SUSCEPTIBLE, RECOVERED", "EXPOSED, INFECTED", c("Susceptible","Recovered","Exposed","Infected"), ps, pe, year)
```

```
    if(plotM1) drawPlot4b((times[ps:pe] - ps2), out$MS1[ps:pe], out$MR1[ps:pe], out$ME1[ps:pe], out$MI1[ps:pe],
                          "Animals zone 1", "SUSCEPTIBLE, RECOVERED", "EXPOSED, INFECTED", c("Susceptible","Recovered","Exposed","
                               Infected"), ps, pe, year)
    if(plotM2) drawPlot4b((times[ps:pe] - ps2), out$MS2[ps:pe], out$MR2[ps:pe], out$ME2[ps:pe], out$MI2[ps:pe],
                          "Animals zone 2", "SUSCEPTIBLE, RECOVERED", "EXPOSED, INFECTED", c("Susceptible","Recovered","Exposed","
                               Infected"), ps, pe, year)
    if(plotM2) drawPlot4b((times[ps:pe] - ps2), out$MS3[ps:pe], out$MR3[ps:pe], out$ME3[ps:pe], out$MI3[ps:pe],
                          "Animals zone 3", "SUSCEPTIBLE, RECOVERED", "EXPOSED, INFECTED", c("Susceptible","Recovered","Exposed","
                               Infected"), ps, pe, year)
    if(plotA1) drawPlot4b((times[ps:pe] - ps2), out$AP1[ps:pe], out$AS1[ps:pe], out$AQ1[ps:pe], out$AI1[ps:pe],
                          "Vector A zone 1", "UNINFECTED EGGS, SUSCEPTIBLE ADULTS", "INFECTED EGGS, INFECTED ADULTS",
                          c("Uninfected egg","Susceptible adult","Infected egg","Infected adult"), ps, pe, year)
    if(plotA2) drawPlot4b((times[ps:pe] - ps2), out$AP2[ps:pe], out$AS2[ps:pe], out$AQ2[ps:pe], out$AI2[ps:pe],
                          "Vector A zone 2", "UNINFECTED EGGS, SUSCEPTIBLE ADULTS", "INFECTED EGGS, INFECTED ADULTS",
                          c("Uninfected egg","Susceptible adult","Infected egg","Infected adult"), ps, pe, year)
    if(plotA3) drawPlot4b((times[ps:pe] - ps2), out$AP3[ps:pe], out$AS3[ps:pe], out$AQ3[ps:pe], out$AI3[ps:pe],
                          "Vector A zone 3", "UNINFECTED EGGS, SUSCEPTIBLE ADULTS", "INFECTED EGGS, INFECTED ADULTS",
                          c("Uninfected egg","Susceptible adult","Infected egg","Infected adult"), ps, pe, year)
    if(plotB1) drawPlot4b((times[ps:pe] - ps2), out$BP1[ps:pe], out$BS1[ps:pe], out$BQ1[ps:pe], out$BI1[ps:pe],
                          "Vector B zone 1", "UNINFECTED EGGS, SUSCEPTIBLE ADULTS", "INFECTED EGGS, INFECTED ADULTS",
                          c("Uninfected egg","Susceptible adult","Infected egg","Infected adult"), ps, pe, year)
    if(plotB2) drawPlot4b((times[ps:pe] - ps2), out$BP2[ps:pe], out$BS2[ps:pe], out$BQ2[ps:pe], out$BI2[ps:pe],
                          "Vector B zone 2", "UNINFECTED EGGS, SUSCEPTIBLE ADULTS", "INFECTED EGGS, INFECTED ADULTS",
                          c("Uninfected egg","Susceptible adult","Infected egg","Infected adult"), ps, pe, year)
    if(plotB3) drawPlot4b((times[ps:pe] - ps2), out$BP3[ps:pe], out$BS3[ps:pe], out$BQ3[ps:pe], out$BI3[ps:pe],
                          "Vector B zone 3", "UNINFECTED EGGS, SUSCEPTIBLE ADULTS", "INFECTED EGGS, INFECTED ADULTS",
                          c("Uninfected egg","Susceptible adult","Infected egg","Infected adult"), ps, pe, year)
    if(plotC1) drawPlot3b((times[ps:pe] - ps2), out$CP1[ps:pe], out$CS1[ps:pe], out$CI1[ps:pe],
                          "Vector C zone 1", "UNINFECTED EGGS, UNINFECTED ADULTS", "INFECTED ADULTS", c("Egg", "Susceptible adult","
                               Infected adult"), ps, pe, year)
    if(plotC2) drawPlot3b((times[ps:pe] - ps2), out$CP2[ps:pe], out$CS2[ps:pe], out$CI2[ps:pe],
                          "Vector C zone 2", "UNINFECTED EGGS, UNINFECTED ADULTS", "INFECTED ADULTS", c("Egg", "Susceptible adult","
                               Infected adult"), ps, pe, year)
    if(plotC3) drawPlot3b((times[ps:pe] - ps2), out$CP3[ps:pe], out$CS3[ps:pe], out$CI3[ps:pe],
                          "Vector C zone 3", "UNINFECTED EGGS, UNINFECTED ADULTS", "INFECTED ADULTS", c("Egg", "Susceptible adult","
                               Infected adult"), ps, pe, year)
    if(plotD1) drawPlot3b((times[ps:pe] - ps2), out$DP1[ps:pe], out$DS1[ps:pe], out$DI1[ps:pe],
                          "Vector D zone 1", "UNINFECTED EGGS, UNINFECTED ADULTS", "INFECTED ADULTS", c("Egg", "Susceptible adult","
                               Infected adult"), ps, pe, year)
    if(plotD2) drawPlot3b((times[ps:pe] - ps2), out$DP2[ps:pe], out$DS2[ps:pe], out$DI2[ps:pe],
                          "Vector D zone 2", "UNINFECTED EGGS, UNINFECTED ADULTS", "INFECTED ADULTS", c("Egg", "Susceptible adult","
                               Infected adult"), ps, pe, year)
    if(plotD3) drawPlot3b((times[ps:pe] - ps2), out$DP3[ps:pe], out$DS3[ps:pe], out$DI3[ps:pe],
                          "Vector D zone 3", "UNINFECTED EGGS, UNINFECTED ADULTS", "INFECTED ADULTS", c("Egg", "Susceptible adult","
                               Infected adult"), ps, pe, year)
  }

  ## Generate summarising tables
  ### Hosts
  summ = as.data.frame(array(NA, c(4,4), dimnames = list(c("Human", "", "Mammal", " "), c("Susceptible", "Exposed", "Infective", "
       Recovered"))))
  summ[1,1] = mean(out$HS1) + mean(out$HS2) + mean(out$HS3)
  summ[1,2] = mean(out$HE1) + mean(out$HE2) + mean(out$HE3)
  summ[1,3] = mean(out$HI1) + mean(out$HI2) + mean(out$HI3)
  summ[1,4] = mean(out$HR1) + mean(out$HR2) + mean(out$HR3)
  summ[2,3] = max(out$HI1 + out$HI2 + out$HI3)
  summ[2,4] = mean(out$HR1+out$HR2+out$HR3)/mean(out$HS1+out$HS2+out$HS3+out$HE1+out$HE2+out$HE3+out$HI1+out$HI2+out$HI3+out$HR1+out
       $HR2+out$HR3)
  summ[3,1] = mean(out$MS1) + mean(out$MS2) + mean(out$MS3)
  summ[3,2] = mean(out$ME1) + mean(out$ME2) + mean(out$ME3)
  summ[3,3] = mean(out$MI1) + mean(out$MI2) + mean(out$MI3)
  summ[3,4] = mean(out$MR1) + mean(out$MR2) + mean(out$MR3)
  summ[4,3] = max(out$MI1 + out$MI2 + out$MI3)
  summ[4,4] = mean(out$MR1+out$MR2+out$MR3)/mean(out$MS1+out$MS2+out$MS3+out$ME1+out$ME2+out$ME3+out$MI1+out$MI2+out$MI3+out$MR1+out
       $MR2+out$MR3)
  output$summary = renderTable(summ, digits = 2, rownames = T, na = "")
  ### Vectors
  summ2 = as.data.frame(array(NA, c(4,4), dimnames = list(c("Vector A", "Vector B", "Vector C", "Vector D"), c("Clean eggs", "
       Infected eggs", "Susc. adult", "Inf. adult"))))
  summ2[1,1] = mean(out$AP1) + mean(out$AP2) + mean(out$AP3)
  summ2[1,2] = mean(out$AQ1) + mean(out$AQ2) + mean(out$AQ3)
  summ2[1,3] = mean(out$AS1) + mean(out$AS2) + mean(out$AS3)
  summ2[1,4] = mean(out$AI1) + mean(out$AI2) + mean(out$AI3)
  summ2[2,1] = mean(out$BP1) + mean(out$BP2) + mean(out$BP3)
  summ2[2,2] = mean(out$BQ1) + mean(out$BQ2) + mean(out$BQ3)
  summ2[2,3] = mean(out$BS1) + mean(out$BS2) + mean(out$BS3)
  summ2[2,4] = mean(out$BI1) + mean(out$BI2) + mean(out$BI3)
  summ2[3,1] = mean(out$CP1) + mean(out$CP2) + mean(out$CP3)
  summ2[3,3] = mean(out$CS1) + mean(out$CS2) + mean(out$CS3)
  summ2[3,4] = mean(out$CI1) + mean(out$CI2) + mean(out$CI3)
  summ2[4,1] = mean(out$DP1) + mean(out$DP2) + mean(out$DP3)
  summ2[4,3] = mean(out$DS1) + mean(out$DS2) + mean(out$DS3)
  summ2[4,4] = mean(out$DI1) + mean(out$DI2) + mean(out$DI3)
  output$summary2 = renderTable(summ2, digits = 2, rownames = T, na = "")
  if(year>=7){
    summ3 = as.data.frame(array(NA, c(2,3), dimnames = list(c("Human", "Mammal"), c("Year +2", "Year +4", "Year +6"))))
    ss = ((year-7) %/% 10) * 36000; s3 = ss + 2*3600+1; e3 = ss + 3*3600; s5 = ss + 4*3600+1; e5 = ss + 5*3600; s7 = ss + 6*3600+1;
         e7 = ss + 7*3600
    summ3[1,1] = mean(out$HR1[s3:e3]+out$HR2[s3:e3]+out$HR3[s3:e3])/mean(out$HS1[s3:e3]+out$HS2[s3:e3]+out$HS3[s3:e3]+out$HE1[s3:e3
         ]+out$HE2[s3:e3]+out$HE3[s3:e3]+out$HI1[s3:e3]+out$HI2[s3:e3]+out$HI3[s3:e3]+out$HR1[s3:e3]+out$HR2[s3:e3]+out$HR3[s3:e3])
    summ3[1,2] = mean(out$HR1[s5:e5]+out$HR2[s5:e5]+out$HR3[s5:e5])/mean(out$HS1[s5:e5]+out$HS2[s5:e5]+out$HS3[s5:e5]+out$HE1[s5:e5
         ]+out$HE2[s5:e5]+out$HE3[s5:e5]+out$HI1[s5:e5]+out$HI2[s5:e5]+out$HI3[s5:e5]+out$HR1[s5:e5]+out$HR2[s5:e5]+out$HR3[s5:e5])
    summ3[1,3] = mean(out$HR1[s7:e7]+out$HR2[s7:e7]+out$HR3[s7:e7])/mean(out$HS1[s7:e7]+out$HS2[s7:e7]+out$HS3[s7:e7]+out$HE1[s7:e7
         ]+out$HE2[s7:e7]+out$HE3[s7:e7]+out$HI1[s7:e7]+out$HI2[s7:e7]+out$HI3[s7:e7]+out$HR1[s7:e7]+out$HR2[s7:e7]+out$HR3[s7:e7])
    summ3[2,1] = mean(out$MR1[s3:e3]+out$MR2[s3:e3]+out$MR3[s3:e3])/mean(out$MS1[s3:e3]+out$MS2[s3:e3]+out$MS3[s3:e3]+out$ME1[s3:e3
```

```
        ]+out$ME2[s3:e3]+out$ME3[s3:e3]+out$MI1[s3:e3]+out$MI2[s3:e3]+out$MI3[s3:e3]+out$MR1[s3:e3]+out$MR2[s3:e3]+out$MR3[s3:e3])
    summ3[2,2] = mean(out$MR1[s5:e5]+out$MR2[s5:e5]+out$MR3[s5:e5])/mean(out$MS1[s5:e5]+out$MS2[s5:e5]+out$MS3[s5:e5]+out$ME1[s5:e5
        ]+out$ME2[s5:e5]+out$ME3[s5:e5]+out$MI1[s5:e5]+out$MI2[s5:e5]+out$MI3[s5:e5]+out$MR1[s5:e5]+out$MR2[s5:e5]+out$MR3[s5:e5])
    summ3[2,3] = mean(out$MR1[s7:e7]+out$MR2[s7:e7]+out$MR3[s7:e7])/mean(out$MS1[s7:e7]+out$MS2[s7:e7]+out$MS3[s7:e7]+out$ME1[s7:e7
        ]+out$ME2[s7:e7]+out$ME3[s7:e7]+out$MI1[s7:e7]+out$MI2[s7:e7]+out$MI3[s7:e7]+out$MR1[s7:e7]+out$MR2[s7:e7]+out$MR3[s7:e7])
    output$summary3 = renderTable(summ3, digits = 3, rownames = T, na = "")
  }
} # if input$runIt
}) # observe
}
options(warn=0)

drawPlot4 = function(tt, g1, g2, g3, g4, ttl, y_ax1, y_ax2, legText, ps, pe, year)
{
  ps = (ps - 1)*0.1; pe = (pe - 1)*0.1
  if(pe%%30==0) pe = pe - 1
  ll = rep(c("J", "F", "M", "A", "M", "J", "J", "A", "S", "O", "N", "D"),
           (year+1))[(ps%/%30+1):(pe%/%30+1)]
  par(mar=c(5,5,5,5)); ymax1 = max(g1, g2); ymax2 = max(g3, g4)
  startYear = ps%/%360 + 1
  startMonth = c("Jan", "Feb", "Mar", "Apr", "May", "Jun", "Jul", "Aug", "Sep", "Oct", "Nov", "Dec")[(ps%%360)%/%30 + 1]
  ourXLab = paste("Month (starting ", startMonth, " year ", startYear, ")", sep = "")
  plot(tt, g1, type="l", col="blue", ylim=c(0,ymax1), ylab=y_ax1, xlab=ourXLab, xaxt = "n", main = ttl)
  lines(tt, g2, col = "green")
  par(new=TRUE)
  plot(tt, g3, type="l", col="orange", ylim=c(0,ymax2), axes = F, ylab = NA, xlab=NA)
  axis(4); mtext(y_ax2,side=4,line=3)
  axis(1, at = c(-15+30*((ps%/%30+1):(pe%/%30+1))), labels = ll)
  lines(tt, g4, col="red")
  for(i in ps:pe) if(!(i%%360)) abline(v = (i+15), lty = 2)
  legend("topright", legText, lty=c(1,1,1,1), lwd=c(2,2,2,2), col=c("blue", "green", "orange", "red"))
}

drawPlot3 = function(tt, g1, g2, g3, ttl, y_ax1, y_ax2, legText, ps, pe, year)
{
  ps = (ps - 1)*0.1; pe = (pe - 1)*0.1
  if(pe%%30==0) pe = pe - 1
  ll = rep(c("J", "F", "M", "A", "M", "J", "J", "A", "S", "O", "N", "D"),
           (year+1))[(ps%/%30+1):(pe%/%30+1)]
  par(mar=c(5,5,5,5)); ymax1 = max(g1, g2); ymax2 = max(g3)
  startYear = ps%/%360 + 1
  startMonth = c("Jan", "Feb", "Mar", "Apr", "May", "Jun", "Jul", "Aug", "Sep", "Oct", "Nov", "Dec")[(ps%%360)%/%30 + 1]
  ourXLab = paste("Month (starting ", startMonth, " year ", startYear, ")", sep = "")
  plot(tt, g1, type="l", col="blue", ylim=c(0,ymax1), ylab=y_ax1, xlab=ourXLab, xaxt = "n", main = ttl)
  lines(tt, g2, col = "green")
  par(new=TRUE)
  plot(tt, g3, type="l", col="red", ylim=c(0,ymax2), axes = F, ylab = NA, xlab=NA)
  axis(4); mtext(y_ax2,side=4,line=3)
  axis(1, at = c(-15+30*((ps%/%30+1):(pe%/%30+1))), labels = ll)
  for(i in ps:pe) if(!(i%%360)) abline(v = (i+15), lty = 2)
  legend("topright", legText, lty=c(1,1,1,1), lwd=c(2,2,2,2), col=c("blue", "green", "red"))
}

drawPlot4b = function(tt, g1, g2, g3, g4, ttl, y_ax1, y_ax2, legText, ps, pe, year)
{
  ps = (ps - 1)*0.1; ps2 = ps - 1; pe = (pe - 1)*0.1
  par(mar=c(5,5,5,5)); ymax1 = max(g1, g2); ymax2 = max(g3, g4)
  ourXLab = paste("Days since ", ps%%360%%30, " ",
                  c("Jan", "Feb", "Mar", "Apr", "May", "Jun", "Jul", "Aug", "Sep", "Oct", "Nov", "Dec")[(ps%%360)%/%30 + 1],
                  " of year ", ps%/%360 + 1, sep = "")
  plot(tt, g1, type="l", col="blue", ylim=c(0,ymax1), ylab=y_ax1, xlab=ourXLab, main = ttl)
  lines(tt, g2, col = "green")
  par(new=TRUE)
  plot(tt, g3, type="l", col="orange", ylim=c(0,ymax2), axes = F, ylab = NA, xlab=NA)
  axis(4); mtext(y_ax2,side=4,line=3)
  ps = ps - ps2; pe = pe - ps2; for(i in ps:pe) if(!(i%%360)) abline(v = i, lty = 2)
  lines(tt, g4, col="red")
  legend("topright", legText, lty=c(1,1,1,1), lwd=c(2,2,2,2), col=c("blue", "green", "orange", "red"))
}

drawPlot3b = function(tt, g1, g2, g3, ttl, y_ax1, y_ax2, legText, ps, pe, year)
{
  ps = (ps - 1)*0.1; ps2 = ps - 1; pe = (pe - 1)*0.1
  par(mar=c(5,5,5,5)); ymax1 = max(g1, g2); ymax2 = max(g3)
  ourXLab = paste("Days since ", ps%%360%%30, " ",
                  c("Jan", "Feb", "Mar", "Apr", "May", "Jun", "Jul", "Aug", "Sep", "Oct", "Nov", "Dec")[(ps%%360)%/%30 + 1],
                  " of year ", ps%/%360 + 1, sep = "")
  plot(tt, g1, type="l", col="blue", ylim=c(0,ymax1), ylab=y_ax1, xlab=ourXLab, main = ttl)
  lines(tt, g2, col = "green")
  par(new=TRUE)
  plot(tt, g3, type="l", col="red", ylim=c(0,ymax2), axes = F, ylab = NA, xlab=NA)
  axis(4); mtext(y_ax2,side=4,line=3)
  ps = ps - ps2; pe = pe - ps2; for(i in ps:pe) if(!(i%%360)) abline(v = i, lty = 2)
  legend("topright", legText, lty=c(1,1,1,1), lwd=c(2,2,2,2), col=c("blue", "green", "red"))
}

shinyApp(ui = ui, server = server)
```

Listing 2: CppFunctions.h: CppFunctions header

```
#include <Rcpp.h>
#include <math.h>
using namespace Rcpp;


// Function to print vector in cpp to check correctness
void PrinVect(NumericVector x);

// Function to replicate number equivalent to rep in Rcpp exist for NumericVector
NumericVector rep_N(long double x, int n);

// Function max of two long double
long double Sup(long double a,long double b);

// Function min of two long double
long double Inf(long double a,long double b);


// Function returning the infection rate of cattle
long double b_cattle( NumericVector x);

// Function returning the infection rate for people
long double b_people(NumericVector x);

//Function returning the proportion infection of mosquitoes
long double b_mos(NumericVector x);

//Function to perform the calculus of the rates for people, cattle and mosquitoes
NumericVector Rates_Updates(NumericVector A, NumericVector subparam);

//Function combining vectors in a list to return one vector after concatenation of the others
NumericVector combine(const List& list);

// transhumance flood shearing
NumericVector transhumance(NumericVector t,NumericVector p, double val);

// return sinusoidal function of t for dry years
NumericVector SinFun(NumericVector t, NumericVector p);

//Function with the ODEs to be solved
List ODE(NumericVector t, NumericVector state, NumericVector param);
```

Listing 3: CppFunctions.cpp: CppFunctions body

```cpp
#include <Rcpp.h>
#include <math.h>
#include <iostream>
#include <limits>

using namespace std;
using namespace Rcpp;


/* Function to print vector in cpp to check correctness */
void PrinVect(NumericVector x){
  int i=0;
  for (i = 0; i < x.size(); i++ ) {
    Rcpp::Rcout << x(i) << std::endl;
  }
}

/* Function to replicate number equivalent to rep in Rcpp exist for NumericVector */
NumericVector rep_N(long double x, int n){
  NumericVector v(rep(NumericVector::create(x),n));
  return v;
}

//[[Rcpp::export]]
/* Function max of two long double */
long double Sup(long double a,long double b)
{
  return double (a<=b ? b:a);
}

/* Function min of two long double */
long double Inf(long double a,long double b)
{
  return (a<=b ? a:b);
}


//[[Rcpp::export]]
// Function returning the infection rate of cattle
long double b_cattle( NumericVector x){
  long double a = x[0], b = x[1], c = x[2], d = x[3], e = x[4], f = x[5], g = x[6], h = x[7],  i = x[8], j = x[9], k = x[10], l = x[11], m = x[12], n = x[13];
  long double x1=-std::log (std::pow((1.0-b),(c*d/e)) * std::pow((1.0-f),(g*h/e))* std::pow((1.0-i),(j*k/e))* std::pow((1.0-l),(m*n/e)));
  x1=(std::isinf(x1) ? a : x1);
  b=(e==0.0 ? 0.0 : Inf(a,x1));

  //std::cout<<"b cattle "<<b<<" "<<x1<<" "<<a<<std::endl;
  return b;
}

//[[Rcpp::export]]
// Function returning the infection rate for people
long double b_people(NumericVector x) {
  long double a=x[0], b=x[1],  c=x[2] , d=x[3] , e=x[4], f=x[5], g=x[6], h=x[7], i=x[8], j=x[9], k=x[10], l=x[11];
  long double m=x[12],n=x[13], o=x[14], p=x[15], q=x[16], r=x[17];
  long double aa=0.0, bb=0.0;

  aa=(e==0.0 ? 0.0 : 1.0 - std::pow((1.0 - b),(c*d/e)) * std::pow((1.0-f),(g*h/e)) * std::pow((1.0-i),(j*k/e)) * std::pow((1.0-l),(m*n/e)));
  bb=(q+r==0.0 ? 0.0 : 1.0 - std::pow((1.0 - o),(p*q/(q+r))));
  long double x1 = -std::log (1.0 - (aa + bb - aa*bb));
  x1=(std::isinf(x1) ? a : x1);
  b = Inf(a,x1);
  return b;
}


//[[Rcpp::export]]
//Function returning the proportion infection of mosquitoes
long double b_mos(NumericVector x) {

  long double a = x[0], b = x[1], c = x[2], d = x[3], e = x[4], f = x[5], g = x[6];
  long double x1=(c==0.0 ? 0.0 : a*b/c)+(e+f==0.0 ? 0.0 : d*e/(e+f) )+ g;//ici b_wl is outside ifelse here
  return (x1<=1.0 ? x1 : 1.0);

}

// [[Rcpp::export]]
//Function to perform the calculus of the rates for people, cattle and mosquitoes
NumericVector Rates_Updates(NumericVector A, NumericVector subparam1, NumericVector subparam2){
  long double AS=A[0], AI=A[1], BS=A[2], BI=A[3], CS=A[4], CI=A[5], DS=A[6], DI=A[7], MI=A[8], NM=A[9], HI=A[10],NH=A[11];
  long double AA = AS + AI, BA = BS + BI, CA = CS + CI, DA = DS + DI;
  long double O_ = subparam1[0]*(AA)*subparam1[1] + subparam1[2]*(BA)*subparam1[3]+ subparam1[4]*(CA)*subparam1[5]+ subparam1[6]*(DA)*subparam1[7], chi_ = subparam1[8]*NH, temp=chi_/Sup(O_,0.000001), s_ = Inf(1.0,temp);
  long double o_ah = subparam1[0] * subparam1[1] * s_, o_bh = subparam1[2] * subparam1[3] * s_, o_ch = subparam1[4] * subparam1[5] * s_, o_dh =subparam1[6]*subparam1[7]*s_;
  long double O_m= subparam1[9]*(AA)*subparam1[1] + subparam1[10]*(BA)*subparam1[3]+subparam1[11]*(CA)*subparam1[5] + subparam1[12]*(DA)*subparam1[7], chi_m = subparam1[14]*subparam1[13]*(NM+MI);
  temp=chi_m/Sup(O_m,0.000001);
  long double  s_m = Inf(1.0,temp), o_am = subparam1[9] * subparam1[1] * s_m;
```

Listing 3 (Cont.): CppFunctions.cpp: CppFunctions body

```cpp
  long double o_bm = subparam1[10] * subparam1[3] * s_m, o_cm=subparam1[11] * subparam1[5] * s_m, o_dm=subparam1[12] * subparam1[7] * s_m;
  // o_a1 o_aa2 o_aa3
  long double o_a = o_ah + o_am, o_b = o_bh +o_bm, o_c= o_ch + o_cm, o_d= o_dh +o_dm;
  // o_a1_2 o_ab1_2 o_c1_2 o_d1_2
  long double  o_aa = subparam1[15]/Sup(0.000001,AA),o_ba = subparam1[15]/Sup(0.000001,BA), o_ca=subparam1[15]/Sup(0.000001,CA), o_da = subparam1[15]/Sup(0.000001,DA);

  NumericVector p=NumericVector::create(subparam1[16], HI, NH, subparam1[17], MI, NM, subparam2[15]);
  long double b_a = b_mos(p);
  p=NumericVector::create(subparam1[18], HI, NH, subparam1[19], MI, NM, subparam2[15]);
  long double b_b = b_mos(p);
  // b_a1 b_c1 b_d1 b_h1 b_m1
  p=NumericVector::create(subparam2[0], HI, NH, subparam2[1], MI, NM, subparam2[15]);
  long double b_c =b_mos(p);
  p=NumericVector::create(subparam2[2], HI, NH, subparam2[3], MI, NM, subparam2[15]);
  long double b_d =b_mos(p);
  //b_pe1 b_pe2 b_pe3

  p=NumericVector::create(subparam2[4], subparam2[5], o_ah, AI, NH, subparam2[6], o_bh, BI, subparam2[7], o_ch, CI,subparam2[8],o_dh,DI,subparam2[9],subparam2[10],MI,NM);
  long double b_h =(b_people(p));
  // b_ca1 b_ca2 b_ca3
  p=NumericVector::create(subparam2[4], subparam2[11], o_am, AI, NM, subparam2[12], o_bm, BI, subparam2[13], o_cm,  CI,subparam2[14], o_dm, DI);
  long double b_m =( b_cattle(p));

  NumericVector L(14);
  L=NumericVector::create(o_a, o_b, o_c, o_d, o_aa, o_ba, o_ca, o_da, b_a, b_b, b_c, b_d, b_h, b_m);
  return(L);
}

// [[Rcpp::export]]
//Function combining vectors to return one vector after concatenation of the others
NumericVector combine(const List& list)
{
  std::size_t n = list.size();

  // Figure out the length of the output vector
  std::size_t total_length = 0;
  for (std::size_t i = 0; i < n; ++i)
    total_length += Rf_length(list[i]);

  // Allocate the vector
  NumericVector output = no_init(total_length);

  // Loop and fill
  std::size_t index = 0;
  for (std::size_t i = 0; i < n; ++i)
  {
    NumericVector el = list[i];
    std::copy(el.begin(), el.end(), output.begin() + index);

    // Update the index
    index += el.size();
  }

  return output;

}


// [[Rcpp::plugins(cpp11)]]
//[[Rcpp::export]]
// transhumance flood shearing
NumericVector transhumance(NumericVector t,NumericVector p, double val, bool trans){
  NumericVector l_m(t.size());
  for(int it = 0; it != l_m.size(); ++it) {
    l_m[it]=((trans && fmod(t[it],360.0)>=(p[0]-0.4) && fmod(t[it],360.0)<=(p[0]+0.5))?p[1] : val);}
  return l_m;
  }

//[[Rcpp::export]]
// return sinusoidal function of t
NumericVector SinFun(NumericVector t, NumericVector p){
  NumericVector x(t.size());
  for(int i=0;i<t.size();i++) x[i]= (0.5 * cos(4*atan(1)*(t[i] - p[0])/p[1]) + 0.5);
  return(x);
}

//[[Rcpp::export]]
//Function with the ODEs to be solved
List ODE(NumericVector t, NumericVector state, NumericVector param){
  NumericVector ts, t_a1;
  long double b_wl = param[2];
  long double max_rate = param[0], flood_prop = param[1], O_alt = param[3], d1 = param[4], d2 = param[5], d3 = param[6], d4 = param[7], d5 = param[8], d6 = param[9], year = param[10], c0 = param[11], mmm = param[12], ds = param[13], nPeak = param[14];
  long double seasonHatch = param[15],g_h= param[16], m_h= param[17], x_h= param[18], a_h= param[19], d_h= param[20], p_mh00= param[21], f_mh1= param[22], f_mh2= param[23], f_mh3= param[24], h_h1= param[25], h_h2= param[26], h_h3= param[27];
  long double p_ha= param[28], p_hb= param[29], p_hc= param[30], p_hd= param[31], r_h= param[32], l_h12= param[33], l_h13= param[34], l_h21= param[35], l_h23= param[36], l_h31= param[37], l_h32= param[38];
  long double g_m_u= param[39], g_m_i= param[40], m_m= param[41], x_m= param[42], a_m= param[43], d_m= param[44], h_m= param[45], p_ma= param[46], p_mb= param[47], p_mc= param[48], p_md= param[49], r_m= param[50], k_m1= param[51], k_m2= param[52], k_m3= param[53];
  long double l_m13= param[54], l_m23= param[55], l_m31= param[56], l_m32= param[57],g_a= param[58], z_a= param[59], m_a= param[60],
```

Listing 3 (Cont.): CppFunctions.cpp: CppFunctions body

```
        v_a= param[61], e_ah= param[62], e_am= param[63], p_ah= param[64], p_am= param[65], k_a1= param[66], k_a2= param[67], k_a3= param[68];
  long double m_aq1= param[69], m_aq2= param[70], m_aq3= param[71], m_ap1= param[72], m_ap2= param[73], m_ap3= param[74], l_a12= param[75], l_a13= param[76], l_a21= param[77], l_a23= param[78], l_a31= param[79], l_a32= param[80];
  long double g_b= param[81], z_b= param[82], m_b= param[83], t_b= param[84], v_b= param[85], e_bh= param[86], e_bm= param[87], p_bh = param[88], p_bm= param[89], k_b1= param[90], k_b2= param[91], k_b3= param[92];
  long double m_bq1= param[93], m_bq2= param[94], m_bq3= param[95], m_bp1= param[96], m_bp2= param[97], m_bp3= param[98], l_b12= param[99], l_b13= param[100], l_b21= param[101], l_b23= param[102], l_b31= param[103], l_b32= param[104];
  long double g_c= param[105], m_c= param[106], t_c= param[107], v_c= param[108], e_ch= param[109], e_cm= param[110], p_ch= param[111], p_cm= param[112], k_c1= param[113], k_c2= param[114], k_c3= param[115], m_cp1= param[116], m_cp2= param[117], m_cp3= param[118];
  long double l_c12= param[119], l_c13= param[120], l_c21= param[121], l_c23= param[122], l_c31= param[123], l_c32= param[124];
  long double g_d= param[125], m_d= param[126], t_d= param[127], v_d= param[128], e_dh= param[129], e_dm= param[130], p_dh= param[131], p_dm= param[132], k_d1= param[133], k_d2= param[134], k_d3= param[135], m_dp1= param[136], m_dp2= param[137], m_dp3= param[138];
  long double l_d12= param[139], l_d13= param[140], l_d21= param[141], l_d23= param[142], l_d31= param[143], l_d32= param[144];
  long double shearBeg = param[146], shearEnd = param[147], shearUp = param[148];
  bool shearing= param[145], wetDry= param[149], flood= param[150], elNino= param[151], transHum = param[152], incrTrans = param[156];
  long double incrTransBeg = param[157], incrTransEnd = param[158], incrTransUp = param[159];
  long double t_a=param[153], l_m21Base=param[154],l_m12Base= param[155];
  NumericVector dry2=param[Range(160,(160+year))];
  long double HS1 = state[0], HE1 = state[1], HI1 = state[2], HR1 = state[3], HS2 = state[4], HE2 = state[5], HI2 = state[6], HR2 = state[7], HS3 = state[8], HE3 = state[9], HI3 = state[10], HR3 = state[11];
  long double MS1 = state[12], ME1 = state[13], MI1 = state[14], MR1 = state[15], MS2 =state[16], ME2 = state[17], MI2 = state[18], MR2 = state[19], MS3 = state[20], ME3 = state[21], MI3 = state[22], MR3 = state[23];
  long double AQ1 = state[24], AP1 = state[25], AS1 = state[26], AI1 = state[27], AQ2 = state[28], AP2 =state[29], AS2 =state[30], AI2 = state[31], AQ3 = state[32], AP3 = state[33], AS3 = state[34], AI3 = state[35];
  long double BQ1 = state[36], BP1 = state[37], BS1 = state[38], BI1 = state[39], BQ2 = state[40], BP2 = state[41], BS2 = state[42], BI2 = state[43], BQ3 = state[44], BP3 = state[45], BS3 = state[46], BI3 = state[47];
  long double CP1 = state[48], CS1 = state[49], CI1 = state[50], CP2 = state[51], CS2 = state[52], CI2 = state[53], CP3 = state[54], CS3 = state[55], CI3 = state[56];
  long double DP1 = state[57], DS1 = state[58], DI1 = state[59], DP2 = state[60], DS2 = state[61], DI2 = state[62], DP3 = state[63], DS3 =state[64], DI3 = state[65];

  // population sizes per compartment and per species
  //----------------  human
  long double NH1 = HS1 + HE1 + HI1 + HR1;
  long double NH2 = HS2 + HE2 + HI2 + HR2;
  long double NH3 = HS3 + HE3 + HI3 + HR3;
  //----------------  animal host
  long double NM1 = MS1 + ME1 + MR1;
  long double NM2 = MS2 + ME2 + MR2;
  long double NM3 = MS3 + ME3 + MR3;
  //----------------  vector A
  long double NA1 = AQ1 + AP1 + AS1 + AI1;
  long double NA2 = AQ2 + AP2 + AS2 + AI2;
  long double NA3 = AQ3 + AP3 + AS3 + AI3;
  //----------------  vector B
  long double  NB1 = BQ1 + BP1 + BS1 + BI1;
  long double NB2 = BQ2 + BP2 + BS2 + BI2;
  long double NB3 = BQ3 + BP3 + BS3 + BI3;
  //----------------  vector C
  long double NC1 = CP1 + CS1 + CI1;
  long double NC2 = CP2 + CS2 + CI2;
  long double NC3 = CP3 + CS3 + CI3;
  //----------------  vector D
  long double ND1 = DP1 + DS1 + DI1;
  long double ND2 = DP2 + DS2 + DI2;
  long double ND3 = DP3 + DS3 + DI3;

  // increased susceptibility of animals to vector bites (e.g. because of shearing)
  long double shear = 1.0;
  long double sB=(shearBeg-0.4), sE=(shearEnd+0.5);
  bool a=(shearing && fmod(t[0],360.0)>=sB && fmod(t[0],360.0)<=sE);
  shear = (a? shearUp :  1.0);

  // increased transmissibility of animals to people
  long double tB = (incrTransBeg - 0.4), tE = (incrTransEnd + 0.5);
  bool ta = (incrTrans && fmod(t[0], 360.0) >= tB && fmod(t[0], 360.0) <= tE);
  p_mh00 = (ta? incrTransUp : 1.0) * p_mh00;

  // transhumance
  NumericVector x=NumericVector::create(d1,max_rate);
  NumericVector l_m21 = transhumance(t,x,l_m21Base,transHum);
  x[0]= d2;
  NumericVector l_m12 = transhumance(t,x,l_m12Base,transHum);
 // wet and dry years
  int year_i= t[0]/360;
  long double dry =((wetDry && dry2[year_i] < c0)? mmm: 1.0);
  x[0] = ds, x[1]=nPeak;
  NumericVector test0=SinFun(t,x);
  ts =  dry *(seasonHatch==1? test0: rep_N(1.0,t.size()));
  // seasonal and El Nino hatching of Ae. mcintoshi eggs
  // annual flooding in march, el nino in december
  NumericVector flooded(t.size());
  NumericVector elNinoed(t.size());
  for(int i=0;i<t.size();i++){
    flooded[i]=(flood && fmod(t[i],360.0)>=(d3-0.4) && fmod(t[i],360.0)<=(d4+0.5)? 1.0 : 0.0);
    elNinoed[i]=(elNino && fmod(t[i],3600.0)>=(d5-0.4) && fmod(t[i],3600.0)<=(d6+0.5)? 1.0 : 0.0);
  }
  t_a1 = flood_prop*max_rate*flooded+ max_rate*elNinoed;
  NumericVector p1=NumericVector::create(e_ah,v_a,e_bh,v_b,e_ch,v_c,e_dh, v_d, h_h1,e_am,e_bm,e_cm, e_dm, shear,h_m, O_alt, p_ha,p_ma, p_hb, p_mb); //19 indice 20 elements
  NumericVector p2=NumericVector::create(p_hc, p_mc, p_hd, p_md, max_rate, p_ah, p_bh, p_ch, p_dh, p_mh00, f_mh1, p_am, p_bm, p_cm,
```

Listing 3 (Cont.): CppFunctions.cpp: CppFunctions body

```
        p_dm, 0.0);
   NumericVector X = NumericVector::create(AS1, AI1, BS1,  BI1, CS1, CI1, DS1,  DI1, MI1, NM1, HI1, NH1);
   // biting rates, mortality rates, infection rates floodplain
   NumericVector X1 = Rates_Updates(X, p1, p2);
   long double o_a1=X1[0],o_b1=X1[1],o_c1=X1[2], o_d1=X1[3], o_a1_2=X1[4], o_b1_2=X1[5], o_c1_2=X1[6], o_d1_2=X1[7], b_a1=X1[8],b_b1=X1[9], b_c1=X1[10], b_d1=X1[11], b_h1=X1[12], b_m1=X1[13];
   // Updates h_h1<- h_h2  and f_mh1 <- f_mh2
   p1[8]=h_h2; p2[10] = f_mh2;
   X = NumericVector::create(AS2, AI2, BS2,  BI2, CS2,  CI2, DS2,  DI2, MI2, NM2, HI2,NH2);
   // biting rates, mortality rates, infection rates floodplain
   NumericVector X2 = Rates_Updates(X, p1, p2);
   long double o_a2=X2[0],o_b2=X2[1],o_c2=X2[2], o_d2=X2[3], o_a2_2=X2[4], o_b2_2=X2[5], o_c2_2=X2[6], o_d2_2=X2[7], b_a2=X2[8],b_b2=X2[9], b_c2=X2[10], b_d2=X2[11], b_h2=X2[12], b_m2=X2[13];
   // Updates h_h2<- h_h3  and f_mh2 <- f_mh3
   p1[8]=h_h3; p2[10] = f_mh3, p2[15]=b_wl;
   X = NumericVector::create(AS3, AI3, BS3,  BI3, CS3,  CI3, DS3,  DI3, MI3, NM3, HI3,NH3);
   // biting rates, mortality rates, infection rates floodplain
   NumericVector X3 = Rates_Updates(X, p1, p2);
   long double o_a3=X3[0],o_b3=X3[1],o_c3=X3[2], o_d3=X3[3], o_a3_2=X3[4], o_b3_2=X3[5], o_c3_2=X3[6], o_d3_2=X3[7], b_a3=X3[8],b_b3=X3[9], b_c3=X3[10], b_d3=X3[11], b_h3=X3[12], b_m3=X3[13];
    // differential equations
    //---------------- human
    long double dHS1 = g_h*NH1 +  l_h21*HS2 + l_h31*HS3 + r_h*HR1 - (m_h + b_h1 +      l_h12 + l_h13)*HS1;
    long double dHE1 = b_h1*HS1 + l_h21*HE2 + l_h31*HE3 -           (m_h + x_h +       l_h12 + l_h13)*HE1;
    long double dHI1 = x_h*HE1 +  l_h21*HI2 + l_h31*HI3 -           (m_h + d_h + a_h + l_h12 + l_h13)*HI1;
    long double dHR1 = a_h*HI1 +  l_h21*HR2 + l_h31*HR3 -           (m_h + r_h +       l_h12 + l_h13)*HR1;
    long double dHS2 = g_h*NH2 +  l_h12*HS1 + l_h32*HS3 + r_h*HR2 - (m_h + b_h2 +      l_h21 + l_h23)*HS2;
    long double dHE2 = b_h2*HS2 + l_h12*HE1 + l_h32*HE3 -           (m_h + x_h +       l_h21 + l_h23)*HE2;
    long double dHI2 = x_h*HE2 +  l_h12*HI1 + l_h32*HI3 -           (m_h + d_h + a_h + l_h21 + l_h23)*HI2;
    long double dHR2 = a_h*HI2 +  l_h12*HR1 + l_h32*HR3 -           (m_h + r_h +       l_h21 + l_h23)*HR2;
    long double dHS3 = g_h*NH3 +  l_h13*HS1 + l_h23*HS2 + r_h*HR3 - (m_h + b_h3 +      l_h31 + l_h32)*HS3;
    long double dHE3 = b_h3*HS3 + l_h13*HE1 + l_h23*HE2 -           (m_h + x_h +       l_h31 + l_h32)*HE3;
    long double dHI3 = x_h*HE3 +  l_h13*HI1 + l_h23*HI2 -           (m_h + d_h + a_h + l_h31 + l_h32)*HI3;
    long double dHR3 = a_h*HI3 +  l_h13*HR1 + l_h23*HR2 -           (m_h + r_h +       l_h31 + l_h32)*HR3;
    NumericVector dH=NumericVector::create(dHS1,dHE1,dHI1,dHR1,dHS2,dHE2,dHI2,dHR2,dHS3,dHE3,dHI3,dHR3);
    //---------------- animal host
    NumericVector dMS1 = (g_m_u*NM1 + g_m_i*MI1)*Sup(0.0,1.0-NM1/k_m1) + l_m21*MS2 + l_m31*MS3 + r_m*MR1 - (m_m + b_m1      + l_m12 + l_m13)*MS1;
    NumericVector dME1 = b_m1*MS1                                       + l_m21*ME2 + l_m31*ME3           - (m_m + x_m       + l_m12 + l_m13)*ME1;
    NumericVector dMI1 = x_m*ME1                                        + l_m21*MI2 + l_m31*MI3           - (m_m + d_m + a_m + l_m12 + l_m13)*MI1;
    NumericVector dMR1 = a_m*MI1                                        + l_m21*MR2 + l_m31*MR3           - (m_m + r_m       + l_m12 + l_m13)*MR1;
    NumericVector dMS2 = (g_m_u*NM2 + g_m_i*MI2)*Sup(0.0,1.0-NM2/k_m2) + l_m12*MS1 + l_m32*MS3 + r_m*MR2 - (m_m + b_m2      + l_m21 + l_m23)*MS2;
    NumericVector dME2 = b_m2*MS2 +                                   l_m12*ME1 + l_m32*ME3 -          (m_m + x_m +       l_m21 + l_m23)*ME2;
    NumericVector dMI2 = x_m*ME2 +                                    l_m12*MI1 + l_m32*MI3 -          (m_m + d_m + a_m + l_m21 + l_m23)*MI2;
    NumericVector dMR2 = a_m*MI2 +                                    l_m12*MR1 + l_m32*MR3 -          (m_m + r_m +       l_m21 + l_m23)*MR2;
    long double   dMS3 = (g_m_u*NM3 + g_m_i*MI3)*Sup(0.0,1.0-NM3/k_m3) + l_m13*MS1 +     l_m23*MS2 + r_m*MR3 - (m_m + b_m3 +      l_m31 +     l_m32)*MS3;
    long double   dME3 = b_m3*MS3 +                                   l_m13*ME1 +     l_m23*ME2 -          (m_m + x_m +       l_m31 + l_m32)*ME3;
    long double   dMI3 = x_m*ME3 +                                    l_m13*MI1 +     l_m23*MI2 -          (m_m + d_m + a_m + l_m31 + l_m32)*MI3;
    long double   dMR3 = a_m*MI3 +                                    l_m13*MR1 +     l_m23*MR2 -          (m_m + r_m +       l_m31 + l_m32)*MR3;
    NumericVector dM=combine(List::create(dMS1,dME1,dMI1,dMR1,dMS2,dME2,dMI2,dMR2,dMS3,dME3,dMI3,dMR3));
    //---------------- vector A --- zone 1 dormant
    long double temp=1.0-NA1/k_a1;
    NumericVector dAQ1 = o_a1*g_a*Sup(0.0,temp)*z_a*AI1 -                           (m_aq1 + ts*t_a1)       *AQ1;
    NumericVector dAP1 = g_a*Sup(0.0,temp)*(o_a1*(1.0-z_a)*AI1+(o_a1+o_a1_2)*AS1) -  (m_ap1 + ts*t_a1)       *AP1;
    NumericVector dAS1 = ts*t_a1*AP1 + l_a21*AS2 + l_a31*AS3 -                  (m_a + o_a1*b_a1 + l_a12 + l_a13)*AS1;
    NumericVector dAI1 = ts*t_a1*AQ1 + o_a1*b_a1*AS1 + l_a21*AI2 + l_a31*AI3 - (m_a              + l_a12 + l_a13)*AI1;
    temp = 1.0-NA2/k_a2;
    NumericVector dAQ2 = o_a2*g_a*Sup(0.0,temp)*z_a*AI2 -                           (m_aq2 + ts*t_a)             *AQ2;
    NumericVector dAP2 = g_a*Sup(0.0,temp)*(o_a2*(1.0-z_a)*AI2+(o_a2+o_a2_2)*AS2) -  (m_ap2 + ts*t_a)             *AP2;
    NumericVector dAS2 = ts*t_a*AP2 + l_a12*AS1 + l_a32*AS3 -                       (m_a + o_a2*b_a2 + l_a21 + l_a23)*AS2;
    NumericVector dAI2 = ts*t_a*AQ2 + o_a2*b_a2*AS2 + l_a12*AI1 + l_a32*AI3 -      (m_a              + l_a21 + l_a23)*AI2;
    temp = 1.0-NA3/k_a3;
    NumericVector dAQ3 = o_a3*g_a*Sup(0.0,temp)*z_a*AI3 -                           (m_aq3 + ts*t_a)             *AQ3;
    NumericVector dAP3 = g_a*Sup(0.0,temp)*(o_a3*(1-z_a)*AI3+(o_a3+o_a3_2)*AS3) -   (m_ap3 + ts*t_a)             *AP3;
    NumericVector dAS3 = ts*t_a*AP3 + l_a13*AS1 + l_a23*AS2 -                       (m_a + o_a3*b_a3 + l_a31 + l_a32)*AS3;
    NumericVector dAI3 = ts*t_a*AQ3 + o_a3*b_a3*AS3 + l_a13*AI1 + l_a23*AI2 -      (m_a              + l_a31 + l_a32)*AI3;
    NumericVector xtemp = ts*t_a1*AP1;
    NumericVector dA = combine(List::create(dAQ1,dAP1,dAS1,dAI1,dAQ2,dAP2,dAS2,dAI2,dAQ3,dAP3,dAS3,dAI3));
    //---------------- vector B
    NumericVector dBQ1 = o_b1*g_b*Sup(0.0,1.0-NB1/k_b1)*z_b*BI1 -                          (m_bq1 + ts*t_b)            *BQ1;
    NumericVector dBP1 = g_b*Sup(0.0,1.0-NB1/k_b1)*(o_b1*(1.0-z_b)*BI1+(o_b1+o_b1_2)*BS1) - (m_bp1 + ts*t_b)             *BP1;
    NumericVector dBS1 = ts*t_b*BP1 + l_b21*BS2 + l_b31*BS3 -                       (m_b + o_b1*b_b1 + l_b12 + l_b13)*BS1;
    NumericVector dBI1 = ts*t_b*BQ1 + o_b1*b_b1*BS1 + l_b21*BI2 + l_b31*BI3 -      (m_b              + l_b12 + l_b13)*BI1;
    NumericVector dBQ2 = o_b2*g_b*Sup(0.0,1.0-NB2/k_b2)*z_b*BI2 -                          (m_bq2 + ts*t_b)            *BQ2;
    NumericVector dBP2 = g_b*Sup(0.0,1.0-NB2/k_b2)*(o_b2*(1.0-z_b)*BI2+(o_b2+o_b2_2)*BS2) - (m_bp2 + ts*t_b)             *BP2;
    NumericVector dBS2 = ts*t_b*BP2 + l_b12*BS1 + l_b32*BS3 -                       (m_b + o_b2*b_b2 + l_b21 + l_b23)*BS2;
    NumericVector dBI2 = ts*t_b*BQ2 + o_b2*b_b2*BS2 + l_b12*BI1 + l_b32*BI3 -      (m_b              + l_b21 + l_b23)*BI2;
    NumericVector dBQ3 = o_b3*g_b*Sup(0.0,1.0-NB3/k_b3)*z_b*BI3 -                          (m_bq3 + ts*t_b)            *BQ3;
    NumericVector dBP3 = g_b*Sup(0.0,1.0-NB3/k_b3)*(o_b3*(1.0-z_b)*BI3+(o_b3+o_b3_2)*BS3) - (m_bp3 + ts*t_b)             *BP3;
    NumericVector dBS3 = ts*t_b*BP3 + l_b13*BS1 + l_b23*BS2 -                       (m_b + o_b3*b_b3 + l_b31 + l_b32)*BS3;
    NumericVector dBI3 = ts*t_b*BQ3 + o_b3*b_b3*BS3 + l_b13*BI1 + l_b23*BI2 -      (m_b              + l_b31 + l_b32)*BI3;
    NumericVector dB = combine(List::create(dBQ1,dBP1,dBS1,dBI1,dBQ2,dBP2,dBS2,dBI2,dBQ3,dBP3,dBS3,dBI3));
    //---------------- vector C
    NumericVector dCP1 = g_c*Sup(0.0,1.0-NC1/k_c1)*(o_c1*CI1+(o_c1+o_c1_2)*CS1) - (m_cp1 + ts*t_c)             *CP1;
```

Listing 3 (Cont.): CppFunctions.cpp: CppFunctions body

```
    NumericVector dCS1 = ts*t_c*CP1 + l_c21*CS2 + l_c31*CS3 -             (m_c + o_c1*b_c1 + l_c12 + l_c13)*CS1;
    long double  dCI1 = o_c1*b_c1*CS1 + l_c21*CI2 + l_c31*CI3 -             (m_c              + l_c12 + l_c13)*CI1;
    NumericVector dCP2 = g_c*Sup(0.0,1.0-NC2/k_c2)*(o_c2*CI2+(o_c2+o_c2_2)*CS2) - (m_cp2 + ts*t_c)             *CP2;
    NumericVector dCS2 = ts*t_c*CP2 + l_c12*CS1 + l_c32*CS3 -             (m_c + o_c2*b_c2 + l_c21 + l_c23)*CS2;
    long double dCI2 = o_c2*b_c2*CS2 + l_c12*CI1 + l_c32*CI3 -             (m_c              + l_c21 + l_c23)*CI2;
    NumericVector dCP3 = g_c*Sup(0.0,1.0-NC3/k_c3)*(o_c3*CI3+(o_c3+o_c3_2)*CS3) - (m_cp3 + ts*t_c)             *CP3;
    NumericVector dCS3 = ts*t_c*CP3 + l_c13*CS1 + l_c23*CS2 -             (m_c + o_c3*b_c3 + l_c31 + l_c32)*CS3;
    long double dCI3 = o_c3*b_c3*CS3 + l_c13*CI1 + l_c23*CI2 -             (m_c              + l_c31 + l_c32)*CI3;
    NumericVector dC = combine(List::create(dCP1,dCS1,dCI1, dCP2,dCS2, dCI2, dCP3,dCS3,dCI3));
    //---------------- vector D
    NumericVector dDP1 = g_d*Sup(0.0,1.0-ND1/k_d1)*(o_d1*DI1+(o_d1+o_d1_2)*DS1) - (m_dp1 + ts*t_d)             *DP1;
    NumericVector dDS1 = ts*t_d*DP1 + l_d21*DS2 + l_d31*DS3 -             (m_d + o_d1*b_d1 + l_d12 + l_d13)*DS1;
    long double dDI1 = o_d1*b_d1*DS1 + l_d21*DI2 + l_d31*DI3 -             (m_d              + l_d12 + l_d13)*DI1;
    NumericVector dDP2 = g_d*Sup(0.0,1.0-ND2/k_d2)*(o_d2*DI2+(o_d2+o_d2_2)*DS2) - (m_dp2 + ts*t_d)             *DP2;
    NumericVector dDS2 = ts*t_d*DP2 + l_d12*DS1 + l_d32*DS3 -             (m_d + o_d2*b_d2 + l_d21 + l_d23)*DS2;
    long double dDI2 = o_d2*b_d2*DS2 + l_d12*DI1 + l_d32*DI3 -             (m_d              + l_d21 + l_d23)*DI2;
    NumericVector dDP3 = g_d*Sup(0.0,1.0-ND3/k_d3)*(o_d3*DI3+(o_d3+o_d3_2)*DS3) - (m_dp3 + ts*t_d)             *DP3;
    NumericVector dDS3 = ts*t_d*DP3 + l_d13*DS1 + l_d23*DS2 -             (m_d + o_d3*b_d3 + l_d31 + l_d32)*DS3;
    long double dDI3 = o_d3*b_d3*DS3 + l_d13*DI1 + l_d23*DI2 -             (m_d              + l_d31 + l_d32)*DI3;
    NumericVector dD = combine(List::create(dDP1,dDS1,dDI1,dDP2,dDS2,dDI2,dDP3,dDS3,dDI3));
    List ret=List::create(dH,dM,dA, dB, dC,dD);
    NumericVector xw = combine(ret);
    return  List::create(xw);
  }
```
